# Supplementary material for: Effectiveness of dual active ingredient insecticide-treated nets in preventing malaria: A systematic review and meta-analysis
Source: PLoS One. 2023 Aug 16;18(8):e0289469. doi: 10.1371/journal.pone.0289469 (PMC10431665; doi:10.1371/journal.pone.0289469)
Supplement: S1 File — (DOCX) [file pone.0289469.s002.docx]

Contents

[Supporting information 1 – Search Strategies 2](#_Toc134172567)

[PubMed (NCBI) 2](#_Toc134172568)

[Embase (Ovid) 3](#_Toc134172569)

[CINAHL Plus with Full Text 4](#_Toc134172570)

[Cochrane Library (Wiley) 5](#_Toc134172571)

[ClinicalTrials.gov 7](#_Toc134172572)

[WHO ICTRP 7](#_Toc134172573)

[ISRCTN 7](#_Toc134172574)

[Supporting information 2 – Studies excluded at full text 8](#_Toc134172575)

[Supporting information 3 – Characteristics of included studies 11](#_Toc134172576)

[Accrombessi 2023 11](#_Toc134172577)

[Mosha 2022 30](#_Toc134172578)

[Tiono 2018 54](#_Toc134172579)

[Supporting information 4 – Additional conducted analyses 70](#_Toc134172580)

[Supporting information 5 – ICEMAN Credibility Assessments 89](#_Toc134172581)

[Active ingredient/manufacturer subgroup 89](#_Toc134172582)

[Setting subgroup 92](#_Toc134172583)

[Vector subgroup 96](#_Toc134172584)

# Supporting information 1 – Search Strategies

| Databases | - PubMed (NCBI) - Embase (Ovid), - CINAHL with Full Text (EBSCO) - Cochrane Library (Wiley) |
| --- | --- |
| Trial Registries | - ClinicalTrials.gov, - WHO ICTRP - ISRCTN Trial Registry |
| Seed Articles | 32699237 [uid] OR 35339225 [uid] |
| Date Run | 6/7/2022 |
| PubMed (NCBI) | 3246 |
| Embase (Ovid) | 4705 |
| CINAHL with Full Text (EBSCO) | 417 |
| Cochrane Library (Wiley) | 630 |
| ClinicalTrials.gov | 220 |
| WHO ICTRP | 14 |
| ISRCTN Trial Registry | 262 |
| TOTAL RESULTS | 9494 |
| Search Created By | Carrie Price, MLS, Health Professions Librarian  Albert S. Cook Library  Towson University  Towson, MD, USA |

### PubMed (NCBI)

("malaria"[mesh] OR "black water fever"[tw] OR "black water fevers"[tw] OR "blackwater fever"[tw] OR "blackwater fevers"[tw] OR "malaria*"[tw] OR "marsh fever"[tw] OR "marsh fevers"[tw] OR "p falciparum"[tw] OR "p. falciparum"[tw] OR "paludism"[tw] OR "plasmodia"[tw] OR "plasmodial"[tw] OR "plasmodiosis"[tw] OR "plasmodium"[tw] OR "plasmodiums"[tw] OR "remittent fever"[tw] OR "remittent fevers"[tw] OR "swamp fever"[tw] OR "swamp fevers"[tw])

AND

("mosquito nets"[mesh] OR "insecticide-treated bednets"[mesh] OR "bednet"[tw] OR "bednets"[tw] OR "insecticide treated net"[tw] OR "insecticide treated nets"[tw] OR "insecticide treated bednet"[tw] OR "insecticide treated bednets"[tw] OR "insecticide treated bed net"[tw] OR "insecticide treated bed nets"[tw] OR "itn s"[tw] OR "itn"[tw] OR "itns"[tw] OR "llin*"[tw] OR "long lasting insecticidal net"[tw] OR "long lasting insecticidal nets"[tw] OR "long lasting insecticide net"[tw] OR "long lasting insecticide nets"[tw] OR "mesh"[tw] OR "meshes"[tw] OR "net"[tw] OR "nets"[tw] OR "netting"[tw])

AND

("allethrins"[mesh] OR "diflubenzuron"[mesh] OR "juvenile hormones"[mesh] OR "methoprene"[mesh] OR "permethrins"[mesh]  OR "piperonyl butoxide"[mesh] OR "pyrethrins"[mesh] OR "pyriproxyfen" [supplementary concept] OR  "2"[tiab] OR "2gard"[tw] OR "abate"[tw] OR "actellic"[tw] OR "acticin"[tw] OR "agent*"[tw] OR "allethrin*"[tw] OR "alphacypermethrin"[tw] OR "altosid"[tw] OR "ambush"[tw] OR "aqua k-othrine "[tw] OR "aqua reslin super"[tw] OR "aquatain amf"[tw] OR "armol "[tw] OR "atroban"[tw] OR "bacillus"[tw] OR "bendiocarb"[tw] OR "bifenthrin"[tw] OR "bioallethrin"[tw] OR "biomopedicul "[tw] OR "bistar 10wp"[tw] OR "chlorfenapyr*"[tw] OR "cielo ulv"[tw] OR "cispermethrin"[tw] OR "clothianidin"[tw] OR "cypermethrin*"[tw] OR "cyphenothrin"[tw] OR "dai s"[tw] OR "dai"[tw] OR "dais"[tw] OR "deltamethrin"[tw] OR "device 25wp"[tw] OR "diflubenzuron"[tw] OR "dimilin"[tw] OR "dinotefuran"[tw] OR "dioflubenzuron"[tw] OR "doubl*"[tw] OR "dronol"[tw] OR "dual"[tw] OR "dually"[tw] OR "du-dim 2 dt"[tw] OR "duranet"[tw] OR "ectiban"[tw] OR "ectomethrin "[tw] OR "elimite "[tw] OR "etofenprox"[tw] OR "expar"[tw] OR "fastm"[tw] OR "fendona"[tw]  OR "fenoxycarb"[tw] OR "ficam "[tw] OR "fludora"[tw] OR "flupyradifurone"[tw] OR "fmc 33297 "[tw] OR "fmc33297 "[tw] OR "fyfanon"[tw] OR "g2"[tw] OR "gamabenceno"[tw] OR "gamaderm"[tw] OR "gepescab "[tw] OR "gokilaht-s 5ec "[tw] OR "icon"[tw] OR "iconlife"[tw] OR "ig2"[tw] OR "imidacloprid"[tw] OR "infectomite "[tw] OR "infectopedicul"[tw] OR "insect growth regulator*"[tw] OR "interceptor "[tw] OR "interceptor g2"[tw] OR "interceptorg2 "[tw] OR "ipevet"[tw] OR "ipevetex"[tw] OR "juvenile hormone*"[tw] OR "k othrine"[tw] OR "kinoprene"[tw] OR "klinits"[tw] OR "klypson 500 wg"[tw] OR "kothrine"[tw] OR "lambda cyhalothrin"[tw] OR "lambdacyhalothrin"[tw] OR "limitor 5 gr"[tw] OR "loxazol"[tw] OR "lyclear "[tw] OR "magnet"[tw] OR "malathion"[tw] OR "methoprene"[tw] OR "miranet"[tw] OR "mosquiron 100ec"[tw] OR "mozkill"[tw] OR "nedax plus"[tw] OR "net protect"[tw] OR "netprotect"[tw] OR "new nok"[tw] OR "nix"[tw] OR "nonpyrethroid*"[tw] OR "novaluron"[tw] OR "novo herklin"[tw] OR "nrdc 143"[tw] OR "nrdc 147 "[tw] OR "olyset"[tw] OR "olysetnet"[tw] OR "olysetplus"[tw] OR "oms 1821"[tw] OR "pali 250 wg"[tw] OR "panda"[tw] OR “pandanet”[tw] OR "pbo"[tw] OR "pdms"[tw]  OR "pendulum"[tw] OR "perigen"[tw] OR "permanet"[tw]  OR "permanone "[tw] OR "permectrin"[tw] OR "permethrin*"[tw] OR "permicren"[tw] OR "permite"[tw] OR "piopel"[tw] OR "piperonyl butoxide*"[tw] OR "pirimiphos methyl"[tw] OR "pirimiphosmethyl"[tw] OR "polydimethylsiloxane"[tw] OR "pounce"[tw] OR "pp 557"[tw] OR "prallethrin"[tw] OR "pyrethrin*"[tw] OR "pyrethroid*"[tw] OR "pyriproxifen"[tw] OR "pyriproxyfen"[tw] OR "quellada−p"[tw] OR "reliefnet"[tw] OR "relief net"[tw] OR "revival"[tw] OR "rg"[tw] OR "rg2"[tw] OR "royal gaurd"[tw] OR "royal guard"[tw] OR "royal sentry"[tw] OR "rubi"[tw] OR "safe net"[tw] OR "safenet"[tw] OR "sarcop"[tw] OR "scabianil "[tw] OR "sentrin"[tw] OR "spinosad"[tw] OR "stockade"[tw] OR "stockate"[tw] OR "sumilarv 0.5g "[tw] OR "sumilarv 2mr"[tw] OR "sumishield 50wg"[tw] OR "temeguard"[tw] OR "temephos"[tw] OR "transfluthrin"[tw] OR "transpermethrin"[tw] OR "tsara"[tw] OR "twin"[tw] OR "two"[tiab] OR "vectobac"[tw] OR "vectomax"[tw] OR "vectra felis"[tw] OR "vectron"[tw] OR "veeralin"[tw] OR "wl 43479"[tw] OR "yahe ln"[tw] OR "yorkool"[tw] OR "zalvor "[tw] OR "zehu ze "[tw] OR "zehuze"[tw] OR "zr 777"[tw])

### Embase (Ovid)

| 1 | malaria/ | 77008 |
| --- | --- | --- |
| 2 | (black water fever or black water fevers or blackwater fever or blackwater fevers or malaria* or marsh fever or marsh fevers or p falciparum or p falciparum or paludism or plasmodia or plasmodial or plasmodiosis or plasmodium or plasmodiums or remittent fever or remittent fevers or swamp fever or swamp fevers).mp. | 142794 |
| 3 | 1 or 2 | 142794 |
| 4 | bed net/ or insecticide treated net/ | 6085 |
| 5 | (bednet OR bednets OR insecticide treated net OR insecticide treated nets OR insecticide treated bednet OR insecticide treated bednets OR insecticide treated bed net OR insecticide treated bed nets OR itn s OR itn OR itns OR llin* OR long lasting insecticidal net OR long lasting insecticidal nets OR long lasting insecticide net OR long lasting insecticide nets OR mesh OR meshes OR net OR nets OR netting).mp. | 245605 |
| 6 | 4 or 5 | 245605 |
| 7 | allethrin/ or diflubenzuron/ or fenoxycarb/ or juvenile hormone/ or kinoprene/or permethrin/ or piperonyl butoxide/ or pyriproxyfen/ or pyrethrin/ or pyrethroid/ | 19450 |
| 8 | ('2' or two).ti,ab. or (2gard or abate or actellic or acticin or agent* or allethrin* or alphacypermethrin or altosid or ambush or aqua k-othrine or aqua reslin super or aquatain amf or armol or atroban or bacillus or bendiocarb or bifenthrin or bioallethrin or biomopedicul or bistar 10wp or chlorfenapyr* or cielo ulv or cispermethrin or clothianidin or cypermethrin* or cyphenothrin or dai s or dai or dais or deltamethrin or device 25wp or diflubenzuron or dimilin or dinotefuran or dioflubenzuron or doubl* or dronol or dual or dually or du-dim 2 dt or duranet or ectiban or ectomethrin or elimite or etofenprox or expar or fastm or fendona or fenoxycarb or ficam or fludora or flupyradifurone or fmc 33297 or fmc33297 or fyfanon or g2 or gamabenceno or gamaderm or gepescab or gokilaht-s 5ec or icon or iconlife or ig2 or imidacloprid or infectomite or infectopedicul or insect growth regulator* or interceptor or interceptor g2 or interceptorg2 or ipevet or ipevetex or juvenile hormone* or k othrine or kinoprene or klinits or klypson 500 wg or kothrine or lambda cyhalothrin or lambdacyhalothrin or limitor 5 gr or loxazol or lyclear or magnet or malathion or methoprene or miranet or mosquiron 100ec or mozkill or nedax plus or net protect or netprotect or new nok or nix or nonpyrethroid* or novaluron or novo herklin or nrdc 143 or nrdc 147 or olyset or olysetnet or olysetplus or oms 1821 or pali 250 wg or panda or pandanet or pbo or pdms or pendulum or perigen or permanet or permanone or permectrin or permethrin* or permicren or permite or piopel or piperonyl butoxide* or pirimiphos methyl or pirimiphosmethyl or polydimethylsiloxane or pounce or pp 557 or prallethrin or pyrethrin* or pyrethroid* or pyriproxifen or pyriproxyfen or quellada p or reliefnet or relief net or revival or rg or rg2 or royal gaurd or royal guard or royal sentry or rubi or safe net or safenet or sarcop or scabianil or sentrin or spinosad or stockade or stockate or sumilarv or sumilarv 2mr or sumishield 50wg or temeguard or temephos or transfluthrin or transpermethrin or tsara or twin or vectobac or vectomax or vectra felis or vectron or veeralin or wl 43479 or yahe ln or yorkool or zalvor or zehu ze or zehuze or zr 777).mp. | 15940831 |
| 9 | 7 or 8 | 15940831 |
| 10 | 3 and 6 and 9 | 4705 |

### CINAHL Plus with Full Text

(MH "malaria" OR "black water fever" OR "black water fevers" OR "blackwater fever" OR "blackwater fevers" OR "malaria*" OR "marsh fever" OR "marsh fevers" OR "p falciparum" OR "p. falciparum" OR "paludism" OR "plasmodia" OR "plasmodial" OR "plasmodiosis" OR "plasmodium" OR "plasmodiums" OR "remittent fever" OR "remittent fevers" OR "swamp fever" OR "swamp fevers")

AND

(MH "mosquito nets" OR "bednet" OR "bednets" OR "insecticide treated net" OR "insecticide treated nets" OR "insecticide treated bednet" OR "insecticide treated bednets" OR "insecticide treated bed net" OR "insecticide treated bed nets" OR "itn s" OR "itn" OR "itns" OR "llin*" OR "long lasting insecticidal net" OR "long lasting insecticidal nets" OR "long lasting insecticide net" OR "long lasting insecticide nets" OR "mesh" OR "meshes" OR "net" OR "nets" OR "netting")

AND

("pyriproxyfen" OR  "2" OR "2gard" OR "abate" OR "actellic" OR "acticin" OR "agent*" OR "allethrin*" OR "alphacypermethrin" OR "altosid" OR "ambush" OR "aqua k-othrine " OR "aqua reslin super" OR "aquatain amf" OR "armol " OR "atroban" OR "bacillus" OR "bendiocarb" OR "bifenthrin" OR "bioallethrin" OR "biomopedicul " OR "bistar 10wp" OR "chlorfenapyr*" OR "cielo ulv" OR "cispermethrin" OR "clothianidin" OR "cypermethrin*" OR "cyphenothrin" OR "dai s" OR "dai" OR "dais" OR "deltamethrin" OR "device 25wp" OR "diflubenzuron" OR "dimilin" OR "dinotefuran" OR "dioflubenzuron" OR "doubl*" OR "dronol" OR "dual" OR "dually" OR "du-dim 2 dt" OR "duranet" OR "ectiban" OR "ectomethrin " OR "elimite " OR "etofenprox" OR "expar" OR "fastm" OR "fendona"  OR "fenoxycarb" OR "ficam " OR "fludora" OR "flupyradifurone" OR "fmc 33297 " OR "fmc33297 " OR "fyfanon" OR "g2" OR "gamabenceno" OR "gamaderm" OR "gepescab " OR "gokilaht-s 5ec " OR "icon" OR "iconlife" OR "ig2" OR "imidacloprid" OR "infectomite " OR "infectopedicul" OR "insect growth regulator*" OR "interceptor " OR "interceptor g2" OR "interceptorg2 " OR "ipevet" OR "ipevetex" OR "juvenile hormone*" OR "k othrine" OR "kinoprene" OR "klinits" OR "klypson 500 wg" OR "kothrine" OR "lambda cyhalothrin" OR "lambdacyhalothrin" OR "limitor 5 gr" OR "loxazol" OR "lyclear " OR "magnet" OR "malathion" OR "methoprene" OR "miranet" OR "mosquiron 100ec" OR "mozkill" OR "nedax plus" OR "net protect" OR "netprotect" OR "new nok" OR "nix" OR "nonpyrethroid*" OR "novaluron" OR "novo herklin" OR "nrdc 143" OR "nrdc 147 " OR "olyset" OR "olysetnet" OR "olysetplus" OR "oms 1821" OR "pali 250 wg" OR "panda" OR “pandanet” OR "pbo" OR "pdms"  OR "pendulum" OR "perigen" OR "permanet"  OR "permanone " OR "permectrin" OR "permethrin*" OR "permicren" OR "permite" OR "piopel" OR "piperonyl butoxide*" OR "pirimiphos methyl" OR "pirimiphosmethyl" OR "polydimethylsiloxane" OR "pounce" OR "pp 557" OR "prallethrin" OR "pyrethrin*" OR "pyrethroid*" OR "pyriproxifen" OR "pyriproxyfen" OR "quellada−p" OR "reliefnet" OR "relief net" OR "revival" OR "rg" OR "rg2" OR "royal gaurd" OR "royal guard" OR "royal sentry" OR "rubi" OR "safe net" OR "safenet" OR "sarcop" OR "scabianil " OR "sentrin" OR "spinosad" OR "stockade" OR "stockate" OR "sumilarv 0.5g " OR "sumilarv 2mr" OR "sumishield 50wg" OR "temeguard" OR "temephos" OR "transfluthrin" OR "transpermethrin" OR "tsara" OR "twin" OR "two" OR "vectobac" OR "vectomax" OR "vectra felis" OR "vectron" OR "veeralin" OR "wl 43479" OR "yahe ln" OR "yorkool" OR "zalvor " OR "zehu ze " OR "zehuze" OR "zr 777")

### Cochrane Library (Wiley)

([mh "malaria"] OR "black water fever":ti,ab,kw OR "black water fevers":ti,ab,kw OR "blackwater fever":ti,ab,kw OR "blackwater fevers":ti,ab,kw OR "malaria*":ti,ab,kw OR "marsh fever":ti,ab,kw OR "marsh fevers":ti,ab,kw OR "p falciparum":ti,ab,kw OR "p. falciparum":ti,ab,kw OR "paludism":ti,ab,kw OR "plasmodia":ti,ab,kw OR "plasmodial":ti,ab,kw OR "plasmodiosis":ti,ab,kw OR "plasmodium":ti,ab,kw OR "plasmodiums":ti,ab,kw OR "remittent fever":ti,ab,kw OR "remittent fevers":ti,ab,kw OR "swamp fever":ti,ab,kw OR "swamp fevers":ti,ab,kw)

AND

([mh "mosquito nets"] OR [mh "insecticide-treated bednets"] OR "bednet":ti,ab,kw OR "bednets":ti,ab,kw OR "insecticide treated net":ti,ab,kw OR "insecticide treated nets":ti,ab,kw OR "insecticide treated bednet":ti,ab,kw OR "insecticide treated bednets":ti,ab,kw OR "insecticide treated bed net":ti,ab,kw OR "insecticide treated bed nets":ti,ab,kw OR "itn s":ti,ab,kw OR "itn":ti,ab,kw OR "itns":ti,ab,kw OR "llin*":ti,ab,kw OR "long lasting insecticidal net":ti,ab,kw OR "long lasting insecticidal nets":ti,ab,kw OR "long lasting insecticide net":ti,ab,kw OR "long lasting insecticide nets":ti,ab,kw OR "mesh":ti,ab,kw OR "meshes":ti,ab,kw OR "net":ti,ab,kw OR "nets":ti,ab,kw OR "netting":ti,ab,kw)

AND

([mh "allethrins"] OR [mh "diflubenzuron"] OR [mh "juvenile hormones"] OR [mh "methoprene"] OR [mh "permethrins"]  OR [mh "piperonyl butoxide"] OR [mh "pyrethrins"] OR  "2":ti,ab OR "2gard":ti,ab,kw OR "abate":ti,ab,kw OR "actellic":ti,ab,kw OR "acticin":ti,ab,kw OR "agent*":ti,ab,kw OR "allethrin*":ti,ab,kw OR "alphacypermethrin":ti,ab,kw OR "altosid":ti,ab,kw OR "ambush":ti,ab,kw OR "aqua k-othrine ":ti,ab,kw OR "aqua reslin super":ti,ab,kw OR "aquatain amf":ti,ab,kw OR "armol ":ti,ab,kw OR "atroban":ti,ab,kw OR "bacillus":ti,ab,kw OR "bendiocarb":ti,ab,kw OR "bifenthrin":ti,ab,kw OR "bioallethrin":ti,ab,kw OR "biomopedicul ":ti,ab,kw OR "bistar 10wp":ti,ab,kw OR "chlorfenapyr*":ti,ab,kw OR "cielo ulv":ti,ab,kw OR "cispermethrin":ti,ab,kw OR "clothianidin":ti,ab,kw OR "cypermethrin*":ti,ab,kw OR "cyphenothrin":ti,ab,kw OR "dai s":ti,ab,kw OR "dai":ti,ab,kw OR "dais":ti,ab,kw OR "deltamethrin":ti,ab,kw OR "device 25wp":ti,ab,kw OR "diflubenzuron":ti,ab,kw OR "dimilin":ti,ab,kw OR "dinotefuran":ti,ab,kw OR "dioflubenzuron":ti,ab,kw OR "doubl*":ti,ab,kw OR "dronol":ti,ab,kw OR "dual":ti,ab,kw OR "dually":ti,ab,kw OR "du-dim 2 dt":ti,ab,kw OR "duranet":ti,ab,kw OR "ectiban":ti,ab,kw OR "ectomethrin ":ti,ab,kw OR "elimite ":ti,ab,kw OR "etofenprox":ti,ab,kw OR "expar":ti,ab,kw OR "fastm":ti,ab,kw OR "fendona":ti,ab,kw  OR "fenoxycarb":ti,ab,kw OR "ficam ":ti,ab,kw OR "fludora":ti,ab,kw OR "flupyradifurone":ti,ab,kw OR "fmc 33297 ":ti,ab,kw OR "fmc33297 ":ti,ab,kw OR "fyfanon":ti,ab,kw OR "g2":ti,ab,kw OR "gamabenceno":ti,ab,kw OR "gamaderm":ti,ab,kw OR "gepescab ":ti,ab,kw OR "gokilaht-s 5ec ":ti,ab,kw OR "icon":ti,ab,kw OR "iconlife":ti,ab,kw OR "ig2":ti,ab,kw OR "imidacloprid":ti,ab,kw OR "infectomite ":ti,ab,kw OR "infectopedicul":ti,ab,kw OR "insect growth regulator*":ti,ab,kw OR "interceptor ":ti,ab,kw OR "interceptor g2":ti,ab,kw OR "interceptorg2 ":ti,ab,kw OR "ipevet":ti,ab,kw OR "ipevetex":ti,ab,kw OR "juvenile hormone*":ti,ab,kw OR "k othrine":ti,ab,kw OR "kinoprene":ti,ab,kw OR "klinits":ti,ab,kw OR "klypson 500 wg":ti,ab,kw OR "kothrine":ti,ab,kw OR "lambda cyhalothrin":ti,ab,kw OR "lambdacyhalothrin":ti,ab,kw OR "limitor 5 gr":ti,ab,kw OR "loxazol":ti,ab,kw OR "lyclear ":ti,ab,kw OR "magnet":ti,ab,kw OR "malathion":ti,ab,kw OR "methoprene":ti,ab,kw OR "miranet":ti,ab,kw OR "mosquiron 100ec":ti,ab,kw OR "mozkill":ti,ab,kw OR "nedax plus":ti,ab,kw OR "net protect":ti,ab,kw OR "netprotect":ti,ab,kw OR "new nok":ti,ab,kw OR "nix":ti,ab,kw OR "nonpyrethroid*":ti,ab,kw OR "novaluron":ti,ab,kw OR "novo herklin":ti,ab,kw OR "nrdc 143":ti,ab,kw OR "nrdc 147 ":ti,ab,kw OR "olyset":ti,ab,kw OR "olysetnet":ti,ab,kw OR "olysetplus":ti,ab,kw OR "oms 1821":ti,ab,kw OR "pali 250 wg":ti,ab,kw OR "panda":ti,ab,kw OR “pandanet”:ti,ab,kw OR "pbo":ti,ab,kw OR "pdms":ti,ab,kw  OR "pendulum":ti,ab,kw OR "perigen":ti,ab,kw OR "permanet":ti,ab,kw  OR "permanone ":ti,ab,kw OR "permectrin":ti,ab,kw OR "permethrin*":ti,ab,kw OR "permicren":ti,ab,kw OR "permite":ti,ab,kw OR "piopel":ti,ab,kw OR "piperonyl butoxide*":ti,ab,kw OR "pirimiphos methyl":ti,ab,kw OR "pirimiphosmethyl":ti,ab,kw OR "polydimethylsiloxane":ti,ab,kw OR "pounce":ti,ab,kw OR "pp 557":ti,ab,kw OR "prallethrin":ti,ab,kw OR "pyrethrin*":ti,ab,kw OR "pyrethroid*":ti,ab,kw OR "pyriproxifen":ti,ab,kw OR "pyriproxyfen":ti,ab,kw OR "quellada p":ti,ab,kw OR "reliefnet":ti,ab,kw OR "relief net":ti,ab,kw OR "revival":ti,ab,kw OR "rg":ti,ab,kw OR "rg2":ti,ab,kw OR "royal gaurd":ti,ab,kw OR "royal guard":ti,ab,kw OR "royal sentry":ti,ab,kw OR "rubi":ti,ab,kw OR "safe net":ti,ab,kw OR "safenet":ti,ab,kw OR "sarcop":ti,ab,kw OR "scabianil ":ti,ab,kw OR "sentrin":ti,ab,kw OR "spinosad":ti,ab,kw OR "stockade":ti,ab,kw OR "stockate":ti,ab,kw OR "sumilarv 0.5g ":ti,ab,kw OR "sumilarv 2mr":ti,ab,kw OR "sumishield 50wg":ti,ab,kw OR "temeguard":ti,ab,kw OR "temephos":ti,ab,kw OR "transfluthrin":ti,ab,kw OR "transpermethrin":ti,ab,kw OR "tsara":ti,ab,kw OR "twin":ti,ab,kw OR "two":ti,ab OR "vectobac":ti,ab,kw OR "vectomax":ti,ab,kw OR "vectra felis":ti,ab,kw OR "vectron":ti,ab,kw OR "veeralin":ti,ab,kw OR "wl 43479":ti,ab,kw OR "yahe ln":ti,ab,kw OR "yorkool":ti,ab,kw OR "zalvor ":ti,ab,kw OR "zehu ze ":ti,ab,kw OR "zehuze":ti,ab,kw OR "zr 777":ti,ab,kw)

### ClinicalTrials.gov

| Malaria AND net | 95 |
| --- | --- |
| Malaria AND bednet | 57 |
| Malaria AND LLIN | 31 |
| Malaria AND ITN | 37 |
| TOTAL | 220 |

Search terms are automatically truncated. "net" returns "nets".

### WHO ICTRP

| Malaria (condition) AND net (intervention) | 0 |
| --- | --- |
| Malaria (condition) AND nets (intervention) | 6 |
| Malaria (condition) AND bednet (intervention) | 1 |
| Malaria (condition) AND bednets (intervention) | 0 |
| Malaria (condition) AND LLIN (intervention) | 4 |
| Malaria (condition) AND LLINs (intervention) | 2 |
| Malaria (condition) AND ITN (intervention) | 1 |
| Malaria (condition) AND ITNs (intervention) | 0 |
| TOTAL | 14 |

### ISRCTN

Malaria

262

# Supporting information 2 – Studies excluded at full text

| **Title** | **Authors** | **Exclusion Reason** |
| --- | --- | --- |
| Department of Error |  | Wrong study design |
| Does IPTi decrease malaria morbidity but not mortality? Christine Stabell Benn1 | Aaby, P. | Wrong study design |
| Bio-efficacy of new long-lasting insecticide-treated bed nets against Anopheles funestus and Anopheles gambiae from central and northern Mozambique | Abilio, A. P.; Marrune, P.; De Deus, N.; Mbofana, F.; Muianga, P.; Kampango, A. | Wrong outcomes |
| Effect of insecticide-treated bednets for malaria control in Southeast Anatolia-Turkey | Alten, B.; Caglar, S. S.; Simsek, F. M.; Kaynas, S. | Wrong intervention |
| The effectiveness of permethrin-impregnated bed nets for malaria control in Kg. Ganoh, an Orang Asli area of Rompin District, Pahang | Amal, N. M.; Yussof, S. | Wrong study design |
| Long-term use of insecticide treated bed nets reduces malaria transmission and death rates in children | Anonymous, | Wrong intervention |
| Mosquito nets protect children from malaria | Anonymous, | Wrong study design |
| A cluster randomized controlled cross-over bed net acceptability and preference trial in Solomon Islands: Community participation in shaping policy for malaria elimination | Atkinson, J. A.; Bobogare, A.; Vallely, A.; Boaz, L.; Kelly, G.; Basifiri, W.; Forsyth, S.; Baker, P.; Appleyard, B.; Toaliu, H.; Williams, G. | Wrong intervention |
| Pyrethroid resistance in sub-Saharan Africa | Bajunirwe, Francis | Wrong study design |
| Evaluation of Interceptor long-lasting insecticidal nets in eight communities in Liberia | Banek, K.; Kilian, A.; Allan, R. | Wrong intervention |
| Seeing (RED) | Benn, C. | Wrong study design |
| Malaria elimination: Worthy, challenging, and just possible | Das, P.; Horton, R. | Wrong study design |
| Insecticide treated nets - Technological & operational challenges | Dash, A. P.; Yadav, R. S. | Wrong study design |
| Randomised trials in child health in developing countries 2011 | Duke, T. | Wrong study design |
| Bed nets prove their mettle against malaria | Enserink, M. | Wrong study design |
| Use of insecticide-treated nets by pregnant and childbearing-age women: Action research in Southern Nigeria | Esienumoh, Ekpoanwan; Mboho, Margaret; Ndiok, Akon | Wrong study design |
| Pyriproxyfen-treated bed nets reduce reproductive fitness and longevity of pyrethroid-resistant Anopheles gambiae under laboratory and field conditions | Grisales, N.; Lees, R. S.; Maas, J.; Morgan, J. C.; Wangrawa, D. W.; Guelbeogo, W. M.; N'Fale, S.; Lindsay, S. W.; McCall, P. J.; Ranson, H. | Wrong outcomes |
| Evidence supporting deployment of next generation insecticide treated nets in Burkina Faso: bioassays with either chlorfenapyr or piperonyl butoxide increase mortality of pyrethroid-resistant Anopheles gambiae | Hien, A. S.; Soma, D. D.; Maiga, S.; Coulibaly, D.; Diabate, A.; Belemvire, A.; Diouf, M. B.; Jacob, D.; Kone, A.; Dotson, E.; Awolola, T. S.; Oxborough, R. M.; Dabire, R. K. | Wrong outcomes |
| Control of malaria vectors and management of insecticide resistance through universal coverage with next-generation insecticide-treated nets | Killeen, G. F. | Wrong study design |
| Malaria nets shape up for resistance | Lines, J. | Wrong study design |
| Insecticide products: Treatment of mosquito nets at home | Lines, J. D.; Zaim, M. | Wrong study design |
| Comparative functional survival and equivalent annual cost of 3 long-lasting insecticidal net (LLIN) products in Tanzania: A randomised trial with 3-year follow up | Lorenz, L. M.; Bradley, J.; Yukich, J.; Massue, D. J.; Mboma, Z. M.; Pigeon, O.; Moore, J.; Kilian, A.; Lines, J.; Kisinza, W.; Overgaard, H. J.; Moore, S. J. | Wrong intervention |
| Life shortening effect of Olyset Duo, a longlasting insecticidal net incorporating a mixture of pyrethroid and pyriproxyfen, against pyrethroidresistant mosquito | Lucas, J. R.; Invest, J.; Ohashi, K.; Teshima, H.; Shono, Y. | Wrong intervention |
| Comparing the durability of the long-lasting insecticidal nets DawaPlus 2.0 and DuraNet© in northwest Democratic Republic of Congo | Mansiangi, P.; Umesumbu, S.; Etewa, I.; Zandibeni, J.; Bafwa, N.; Blaufuss, S.; Olapeju, B.; Ntoya, F.; Sadou, A.; Irish, S.; Mukomena, E.; Kalindula, L.; Watsenga, F.; Akogbeto, M.; Babalola, S.; Koenker, H.; Kilian, A. | Wrong study design |
| Durability of three types of dual active ingredient long-lasting insecticidal net compared to a pyrethroid-only LLIN in Tanzania: methodology for a prospective cohort study nested in a cluster randomized controlled trial | Martin, J. L.; Messenger, L. A.; Mosha, F. W.; Lukole, E.; Mosha, J. F.; Kulkarni, M.; Churcher, T. S.; Sherrard-Smith, E.; Manjurano, A.; Protopopoff, N.; Rowland, M. | Wrong outcomes |
| Effectiveness of two types of long lasting insecticidal nets after two years of use for malaria vector control in an area of high pyrethroid resistance, muleba -tanzania | Martine, J. L.; Protopopof, N.; Magesa, S.; Mosha, F. W. | Wrong study design |
| Evaluation of next generation of insecticide treated nets: the Tanzanian experiences | Mosha, J. F.; Lukole, E.; Mosha, F. W.; Manjurano, A.; Martin, J.; Kulkarni, M.; Mwalimu, C. D.; Rowland, M.; Protopopoff, N. | Wrong study design |
| Estimating the Malaria Prevention Impact of New Nets: Observational Analyses to Evaluate the Evidence Generated During Piloted New Net Distributions in Rwanda | PATH; Rwanda Biomedical Centre; University of Rwanda; Liverpool School of Tropical Medicine | Wrong study design |
| Estimating the Malaria Prevention Impact of New Nets: Observational Analyses to Evaluate the Evidence Generated During Piloted New Net Distributions in Mozambique | PATH; Tropical Health LLP; Ministry of Health, Mozambique; Instituto Nacional de Saúde; Tulane University; Liverpool School of Tropical Medicine | Wrong study design |
| A new promising long lasting insecticide net in the control of insecticide resistant vectors: Olyset Duo: A pyriproxyfen and permethrin mixture net | Protopopoff, N.; Oxborough, R.; Irish, S.; Malone, D.; Mosha, F. W.; Rowland, M. | Wrong study design |
| Accelerating the evidence for new classes of long-lasting insecticide-treated nets | Protopopoff, N.; Rowland, M. | Wrong study design |
| Durability, household usage and washing pattern of DuraNet(©) and Interceptor(®) long-lasting insecticidal nets in long-term field trials in India | Sharma, S. K.; Yadav, R. S.; Srivastava, H. C.; Bhatt, R. M.; Pant, C. S.; Haque, M. A.; Sreehari, U.; Raghavendra, K. | Wrong intervention |
| The impact of Olyset net and DawaPlus 2.0 on the risk of plasmodium infection in Gembe East, Western Kenya | Tamari, N.; Sonye, G. O.; Awuor, B.; Kongere, J. O.; Hashimoto, M.; Kataoka, M.; Munga, S.; Minakawa, N. | Wrong outcomes |
| Erratum: Assessing the impact of the addition of pyriproxyfen on the durability of permethrin-treated bed nets in Burkina Faso: A compound-randomized controlled trial (Malaria Journal (2019) 8 (383) DOI: 10.1186/s12936-019-3018-1) | Toe, K. H.; Mechan, F.; Tangena, J. A. A.; Morris, M.; Solino, J.; Tchicaya, E. F. S.; Traore, A.; Ismail, H.; Maas, J.; Lissenden, N.; Pinder, M.; Lindsay, S. W.; Tiono, A. B.; Ranson, H.; Sagnon, N. | Wrong outcomes |
| Assessing the impact of the addition of pyriproxyfen on the durability of permethrin-treated bed nets in Burkina Faso: A compound-randomized controlled trial | Toe, K. H.; Mechan, F.; Tangena, J. A. A.; Morris, M.; Solino, J.; Tchicaya, E. F. S.; Traore, A.; Ismail, H.; Maas, J.; Lissenden, N.; Pinder, M.; Lindsay, S. W.; Tiono, A. B.; Ranson, H.; Sagnon, N. | Wrong outcomes |
| Effectiveness of a long-lasting insecticide treatment kit (ICON® Maxx) for polyester nets over three years of household use: a WHO phase III trial in Tanzania | Tungu, P. K.; Sudi, W.; Kisinza, W.; Rowland, M. | Wrong outcomes |

# Supporting information 3 – Characteristics of included studies

## Accrombessi 2023

| **Study: Assessing the efficacy of two dual-active ingredients long-lasting insecticidal nets for the control of malaria transmitted by pyrethroid-resistant vectors in Benin: study protocol for a three-arm, single-blinded, parallel, cluster-randomized controlled trial**  **AND**  **Preliminary data collected from direct correspondence with the authors**  ***Reviewer note, all of this methodological detail has been collected from the protocol, and is presented in the future tense as appropriate. Details of methods actually conducted were not provided by the authors*** | |
| --- | --- |
| **DESIGN** | |
| **Lead Author** | Manfred Accrombessi |
| **Publication year** | Protocol published in 2021, publication of results in 2023 |
| **Trial name** | Part of a larger project called “The new net project” |
| **Other reports of this study**  (i.e., author, year, title, doi) | Protocol – Accrombessi (2021)  Final results to be published at later date (2023) |
| **Time period study was conducted** | Intervention distribution occurred in March 2020. Outcomes data is still being collected, however outcomes reported by the authors are up to 20-months post-intervention. |
| **Design** | Three-arm superiority, cluster-randomised controlled trial |
| **Eligibility criteria of participants** | The cohort will include children aged 6 months to 9 years old resident in the study villages, without any severe illnesses and whose parents/carers have given written informed consent for their child to be included in the study. The participant also needed to be a resident of the study area for the past three months to be eligible to contribute data via the cross-sectional surveys.  Age-stratified individuals are randomly selected from each cluster using the census list generated during the registration activity. A maximum of two household members will be selected at random, using a random number table.  The inclusion criteria are:   - For adults: willing to participate and provide consent - For children: Having an adult caregiver willing to provide written consent for the household and clinical survey and assent for children over 10. - Residence in the village over the last 3 months   Exclusion criteria   - Dwelling not found or vacant during the survey - No adult caregiver capable to give informed consent - Habitants severely ill |
| **Recruitment methods and rate** | Using satellite map we identified and geo-located 60 villages within the district that are accessible and within a travelling time from the office in Cove town of less than 2 hours’ drive and ideally at a distance of minimum 2 kilometers from each other. Each cluster will be comprised of 1 hamlet/ village for a total number of houses of 200 household (1200 residents) per cluster.  The selection of the 60 clusters for the main study will be based on the following criteria determined from the baseline cross-sectional survey:   1. Hamlet leader and population acceptance 2. Within 2 hours drive from Cove Office 3. Clusters with estimated malaria infection prevalence over 30% (from baseline cross sectional survey) 4. Clusters minimum 2 km apart   To estimate malaria case incidence, a cohort of 25 children per cluster aged 6 months to 9 years will be randomly selected from a census list of children living in the study clusters and followed over 24 months. For malaria infection prevalence assessment, two cross-sectional surveys will be conducted at 6 and 18 months post-net distribution; 70 individuals per cluster will be randomly selected for each cross-sectional survey, stratified by age (< 5 years, 5–9 years, 10–14 years, ≥15 years and older) from each of the 60 clusters. |
| **Unit of allocation** | Village was the unit of randomisation (60 clusters) |
| **Method of Randomization/ matching or other** | The protocol states that “Restricted randomisation will be used to allocate the clusters to arms in order to ensure that they are balanced on known factors that may affect the main outcomes. The following factors will be used: baseline (pre-intervention) malaria infection prevalence in children aged 0.5 to 10 years, cluster-level LLIN usage (prior to distribution), socio-economic status, village size, species composition (proportion An.gambiae/An.coluzzii) and distance to nearest health facility” |
| **Number of clusters per arm** | 20 clusters per arm (60 clusters total) |
| **Adjustment for clustering** | The statistical analysis plan provided by the authors states “…using a Cox proportional hazards model allowing for multiple events per child and using robust estimates of variance to account for the clustered design” and “…The unit of analysis will be the individual participant with clustering account for using random effects” |
| **Cluster details**  (including buffer sizes between clusters, other indication of dilution effects) | Each cluster will be comprised of 1 village or a group of villages for an average of 200 households (1200 residents) per cluster. Clusters will have a minimum of 100 children and 100 households.  A “fried egg” design will be used for cluster boundaries. Clusters will be designed with core and buffer areas to reduce the likelihood of spill-over of intervention effects. Nets will be distributed to all households in a cluster (i.e. core and buffer areas) but data collection to measure intervention effects will be restricted to households situated in the core areas to avoid spillover from neighbouring clusters receiving different nets. The cluster demarcation will be performed using the spatial analyst toolbox in ArcGIS (ESRI, Redlands, USA) based on the following criteria; a minimum of 50 children aged 6 months to 10 years in the core area and a buffer of minimum 1000 m between core households and any households in an adjacent cluster. |
| **Number of participants per arm** (including number of exclusions and reasons) | Each cluster will be comprised of 1 village or a group of villages for an average of 200 households (1200 residents) per cluster. Clusters will have a minimum of 100 children and 100 households.  Clusters would have a minimum of 100 children and 100 households to get a minimum of 50 children less than 10 years to be enrolled in the cohort.  A cohort of 30 children per cluster aged 6 months to 10 years old will be randomly selected from a census list of children living in the study clusters at the beginning of the first year and followed every two weeks/1 month over a 21 months period. |
| **Outcomes assessed** | Malaria Case Incidence Rate  Parasite Prevalence  Prevalence of Anaemia |
| **SETTING** | |
| **Country** | Benin |
| **Site/s**  (town/settlements/region) | Cove, Zagnanado, and Ouinhi Districts, located in the Zou department, central Benin, 154 km north of Cotonou. This area consists of 123 villages with approximately 54,000 households and population size of 220,000 inhabitants. The main economic activities of the population are farming, fishing, hunting, and trading. |
| **Peak transmission season** | April to November with two peaks during the rainy seasons from April to July and then from October to November |
| **Baseline malaria endemicity/ level of transmission** | A prevalence of malaria infection between 20 to 40%.  Malaria is highly endemic, and Malaria infection prevalence in the Zou department was 20% in children under 5 years old according to the demographic health survey (DHS) conducted in 2011 to 2012 and increased to 36.5% in the 2017–2018 DHS. |
| **Study site (e.g., rural/ urban/ peri-urban/ level of urbanicity)** | Rural |
| **Vector species and vector profile details**  (i.e., behaviours, resistance profile/ susceptibility tests, parity, sporozoite rates and all other reported information) | Main vector species were Anopheles coluzzii and Anopheles gambiae sensu stricto. High pyrethroid resistance in the main malaria vectors. Entomological surveys performed in the Cove region in 2015 revealed high levels of pyrethroid resistance intensity (> 200 fold) mainly due to high frequencies of kdr of > 90% and elevated cytochrome P450 enzymes. |
| **Malaria species** | P. falciparum |
| **PARTICIPANTS (those who received the intervention and on whom impact was measured)** | |
| **Characteristics, numbers, and demographics of participants who received the intervention** | "In the cohort we had 48% female children (aged 6 months to 10 years)."  "In the 6 month cross-sectional we had 45% female and in the 18 month cross-sectional we had 54% female. The cross sectionals were all ages"  - Direct correspondence with author |
| **Characteristics, numbers, and demographics of participants on whom the impact was measured.** | The cohort will include children aged 6 months to 9 years old resident in the study villages, without any severe illnesses and whose parents/carers have given written informed consent for their child to be included in the study. Inclusion criteria during cross-sectional surveys will be the willingness to participate and provide consent (or for parents/carers to provide consent for children); resident in the village during the previous 3 months, selected individuals without severe illness. |
| **Frequency of travel in the last month** | Not reported |
| **INTERVENTION 1** | |
| **Dual AI net brand, insecticide type, dose, material of net**  (including active ingredient, timing and frequency of application, durability of the net and insecticide) | Interceptor G2  Mixture LLIN made of polyester netting (100 deniers) coated with a wash-resistant formulation of 200 mg/m2 chlorfenapyr and 100 mg/m2 alpha-cypermethrin. The nets are blue, rectangular, and identical sizes (1.8m long, 1.6m wide, and 1.8m high) |
| **Net treatment strategy**  (e.g., how were the nets treated with the insecticide(s)) | Not reported |
| **Deployment strategy of nets**  (e.g., who received the nets? Where the houses in which they were installed permanent? What was the frequency of distribution?) | A short questionnaire will be used to collect demographic details on household residents to estimate the number of nets required to be distributed in each house and to randomly select children for the cohort. Each building will be mapped using a Global Positioning System to assist with delineating clusters.  Nets will be distributed with the support of the National Malaria Control Programme (NMCP). All households in the study area will receive one net for every two people. The census listing will be used to facilitate net distribution at a central location in each hamlet.  One LLIN was given to every two people as recommended by NMCP. The study LLIN requirement has been calculated following standard guideline to cover 100% of the study area population (population divided by 1.6 to account for households with uneven numbers of members). The household listing, generated during the house mapping and registration, will be used to distribute the LLIN at a central location in each hamlet. Householders will be asked to return their old net and use the new LLIN provided. |
| **How was the intervention measured?**  (e.g. how was net usage monitored/observed by the authors? How was coverage of nets measured?) | The coverage achieved in each cluster will be evaluated through a post-intervention coverage survey one month after distribution. Net coverage and usage will also be assessed during cohort visits and cross-sectional surveys. Three indicators will be used: proportion of households with at least one ITN for every 2 people”, “proportion of household members with enough ITNs to sleep under (population access)” and “proportion of residents reporting using an ITN last night |
| **Coverage of household / person**  (% of households with at least 1 ITN  % of households with at least 1 ITN for every 2 people  % of population with access to an ITN  Nets per HH or per person) | One LLIN was given to every two people as recommended by NMCP (1 to 2 people =1 LLIN, 3/4 people=2 LLINs, 5/6= 3 LLINs etc...). The study LLIN requirement has been calculated following standard guideline to cover 100% of the study area population (population divided by 1.6 to account for households with uneven numbers of members). The household listing, generated during the house mapping and registration, will be used to distribute the LLIN at a central location in each hamlet. Householders will be asked to return their old net and use the new LLIN provided. |
| **Coverage across cluster/site/jurisdiction** | Not reported (however protocol suggests aiming of 85% access with 75% usage). |
| **Length of intervention and time points of outcome measurement** | Outcomes measured at 6, 18 and 24 months. Intervention was maintained throughout |
| **Changes in human behaviour** (e.g., sleeping behaviour, non-intervention-based spraying of houses) | No other interventions were conducted in the area (including IRS) - Direct correspondence with author |
| **Background interventions**  (all reported in primary study – e.g., spraying prior to treatment period (and during) including coverage of background interventions. Other malaria or vector-specific control interventions [indoor surface treatment, nets/ other insecticides/ barrier], cointerventions, treatment of individuals that may impact outcome) | To reach a minimum of 85% access following the distribution and a minimum of 75% usage, information, education, and communication (IEC) activities will be conducted before, during, and after LLIN distribution to increase usage in the study area, including instructions on when and how to wash the nets. A door to door hang-up campaign will take place after the distribution and depending on net usage rates, subsequent hang-up campaigns will be organized to increase net usage. Throughout the study, community health workers will be utilized to encourage continuous net use in the study population. Hamlet and religious leaders will be also involved in the sensitization  campaigns for net usage. |
| **INTERVENTION 2** | |
| **Dual AI net brand, insecticide type, dose, material of net**  (including active ingredient, timing and frequency of application, durability of the net and insecticide) | Royal Guard  Mixture LLIN made of polyethylene (120 deniers) incorporating 225 mg/m2 pyriproxyfen and 261 mg/m2 alpha-cypermethrin. The nets are blue, rectangular, and identical sizes (1.8m long, 1.6m wide, and 1.8m high). |
| **Net treatment strategy**  (e.g., how were the nets treated with the insecticide(s)) | Not reported |
| **Deployment strategy of nets**  (e.g., who received the nets? Where the houses in which they were installed permanent? What was the frequency of distribution?) | Same as above (Intervention 1) |
| **How was the intervention measured?**  (e.g. how was net usage monitored/observed by the authors? How was coverage of nets measured?) | Same as above (Intervention 1) |
| **Coverage of household / person**  (% of households with at least 1 ITN  % of households with at least 1 ITN for every 2 people  % of population with access to an ITN  Nets per HH or per person) | Same as above (Intervention 1) |
| **Coverage across cluster/site/jurisdiction** | Not reported (however protocol suggests aiming of 85% access with 75% usage). |
| **Length of intervention and time points of outcome measurement** | Outcomes measured at 6, 18 and 24 months. Intervention was maintained throughout |
| **Changes in human behaviour** (e.g., sleeping behaviour, non-intervention-based spraying of houses) | Not reported |
| **Background interventions**  (all reported in primary study – e.g., spraying prior to treatment period (and during) including coverage of background interventions. Other malaria or vector-specific control interventions [indoor surface treatment, nets/ other insecticides/ barrier], cointerventions, treatment of individuals that may impact outcome) | Same as above (Intervention 1) |
| **COMPARISON** | |
| **Dual AI net brand, insecticide type, dose, material of net**  (including active ingredient, timing and frequency of application, durability of the net and insecticide) | Interceptor LLIN  A pyrethroid-treated LLIN with alpha-cypermethrin (coated  onto filaments) at a target dose of 200 mg/m2 of polyester  fabric (100 deniers). The nets are blue, rectangular, and identical sizes (1.8m long, 1.6m wide, and 1.8m high). |
| **Net treatment strategy**  (e.g., how were the nets treated with the insecticide(s)) | Not reported |
| **Deployment strategy of nets**  (e.g., who received the nets? Where the houses in which they were installed permanent? What was the frequency of distribution?) | Same as above (Intervention 1) |
| **How was the intervention measured?**  (e.g. how was net usage monitored/observed by the authors? How was coverage of nets measured?) | Same as above (Intervention 1) |
| **Coverage of household / person**  (% of households with at least 1 ITN  % of households with at least 1 ITN for every 2 people  % of population with access to an ITN  Nets per HH or per person) | Same as above (Intervention 1) |
| **Coverage across cluster/site/jurisdiction** | Not reported (however protocol suggests aiming of 85% access with 75% usage). |
| **Length of intervention and time points of outcome measurement** | Outcomes measured at 6, 18 and 24 months. Intervention was maintained throughout |
| **Changes in human behaviour** (e.g., sleeping behaviour, non-intervention-based spraying of houses) | Not reported |
| **Any other details regarding comparator not described elsewhere.** | Same as above (Intervention 1) |
| **MALARIA CASE INCIDENCE RATE**  Defined as symptoms plus parasitaemia, over a population at risk or person-time. Detected either through passive or active surveillance. | |
| **Name** | As above |
| **Definition/ assessment metric** | The primary outcome is malaria case incidence in children aged 6 months to 10 years over 24 months. A malaria case is defined as an infra-rouge frontal temperature above 37.5 °C or history of a fever in the last 48 h and a positive rapid diagnostic test (RDT).  Malaria case incidence was measured in an active cohort of approximately 30 children per cluster followed up for 20 months (for 2 years following the net distribution- follow up was delayed immediately following the net distribution due to the Covid pandemic). |
| **Events** | Data presented as no. clinical episodes (follow-up time child-years)  OVERALL  Pyrethroid-only LLIN Group = 898 (874.4)  Pyriproxyfen-pyrethroid Group = 743 (883.9)  Chlorfenapyr-pyrethroid Group = 494 (887.3)  YEAR 1  Pyrethroid-only LLIN Group = 268 (344.5)  Pyriproxyfen-pyrethroid Group = 211 (342.7)  Chlorfenapyr-pyrethroid Group = 124 (349.2)  YEAR 2  Pyrethroid-only LLIN Group = 630 (529.9)  Pyriproxyfen-pyrethroid Group = 532 (541.2)  Chlorfenapyr-pyrethroid Group = 370 (538.1) |
| **Total (or unit time)** | Unit time presented above |
| **Time of outcome assessment** | Year 1 and 2 presented above as is combined data |
| **Results**  (i.e., main results – epidemiological, unadjusted and adjusted, secondary outcomes, sensitivity analyses, subgroups, and clusters) | Results presented as incidence per child-year (95% CI for each group)  OVERALL  Pyrethroid-only LLIN Group = 1.02 (0.96 – 1.09)  Pyriproxyfen-pyrethroid Group = 0.84 (0.78 – 0.90)  Chlorfenapyr-pyrethroid Group = 0.56 (0.51 – 0.61)  YEAR 1  Pyrethroid-only LLIN Group = 0.77 (0.69 – 0.87)  Pyriproxyfen-pyrethroid Group = 0.61 (0.54 – 0.7)  Chlorfenapyr-pyrethroid Group = 0.35 (0.29 – 0.42)  YEAR 2  Pyrethroid-only LLIN Group = 1.19 (1.09 – 1.28)  Pyriproxyfen-pyrethroid Group = 0.98 (0.9 – 1.07)  Chlorfenapyr-pyrethroid Group = 0.69 (0.62 – 0.76) |
| **Effect type** | IRR |
| **MALARIA INFECTION INCIDENCE**  Defined as parasitaemia with or without symptoms, over a population at risk or person-time. Detected through passive or active surveillance. | |
| **Name** | N/A |
| **Definition/ assessment metric** | N/A |
| **Events** | N/A |
| **Total (or unit time)** | N/A |
| **Time of outcome assessment** | N/A |
| **Results**  (i.e., main results – epidemiological, unadjusted and adjusted, secondary outcomes, sensitivity analyses, subgroups, and clusters) | N/A |
| **Effect type** | N/A |
| **INCIDENCE OF SEVERE DISEASE**  Defined as hospitalization with parasitaemia, over a population at risk or person-time. | |
| **Name** | N/A |
| **Definition/ assessment metric** | N/A |
| **Events** | N/A |
| **Total (or unit time)** | N/A |
| **Time of outcome assessment** | N/A |
| **Results**  (i.e., main results – epidemiological, unadjusted and adjusted, secondary outcomes, sensitivity analyses, subgroups, and clusters) | N/A |
| **Effect type** | N/A |
| **PARASITE PREVALENCE**  Parasitaemia with or without symptoms, over the population sampled. Detected through cross-sectional surveys. | |
| **Name** | As above |
| **Definition/ assessment metric** | Malaria infection prevalence in the study population at 6 and 18 months post bed net distribution.  Prevalence was measured in cross-sectional surveys in a random sample of approximately 70 people per cluster, of any age. Infection was measured using RDT and people were tested regardless of symptoms. Cross-sectional surveys took place 6 and 18 months after net distribution. |
| **Events** | Results presented as n/N (%)  6 MONTHS POST-INTERVENTION  Pyrethroid-only LLIN Group = 412/1471 (28%)  Pyriproxyfen-pyrethroid Group = 394/1463 (26.9%)  Chlorfenapyr-pyrethroid Group = 231/1475 (15.7%)  18 MONTHS POST-INTERVENTION  Pyrethroid-only LLIN Group = 576/1489 (38.7%)  Pyriproxyfen-pyrethroid Group = 564/1468 (38.2%)  Chlorfenapyr-pyrethroid Group = 414/1483 (27.9%) |
| **Total (or unit time)** | As above |
| **Time of outcome assessment** | 6 and 18 months post-intervention |
| **Results**  (i.e., main results – epidemiological, unadjusted and adjusted, secondary outcomes, sensitivity analyses, subgroups, and clusters) | Results presented in comparison to the pyrethroid-only group (reference) as OR (95% CI) and p-value  6 MONTHS POST-INTERVENTION  Pyriproxyfen-pyrethroid Group = 0.92 (0.63-1.35) p = 0.6742  Chlorfenapyr-pyrethroid Group = 0.47 (0.32-0.69) p = 0.0002  18 MONTHS POST-INTERVENTION  Pyriproxyfen-pyrethroid Group = 0.97 (0.69-1.37) p = 0.8724  Chlorfenapyr-pyrethroid Group = 0.61 (0.43-0.85) p =0.0041 |
| **Effect type** | OR |
| **ALL-CAUSE MORTALITY**  Number of deaths over the population at risk or person-time. | |
| **Name** | N/A |
| **Definition/ assessment metric** | N/A |
| **Events** | N/A |
| **Total (or unit time)** | N/A |
| **Time of outcome assessment** | N/A |
| **Results**  (i.e., main results – epidemiological, unadjusted and adjusted, secondary outcomes, sensitivity analyses, subgroups, and clusters) | N/A |
| **Effect type** | N/A |
| **MALARIA MORTALITY**  Number of deaths attributed to malaria over the population at risk or person-time. | |
| **Name** | N/A |
| **Definition/ assessment metric** | N/A |
| **Events** | N/A |
| **Total (or unit time)** | N/A |
| **Time of outcome assessment** | N/A |
| **Results**  (i.e., main results – epidemiological, unadjusted and adjusted, secondary outcomes, sensitivity analyses, subgroups, and clusters) | N/A |
| **Effect type** | N/A |
| **PREVALENCE OF ANAEMIA**  Defined by study thresholds of anaemia. | |
| **Name** | As above |
| **Definition/ assessment metric** | Anaemia was measured in children aged under 5 years in the cross-sectional surveys at 6 and 18 months post-net distribution. Hemoglobin (measured by Haemocue), defined as < 10 g/dL (moderate) and < 8 g/ dL (severe). |
| **Events** | Results presented as n/N (%)  6 MONTHS POST-INTERVENTION  Pyrethroid-only LLIN Group = 99/241 (41.1%)  Pyriproxyfen-pyrethroid Group = 117/250 (46.8%)  Chlorfenapyr-pyrethroid Group = 82/241 (34%)  18 MONTHS POST-INTERVENTION  Pyrethroid-only LLIN Group = 118/252 (46.8%)  Pyriproxyfen-pyrethroid Group = 108/245 (44.1%)  Chlorfenapyr-pyrethroid Group = 118/246 (47.9%) |
| **Total (or unit time)** | As above |
| **Time of outcome assessment** | 6 and 18 months post-intervention |
| **Results**  (i.e., main results – epidemiological, unadjusted and adjusted, secondary outcomes, sensitivity analyses, subgroups, and clusters) | Results presented in comparison to the pyrethroid-only group (reference) as OR (95% CI) and p-value  6 MONTHS POST-INTERVENTION  Pyriproxyfen-pyrethroid Group = 1.24 (0.71-2.18) p = 0.4452  Chlorfenapyr-pyrethroid Group = 0.71 (0.4-1.27) p = 0.2447  18 MONTHS POST-INTERVENTION  Pyriproxyfen-pyrethroid Group = 0.84 (0.39-1.79) p = 0.6545  Chlorfenapyr-pyrethroid Group = 1.08 (0.51-2.228) p = 0.8429 |
| **Effect type** | OR |
| **COSTS** | |
| **Event/raw costs** | Not reported |
| **Results** | Not reported |
| **Time of cost assessment** | Not reported |
| **Resources needed/ used** | Not reported |
| **ADDITIONAL DATA** | |
| **Unintended benefits** | Not reported |
| **Harms** | Collected according to protocol but not reported |
| **Other contextual information present/measured/reported)** (feasibility, acceptability, preferences/values, impact on equity) | Not reported |
| **Entomological outcomes measured** (list) | EIR, as a measure for malaria transmission rate in the primary vector species. Mosquito density and survivorship, mosquito resting behaviour, species composition, ovary development, and fecundity.  Frequency and intensity of phenotypic and genotypic resistance to pyrethroid, chlorfenapyr, and pyriproxyfen insecticides.  Prevalence of elevated cytochrome P450s and other metabolic enzymes associated with pyrethroid resistance. |
| **Other** | Not reported |
| **Source of funding** | This research is supported by a grant to the London School of Hygiene and Tropical Medicine from UNITAID and Global Fund via the Innovative Vector Control Consortium (IVCC). This cluster-randomized clinical trial is part of a larger project “The New Net project”. The funders do not play a role in study design, collection, management, analysis and interpretation of data. They do not influence the writing of the report and the decision to submit. |
| **Possible conflicts of interest** | The authors declare that they have no competing interests. |

| **Domain** | **Signalling question** | **Response** | **Comments** |
| --- | --- | --- | --- |
| **Bias arising from the randomization process** | 1a.1 Was the allocation sequence random? | Y | The protocol states that “Restricted randomisation will be used to allocate the clusters to arms in order to ensure that they are balanced on known factors that may affect the main outcomes. The following factors will be used: baseline (pre-intervention) malaria infection prevalence in children aged 0.5 to 10 years, cluster-level LLIN usage (prior to distribution), socio-economic status, village size, species composition (proportion An.gambiae/An.coluzzii) and distance to nearest health facility”  Unclear if the sequence was concealed. |
|  | 1a.2 Was the allocation sequence concealed until clusters were enrolled and assigned to interventions? | NI |  |
|  | 1a.3 Did baseline differences between intervention groups suggest a problem with the randomization process? | N | No baseline demographics were provided in the requested information from the authors. However, restricted randomisation based on baseline variables should account for this. |
|  | **Risk of bias judgement** | **Some concerns** | The protocol states that “Restricted randomisation will be used to allocate the clusters to arms in order to ensure that they are balanced on known factors that may affect the main outcomes. The following factors will be used: baseline (pre-intervention) malaria infection prevalence in children aged 0.5 to 10 years, cluster-level LLIN usage (prior to distribution), socio-economic status, village size, species composition (proportion An.gambiae/An.coluzzii) and distance to nearest health facility”  Unclear if the sequence was concealed. No baseline demographics were provided in the requested information from the authors. However, restricted randomisation based on baseline variables should account for this. |
| **Bias arising from the timing of identification or recruitment of participants** | 1b.1 Were all the individual participants identified and recruited (if appropriate) before randomization of clusters? | **Y** |  |
|  | 1b.2 If N/PN/NI to 1b.1: Is it likely that selection of individual participants was affected by knowledge of the intervention assigned to the cluster? |  |  |
|  | 1b.3 Were there baseline imbalances that suggest differential identification or recruitment of individual participants between intervention groups? | **N** | No baseline demographics were provided in the requested information from the authors. However, restricted randomisation based on baseline variables should account for this. |
|  | **Risk of bias judgement** | **Low** | Low |
| **Bias due to deviations from intended interventions** | 2.1a Were participants aware that they were in a trial? | Y | All participants had to provide informed consent, or informed consent was provided by parents/caregivers of young children |
|  | 2.1b If Y/PY/NI to 2.1a: Were participants aware of their assigned intervention during the trial? | N | Authors describe this as a single blinded study, but then state "Study participants will be blinded to the type of nets they have received. All field staff will be blinded to the allocation and analyses will be conducted on blinded data".  This suggests complete blinding |
|  | 2.2 Were carers and people delivering the interventions aware of participants' assigned intervention during the trial? | N |  |
|  | 2.3 If Y/PY/NI to 2.1b or 2.2: Were there deviations from the intended intervention that arose because of the trial context? | NA |  |
|  | 2.4 If Y/PY to 2.3: Were these deviations likely to have affected the outcome? | NA |  |
|  | 2.5 If Y/PY/NI to 2.4: Were these deviations from intended intervention balanced between groups? | NA |  |
|  | 2.6 Was an appropriate analysis used to estimate the effect of assignment to intervention? | Y | ITT and per protocol analyses were used. |
|  | 2.7 If N/PN/NI to 2.6: Was there potential for a substantial impact (on the result) of the failure to analyse participants in the group to which they were randomized ? | NA |  |
|  | **Risk of bias judgement** | **Low** | All participants had to provide informed consent, or informed consent was provided by parents/caregivers of young children  Authors describe this as a single blinded study, stating "Study participants will be blinded to the type of nets they have received. All field staff will be blinded to the allocation and analyses will be conducted on blinded data".        ITT and per protocol analyses were used. |
| **Outcome: Malaria Case Incidence** | | | |
| **Bias due to missing outcome data** | 3.1a Were data for this outcome available for all clusters that recruited participants? | Y | All clusters were included in the analyses. |
|  | 3.1b Were data for this outcome available for all, or nearly all, participants within clusters? | N | Malaria case incidence was measured in an active cohort of approximately 30 children per cluster followed up for 20 months. These 30 children were chosen randomly. |
|  | 3.2 If N/PN/NI to 3.1a or 3.1b: Is there evidence that the result was not biased by missing data? | Y | Random selection suggests that the result will not be biased |
|  | 3.3 If N/PN to 3.2 Could missingness in the outcome depend on its true value? | NA |  |
|  | 3.4 If Y/PY/NI to 3.3: Is it likely that missingness in the outcome depended on its true value? | NA |  |
|  | **Risk of bias judgement** | **Low** | All clusters were included in the analyses.   Malaria case incidence was measured in an active cohort of approximately 30 children per cluster followed up for 20 months. These 30 children were chosen randomly.  Random selection suggests that the result will not be biased |
| **Bias in measurement of the outcome** | 4.1 Was the method of measuring the outcome inappropriate? | N | Malaria was confirmed if a temperature was recorded and a positive RDT was returned. |
|  | 4.2 Could measurement or ascertainment of the outcome have differed between intervention groups? | N |  |
|  | 4.3a If N/PN/NI to 4.1 and 4.2: Were outcome assessors aware that a trial was taking place? | Y |  |
|  | 4.3b If Y/PY/NI to 4.3a: Were outcome assessors aware of the intervention received by study participants? | N | Authors describe this as a single blinded study, but then state "Study participants will be blinded to the type of nets they have received. All field staff will be blinded to the allocation and analyses will be conducted on blinded data".  This suggests complete blinding |
|  | 4.4 If Y/PY/NI to 4.3b: Could assessment of the outcome have been influenced by knowledge of intervention received? | NA |  |
|  | 4.5 If Y/PY/NI to 4.4: Is it likely that assessment of the outcome was influenced by knowledge of intervention received? | NA |  |
|  | **Risk of bias judgement** | **Low** | Malaria was confirmed if a temperature was recorded and a positive RDT was returned.      Authors describe this as a single blinded study, but then state "Study participants will be blinded to the type of nets they have received. All field staff will be blinded to the allocation and analyses will be conducted on blinded data".  This suggests complete blinding |
| **Bias in selection of the reported result** | 5.1 Were the data that produced this result analysed in accordance with a pre-specified analysis plan that was finalized before unblinded outcome data were available for analysis? | Y | The authors have provided a pre-specified analysis plan, and protocol. |
|  | 5.2 ... multiple eligible outcome measurements (e.g. scales, definitions, time points) within the outcome domain? | N | Not all data available yet. |
|  | 5.3 ... multiple eligible analyses of the data? | N | Not all data available yet. |
|  | **Risk of bias judgement** | **Low** | The authors have provided a pre-specified analysis plan, and protocol. Not all data available yet.  Not all data available yet. |
| **Outcome: Parasite Prevalence** | | | |
| **Bias due to missing outcome data** | 3.1a Were data for this outcome available for all clusters that recruited participants? | Y | All clusters were included in the analyses. |
|  | 3.1b Were data for this outcome available for all, or nearly all, participants within clusters? | N | Prevalence was measured in cross-sectional surveys in a random sample of approximately 70 people per cluster, of any age. |
|  | 3.2 If N/PN/NI to 3.1a or 3.1b: Is there evidence that the result was not biased by missing data? | Y | Random selection suggests that the result will not be biased |
|  | 3.3 If N/PN to 3.2 Could missingness in the outcome depend on its true value? | NA |  |
|  | 3.4 If Y/PY/NI to 3.3: Is it likely that missingness in the outcome depended on its true value? | NA |  |
|  | **Risk of bias judgement** | **Low** | All clusters were included in the analyses.  Prevalence was measured in cross-sectional surveys in a random sample of approximately 70 people per cluster, of any age. Random selection suggests that the result will not be biased |
| **Bias in measurement of the outcome** | 4.1 Was the method of measuring the outcome inappropriate? | N | Infection was measured using RDT and people were tested regardless of symptoms. |
|  | 4.2 Could measurement or ascertainment of the outcome have differed between intervention groups? | N |  |
|  | 4.3a If N/PN/NI to 4.1 and 4.2: Were outcome assessors aware that a trial was taking place? | Y |  |
|  | 4.3b If Y/PY/NI to 4.3a: Were outcome assessors aware of the intervention received by study participants? | N | Authors describe this as a single blinded study, but then state "Study participants will be blinded to the type of nets they have received. All field staff will be blinded to the allocation and analyses will be conducted on blinded data".  This suggests complete blinding |
|  | 4.4 If Y/PY/NI to 4.3b: Could assessment of the outcome have been influenced by knowledge of intervention received? | NA |  |
|  | 4.5 If Y/PY/NI to 4.4: Is it likely that assessment of the outcome was influenced by knowledge of intervention received? | NA |  |
|  | **Risk of bias judgement** | **Low** | Infection was measured using RDT and people were tested regardless of symptoms.       Authors describe this as a single blinded study, but then state "Study participants will be blinded to the type of nets they have received. All field staff will be blinded to the allocation and analyses will be conducted on blinded data".  This suggests complete blinding |
| **Bias in selection of the reported result** | 5.1 Were the data that produced this result analysed in accordance with a pre-specified analysis plan that was finalized before unblinded outcome data were available for analysis? | Y | The authors have provided a pre-specified analysis plan, and protocol. |
|  | 5.2 ... multiple eligible outcome measurements (e.g. scales, definitions, time points) within the outcome domain? | N | Not all data available yet. |
|  | 5.3 ... multiple eligible analyses of the data? | N | Not all data available yet. |
|  | **Risk of bias judgement** | **The authors have provided a pre-specified analysis plan, and protocol.  Not all data available yet.   Not all data available yet.** |  |
| **Outcome: Prevalence of Anaemia** | | | |
| **Bias due to missing outcome data** | 3.1a Were data for this outcome available for all clusters that recruited participants? | Y | All clusters were included in the analyses. |
|  | 3.1b Were data for this outcome available for all, or nearly all, participants within clusters? | N | Prevalence was measured in cross-sectional surveys in a random sample of approximately 70 people per cluster, of any age. |
|  | 3.2 If N/PN/NI to 3.1a or 3.1b: Is there evidence that the result was not biased by missing data? | Y | Random selection suggests that the result will not be biased |
|  | 3.3 If N/PN to 3.2 Could missingness in the outcome depend on its true value? | NA |  |
|  | 3.4 If Y/PY/NI to 3.3: Is it likely that missingness in the outcome depended on its true value? | NA |  |
|  | **Risk of bias judgement** | **Low** | All clusters were included in the analyses.   Prevalence was measured in cross-sectional surveys in a random sample of approximately 70 people per cluster, of any age.   Random selection suggests that the result will not be biased |
| **Bias in measurement of the outcome** | 4.1 Was the method of measuring the outcome inappropriate? | N | Hemoglobin concentration (measured by Haemocue), defined as < 10 g/dL (moderate) and < 8 g/ dL (severe). |
|  | 4.2 Could measurement or ascertainment of the outcome have differed between intervention groups? | N |  |
|  | 4.3a If N/PN/NI to 4.1 and 4.2: Were outcome assessors aware that a trial was taking place? | Y |  |
|  | 4.3b If Y/PY/NI to 4.3a: Were outcome assessors aware of the intervention received by study participants? | N | Authors describe this as a single blinded study, but then state "Study participants will be blinded to the type of nets they have received. All field staff will be blinded to the allocation and analyses will be conducted on blinded data".  This suggests complete blinding |
|  | 4.4 If Y/PY/NI to 4.3b: Could assessment of the outcome have been influenced by knowledge of intervention received? | NA |  |
|  | 4.5 If Y/PY/NI to 4.4: Is it likely that assessment of the outcome was influenced by knowledge of intervention received? | NA |  |
|  | **Risk of bias judgement** | **Low** | Hemoglobin concentration (measured by Haemocue), defined as < 10 g/dL (moderate) and < 8 g/ dL (severe).      Authors describe this as a single blinded study, but then state "Study participants will be blinded to the type of nets they have received. All field staff will be blinded to the allocation and analyses will be conducted on blinded data".  This suggests complete blinding |
| **Bias in selection of the reported result** | 5.1 Were the data that produced this result analysed in accordance with a pre-specified analysis plan that was finalized before unblinded outcome data were available for analysis? | Y | The authors have provided a pre-specified analysis plan, and protocol. |
|  | 5.2 ... multiple eligible outcome measurements (e.g. scales, definitions, time points) within the outcome domain? | N | Not all data available yet. |
|  | 5.3 ... multiple eligible analyses of the data? | N | Not all data available yet. |
|  | **Risk of bias judgement** | **The authors have provided a pre-specified analysis plan, and protocol.  Not all data available yet.   Not all data available yet.** |  |

## Mosha 2022

| **Study: Effectiveness and cost-effectiveness against malaria of three types of dual-active-ingredient long-lasting insecticidal nets (LLINs) compared with pyrethroid-only LLINs in Tanzania: a four-arm, cluster-randomised trial** | |
| --- | --- |
| **DESIGN** | |
| **Lead Author** | Jacklin F Mosha |
| **Publication year** | 2022 |
| **Trial name** | Not reported |
| **Other reports of this study**  (i.e., author, year, title, doi) | Mosha 2021 (Protocol) |
| **Time period study was conducted** | Selection of villages and households was done during the census between May 11 and July 2, 2018. Enrolment of children for the cohort was done in March, 2019. Between Jan 26 and 28, 2019, LLINs were distributed among households in the study area. |
| **Design** | Four parallel-arm, superiority, cluster-randomised, controlled trial |
| **Eligibility criteria of participants** | No specific eligibility criteria of participants were mentioned. All individuals in the study area were considered eligible if they provided informed consent (or an adult provided informed consent in the case of children) to receiving and using a LLIN.  Inclusion criteria for prevalence and incidence data collection were households with at least one child of appropriate age who permanently resided in the selected household and an adult caregiver who could provide written consent. |
| **Recruitment methods and rate** | Five potential study sites in Tanzania’s Victoria lake zone were evaluated based on four criteria: report of a minimum of 30% malaria infection prevalence in total population, A. gambiae sensu stricto (s.s.) or A. funestus s.s. as the main vectors, insecticide pyrethroid resistance in standard WHO bioassays (<50% mortality), and no indoor residual spraying (IRS) planned for the next 3 years.  Selection of villages and households was done during the census between May 11 and July 2, 2018. Enrolment of children for the cohort was done in March, 2019. The study area included 39 307 households across 72 villages. These households formed 84 clusters, which were evenly distributed between the four groups (21 [25%] clusters- per group). Between Jan 26 and 28, 2019, a total of 147 230 LLINs were distributed among households in the study area.  28 599 core-area households were eligible for cross-sectional surveys of malaria prevalence. |
| **Unit of allocation** | Cluster (which consisted of at least 119 household) n = 84  A cluster was defined in the protocol as a village or group of hamlets with a minimum of 150 households containing children aged 6 months to 14 years living in the core area; this number was later reduced to 119 households during mapping and census. |
| **Method of Randomization/ matching or other** | An independent statistician conducted constrained randomisation to allocate the 84 clusters to the four study groups at a ratio of 1:1:1:1, ensuring that absolute differences in cluster means between study groups were within the specified ranges for specific cluster characteristics.  Randomisations was verified by checking the frequency of allocations to the same study group of all pairs of clusters.  The inhabitants of each cluster and the field staff who were responsible for enrolment and collected data were masked to the type of LLIN allocated. LLINs of each type were similar in appearance apart from a colour-coded loop and a unique identifying code. |
| **Number of clusters per arm** | Clusters were allocated to one of four study groups: the pyrethroid-only (reference) group; the pyriproxyfen group; the chlorfenapyr group; and the piperonyl butoxide group. There were 86 clusters in total and 21 clusters in each arm. |
| **Adjustment for clustering** | The study has adjusted for baseline cluster-level variables used in the randomisation procedure.  The within-period intracluster correlation coefficient (ICC) was 0·059, with a between-period ICC of 0·022 and cluster autocorrelation coefficient (CAC) of 0·375. |
| **Cluster details**  (including buffer sizes between clusters, other indication of dilution effects) | A mapping census was performed to determine clusters. A cluster was defined in the protocol as a village or group of hamlets with a minimum of 150 households containing children aged 6 months to 14 years living in the core area. Clusters were designed with core and buffer areas, with all houses in core areas located at least 600 m from any houses in adjacent clusters to reduce contamination of intervention.  The study area included 39307 households across 72 villages. These households formed 84 clusters, which were evenly distributed between the four groups (21 [25%] clusters per group). |
| **Number of participants per arm** (including number of exclusions and reasons) | Pyrethroid-only group (61183) Pyriproxyfen group (57567) Chlorfenapyr group (60115) PBO group (57631)  Numbers lost to follow up varied but remained balanced between groups, and is reported below with the outcome data. Common reasons for loss to follow up include: children not attending clinical appointment, malaria test result missing and death. Lost to follow up was not presented available for the prevalence data. |
| **Outcomes assessed** | Parasite prevalence (defined in the study as malaria prevalence) Malaria case incidence All-cause mortality Malaria mortality Prevalence of anaemia |
| **SETTING** | |
| **Country** | Northwest Tanzania |
| **Site/s**  (town/settlements/region) | Misungwi district of Mwanza |
| **Peak transmission season** | The dry season is July to August.  High transmission season is October to July.  Low transmission season is August to September. |
| **Baseline malaria endemicity/ level of transmission** | Misungwi has moderate to high malaria transmission. In a study conducted in 2010, prevalence was 52% across all age groups. During the preliminary assessment in May 2018, presence of all main Tanzanian malaria vectors, A. gambiae s.s., A. arabiensis and A. funestus s.s., were found in the area.  Baseline mean household EIR  Pyrethroid-only group = 0·35 (0·72), N=331  Pyriproxyfen group = 0·11 (0·34), N=328  Chlorfenapyr group = 0·04 (0·20), N=332  Piperonyl butoxide group = 0·07 (0·26), N=326 |
| **Study site (e.g., rural/ urban/ peri-urban/ level of urbanicity)** | Rural to Urban (mixed) |
| **Vector species and vector profile details**  (i.e., behaviours, resistance profile/ susceptibility tests, parity, sporozoite rates and all other reported information) | 94.5% of the species identified were A funestus. The protocol mentions that A. gambiae s.s., A. arabiensis have also been detected in the area. Pyrethroid resistance was high in A funestus, with mortality below 85% after exposure to 10 times diagnostic concentrations of a-cypermethrin or permethrin. Exposure to pyriproxyfen sterilised only 127 (24%) of 536 of A funestus and 23 (21%) of 112 A gambiae sensu lato. No resistance was observed against chlorfenapyr insecticide |
| **Malaria species** | Plasmodium falciparum |
| **PARTICIPANTS (those who received the intervention and on whom impact was measured)** | |
| **Characteristics, numbers, and demographics of participants who received the intervention** | The numbers of the participants that received the intervention were not provided. However the characteristics of these participants has been provided at a cluster level.  PYRETHROID-ONLY GROUP STUDY CLUSTERS Overall study area: 61 183 Core cluster areas: 43 877 Mean number of people per household in the study area: 7.3 (3.3) HH AND CHILDREN (6 months - 14 years) Households: 680 Children: 1295 Median age of selected children, years: 6 (3-10), N=1295 Low socioeconomic status: 210/680 (30.9%) LLIN use in household residents of all age groups: 2957/4962 (59.6%) LLIN use in selected children: 831/1295 (64.2%) Malaria infection prevalence in selected children: 519/1130 (45.9%) Anaemia  prevalence in children: 28/453 (6.2%) CHILDREN ENROLLED IN COHORT (6 months - 10 years)(March 2019 and February 2020) Median age, years: 5 (3-7), N=1523 Female: 787/1523 (51.7%) Male: 736/1523 (48.3%) LLIN use are enrolment: 1450/1523 (95.2%)  PYRIPROXYFEN GROUP STUDY CLUSTERS Overall study area: 57 567 Core cluster areas: 43 266 Mean number of people per household in the study area: 6.8 (3.0) HH AND CHILDREN (6 months - 14 years) Households: 667 Children: 1290 Median age of selected children, years: 6 (3-10), N=1290 Low socioeconomic status: 230/667 (34.5%) LLIN use in household residents of all age groups: 2813/4520 (62.2%) LLIN use in selected children: 839/1290 (65.0%) Malaria infection prevalence in selected children: 516/1118 (46.2%) Anaemiaâ€  prevalence in children: 28/500 (5.6%) CHILDREN ENROLLED IN COHORT (6 months - 10 years)(March 2019 and February 2020) Median age, years: 5 (3-8), N=1523 Female: 789/1523 (51.8%) Male: 734/1523 (48.2%) LLIN use are enrolment: 1473/1523 (96.7%)  CHLORFENAPYR GROUP STUDY CLUSTERS Overall study area: 60 115 Core cluster areas: 41 748 Mean number of people per household in the study area: 7.2 (3.1) HH AND CHILDREN (6 months - 14 years) Households: 671 Children: 1249 Median age of selected children, years: 6 (3-9), N=1249 Low socioeconomic status: 209/671 (31.1%) LLIN use in household residents of all age groups: 2849/4803 (59.3%) LLIN use in selected children: 781/1249 (62.5%) Malaria infection prevalence in selected children: 469/1099 (42.7%) Anaemiaâ€  prevalence in children: 28/507 (5.5%) CHILDREN ENROLLED IN COHORT (6 months - 10 years)(March 2019 and February 2020) Median age, years: 5 (2-7), N=1495 Female: 718/1495 (48.0%) Male: 777/1495 (52.0%) LLIN use are enrolment: 1432/1495 (95.8%)  PBO GROUP STUDY CLUSTERS Overall study area: 57 631 Core cluster areas: 45 020 Mean number of people per household in the study area: 6.9 (2.8) HH AND CHILDREN (6 months - 14 years) Households: 638 Children: 1185 Median age of selected children, years: 6 (3-10), N=1185 Low socioeconomic status: 237/638 (37.1%) LLIN use in household residents of all age groups: 2695/4369 (61.7%) LLIN use in selected children: 751/1185 (63.4%) Malaria infection prevalence in selected children: 444/1056 (42.0%) Anaemiaâ€  prevalence in children: 20/472 (4.2%) CHILDREN ENROLLED IN COHORT (6 months - 10 years)(March 2019 and February 2020) Median age, years: 5 (3-8), N=1527 Female: 825/1527 (54.0%) Male: 702/1527 (46.0%) LLIN use are enrolment: 1467/1527 (96.1%) |
| **Characteristics, numbers, and demographics of participants on whom the impact was measured.** | Criteria for prevalence and incidence data collection were households with at least one child of appropriate age who permanently resided in the selected household and an adult caregiver who could provide written consent. For Prevalence data, children were eligible if aged between 6 months and 14 years. For incidence data, children were eligible if aged between 6 months and 10 years. Houses allocated to cluster buffer areas received the same LLINs as those distributed in the core area, but outcome monitoring was done only in households in the core areas.  FOR MALARIA CASE INCIDENCE *note, not all children were eligible for outcome assessment) Pyrethroid-only group: Year 1 (743); Year 2 (842) Pyriproxyfen group: Year 1 (748); Year 2 (844) Chlorfenapyr group: Year 1 (742); Year 2 (845) Piperonyl butoxide group: Year 1 (749); Year 2 (840)  Children 6 months to 10 years (cohort children 2019-2020)  Sex, Female  Pyrethroid-only group: 787/1523 (51·7%)  Pyriproxyfen group: 789/1523 (51·8%)  Chlorfenapyr group: 718/1495 (48·0%)  Piperonyl butoxide group: 825/1527 (54·0%)  FOR SURVEYS FOR PREVALENCE OUTCOMES  *note not all children were eligible for outcome assessment) Pyrethroid-only group: 12 months (1123); 18 months (1127); 24 months (1199)  Pyriproxyfen group: 12 months (1069); 18 months (1153); 24 months (1258) Chlorfenapyr group: 12 months (1126); 18 months (1246); 24 months (1272) Piperonyl butoxide group: 12 months (1071); 18 months (1160); 24 months (1259)  Children 6 months to 14 years (2018)  Median age of selected children, years  Pyrethroid-only group: 6 (3–10), N=1295  Pyriproxyfen group: 6 (3–10), N=1290  Chlorfenapyr group: 6 (3–9), N=1249  Piperonyl butoxide group: 6 (3–10), N=1185  Low socioeconomic status  Pyrethroid-only group: 210/680 (30·9%)  Pyriproxyfen group: 230/667 (34·5%)  Chlorfenapyr group: 209/671 (31·1%)  Piperonyl butoxide group: 237/638 (37·1%) |
| **Frequency of travel in the last month** | They said "A questionnaire was administered to enquire about LLIN use the night before the visit, adverse events, travel history, and visit to health facilities within the past 2 weeks", but gave no data. |
| **INTERVENTION 1** | |
| **Dual AI net brand, insecticide type, dose, material of net**  (including active ingredient, timing and frequency of application, durability of the net and insecticide) | Pyriproxyfen combined with alpha-cypermethrin, Royal Guard |
| **Net treatment strategy**  (e.g., how were the nets treated with the insecticide(s)) | LLINs were treated with a combination of pyriproxyfen (5.5 g/kg) and a-cypermethrin (5.5 g/kg; Royal Guard, Disease Control Technologies, Greer, SC, USA. |
| **Deployment strategy of nets**  (e.g., who received the nets? Where the houses in which they were installed permanent? What was the frequency of distribution?) | All households enumerated in the core and buffer areas of clusters were allocated at least one LLIN for every two people |
| **How was the intervention measured?**  (e.g. how was net usage monitored/observed by the authors? How was coverage of nets measured?) | A survey was done in eight randomly selected houses in each cluster 3 months after distribution to assess LLIN coverage. At 24 months a second survey was conducted that also tested the nets for active ingredient retention. |
| **Coverage of household / person**  (% of households with at least 1 ITN  % of households with at least 1 ITN for every 2 people  % of population with access to an ITN  Nets per HH or per person) | Coverage decreased from 62.2% (2813/4520) at baseline to 38.3% (2087/5455) after 2 years. |
| **Coverage across cluster/site/jurisdiction** | Not reported specifically. Authors mention that all households were targeted and provide the total number of LLINs distributed (147230) but this was not broken down into groups. |
| **Length of intervention and time points of outcome measurement** | LLINs were distributed once, between Jan 26th and Jan 28th 2019. Outcomes were recorded over three years (described below). |
| **Changes in human behaviour** (e.g., sleeping behaviour, non-intervention-based spraying of houses) | Behaviour change communication activities were done during and after the net distribution to increase LLIN use |
| **Background interventions**  (all reported in primary study – e.g., spraying prior to treatment period (and during) including coverage of background interventions. Other malaria or vector-specific control interventions [indoor surface treatment, nets/ other insecticides/ barrier], cointerventions, treatment of individuals that may impact outcome) | Previous vector control interventions in the region included indoor residual spraying and a universal LLIN coverage campaign in 2015, as well as distribution of LLINs to primary school students in 2018 and to pregnant women during antenatal care visits |
| **INTERVENTION 2** | |
| **Dual AI net brand, insecticide type, dose, material of net**  (including active ingredient, timing and frequency of application, durability of the net and insecticide) | Chlorfenapyr combined with alpha-cypermethrin, Interceptor G2 |
| **Net treatment strategy**  (e.g., how were the nets treated with the insecticide(s)) | LLINs were treated with a wash-resistant formulation of 200 mg/  m2 chlorfenapyr and 100 mg/m2 alpha-cypermethrin |
| **Deployment strategy of nets**  (e.g., who received the nets? Where the houses in which they were installed permanent? What was the frequency of distribution?) | All households enumerated in the core and buffer areas of clusters were allocated at least one LLIN for every two people. |
| **How was the intervention measured?**  (e.g. how was net usage monitored/observed by the authors? How was coverage of nets measured?) | A survey was done in eight randomly selected houses in each cluster 3 months after distribution to assess LLIN coverage. At 24 months a second survey was conducted that also tested the nets for active ingredient retention. |
| **Coverage of household / person**  (% of households with at least 1 ITN  % of households with at least 1 ITN for every 2 people  % of population with access to an ITN  Nets per HH or per person) | At least one LLIN was distributed for every two people.  3 months after LLIN distribution, 3155 (72·1%) of 4378 of study participants surveyed reported using the assigned study LLINs the previous night; this proportion was similar between study groups.  Coverage decreased from 59.3% (2849/4803) at baseline to 46.4% (2585/5576) after 2 years |
| **Coverage across cluster/site/jurisdiction** | Not reported specifically. Authors mention that all households were targeted and provide the total number of LLINs distributed (147230) but this was not broken down into groups. |
| **Length of intervention and time points of outcome measurement** | LLINs were distributed once, between Jan 26th and Jan 28th 2019. Outcomes were recorded over three years (described below) and assessed at 12, 18 and 24 months. |
| **Changes in human behaviour** (e.g., sleeping behaviour, non-intervention-based spraying of houses) | Behaviour change communication activities were done during and after the net distribution to increase LLIN use |
| **Background interventions**  (all reported in primary study – e.g., spraying prior to treatment period (and during) including coverage of background interventions. Other malaria or vector-specific control interventions [indoor surface treatment, nets/ other insecticides/ barrier], cointerventions, treatment of individuals that may impact outcome) | Previous vector control interventions in the region included indoor residual spraying and a universal LLIN coverage campaign in 2015, as well as distribution of LLINs to primary school students in 2018 and to pregnant women during antenatal care visits |
| **COMPARISON** | |
| **Dual AI net brand, insecticide type, dose, material of net**  (including active ingredient, timing and frequency of application, durability of the net and insecticide) | There were two comparison groups used in this study.  The first was a pyrethroid-only (reference) group, which received LLINs containing the pyrethroid a-cypermethrin (5 g/kg; Interceptor, BASF SE, Ludwigshafen Germany – INTERCEPTOR LN). The second was a piperonyl butoxide group, with LLINs combining piperonyl butoxide (10 g/kg) and the pyrethroid permethrin (20 g/kg; Olyset Plus, Sumitomo Chemical, Tokyo, Japan). |
| **Net treatment strategy**  (e.g., how were the nets treated with the insecticide(s)) | As above. |
| **Deployment strategy of nets**  (e.g., who received the nets? Where the houses in which they were installed permanent? What was the frequency of distribution?) | All households enumerated in the core and buffer areas of clusters were allocated at least one LLIN for every two people. |
| **How was the intervention measured?**  (e.g. how was net usage monitored/observed by the authors? How was coverage of nets measured?) | A survey was done in eight randomly selected houses in each cluster 3 months after distribution to assess LLIN coverage. At 24 months a second survey was conducted that also tested the nets for active ingredient retention. |
| **Coverage of household / person**  (% of households with at least 1 ITN  % of households with at least 1 ITN for every 2 people  % of population with access to an ITN  Nets per HH or per person) | At least one LLIN was distributed for every two people.  3 months after LLIN distribution, 3155 (72·1%) of 4378 of study participants surveyed reported using the assigned study LLINs the previous night; this proportion was similar between study groups.  Pyrethroid-only group: Coverage decreased from 59.6% (2957/4962) at baseline to 49.5% (2488/5029) after 2 years  PBO group Coverage decreased from 61.7% (2695/4369) at baseline to 29.6% (1534/5185) after 2 years |
| **Coverage across cluster/site/jurisdiction** | Not reported specifically. Authors mention that all households were targeted and provide the total number of LLINs distributed (147230) but this was not broken down into groups. |
| **Length of intervention and time points of outcome measurement** | LLINs were distributed once, between Jan 26th and Jan 28th 2019. Outcomes were recorded over three years (described below) |
| **Changes in human behaviour** (e.g., sleeping behaviour, non-intervention-based spraying of houses) | Behaviour change communication activities were done during and after the net distribution to increase LLIN use |
| **Any other details regarding comparator not described elsewhere.** | Previous vector control interventions in the region included indoor residual spraying and a universal LLIN coverage campaign in 2015, as well as distribution of LLINs to primary school students in 2018 and to pregnant women during antenatal care visits |
| **MALARIA CASE INCIDENCE RATE**  Defined as symptoms plus parasitaemia, over a population at risk or person-time. Detected either through passive or active surveillance. | |
| **Name** | Malaria case incidence |
| **Definition/ assessment metric** | To assess malaria case incidence, 35 households per cluster were enrolled after LLIN distribution in year 1. From each household, one child aged 6 months to 10 years was selected at random and actively followed up for 1 year. A second independent cohort of 40 children per cluster (number increased because of the low incidence in the first year) was recruited 1 year after distribution. Malaria was assessed with a RDT. |
| **Events** | Presented as number of clinical episodes over child-years PYRETHROID-ONLY GROUP Year 1: 194/605.1 (0.32 per year) Year 2: 449/793.4 (0.57 per year) Overall: 643/1398.5 (0.46 per year)  PYRIPROXYFEN GROUP Year 1: 161/604.5 (0.27 per year) Year 2: 418/787.4 (0.53 per year) Overall: 579/1391.9 (0.42 per year)  CHLORENAPYR GROUP Year 1: 79/603.8 (0.13 per year) Year 2: 248/790.6 (0.31 per year) Overall: 317/1394.4 (0.23 per year)  PBO GROUP Year 1: 79/591.8 (0.13 per year) Year 2: 381/788.9 (0.48 per year) Overall: 460/1380.7 (0.33 per year) |
| **Total (or unit time)** | Reported above |
| **Time of outcome assessment** | Reported above |
| **Results**  (i.e., main results – epidemiological, unadjusted and adjusted, secondary outcomes, sensitivity analyses, subgroups, and clusters) | Malaria clinical case incidence over 24 months of follow-up (1380.7 to 1398.5 child-years per group) was 0.46 per child-year in the pyrethroid-only reference group, 0.42 per child-year in the pyriproxyfen group (IRR 0.99 [95% CI 0.66-1.50], p=0.9801), 0.23 per child-year in the chlorfenapyr group (0.56 [0.37-0.86], p=0.0072), and 0.33 per child-year in the piperonyl butoxide group (0.92 [0.61-1.38], p=0.6809) |
| **Effect type** | IRR |
| **MALARIA INFECTION INCIDENCE**  Defined as parasitaemia with or without symptoms, over a population at risk of person-time. Detected through passive or active surveillance. | |
| **Name** | N/A |
| **Definition/ assessment metric** | N/A |
| **Events** | N/A |
| **Total (or unit time)** | N/A |
| **Time of outcome assessment** | N/A |
| **Results**  (i.e., main results – epidemiological, unadjusted and adjusted, secondary outcomes, sensitivity analyses, subgroups, and clusters) | N/A |
| **Effect type** | N/A |
| **INCIDENCE OF SEVERE DISEASE**  Defined as hospitalization with parasitaemia, over a population at risk or person-time. | |
| **Name** | N/A |
| **Definition/ assessment metric** | N/A |
| **Events** | N/A |
| **Total (or unit time)** | N/A |
| **Time of outcome assessment** | N/A |
| **Results**  (i.e., main results – epidemiological, unadjusted and adjusted, secondary outcomes, sensitivity analyses, subgroups, and clusters) | N/A |
| **Effect type** | N/A |
| **PARASITE PREVALENCE**  Parasitaemia with or without symptoms, over the population sampled. Detected through cross-sectional surveys. | |
| **Name** | Parasite prevalence (defined in the study as malaria prevalence) |
| **Definition/ assessment metric** | Prevalence of malaria infection diagnosed with a positive rapid diagnostic test in children aged 6 months to 14 years. This was measured at 12, 18 and 24 months post intervention through a cross-sectional survey. In each household, up to two children aged 6 months to 14 years were randomly selected for detection of malaria parasitaemia. |
| **Events** | PYRETHROID-ONLY GROUP 12 months: 350/1123 18 months: 642/1227 24 months: 549/119  PYRIPROXYFEN GROUP 12 months: 232/1069 18 months:583/1069 24 months: 472/1258  CHLORENAPYR GROUP 12 months: 176/1126 18 months: 509/1246 24 months: 326/1272  PBO GROUP 12 months: 206/1071 18 months: 502/1160 24 months: 512/1259 |
| **Total (or unit time)** | Reported above |
| **Time of outcome assessment** | Reported above |
| **Results**  (i.e., main results – epidemiological, unadjusted and adjusted, secondary outcomes, sensitivity analyses, subgroups, and clusters) | 12 months statistically significant reduction was observed only in the chlorfenapyr group and not in the pyriproxyfen or piperonyl butoxide groups  18 months no group showed a significant reduction relative to the pyrethroid-only group at 18 months  24 months No statistically significant reduction in the prevalence of malaria infection was observed in the pyriproxyfen group (472 [37.5%] of 1258 children; aOR 0.79 [95% CI 0.54-1.17], p=0.2354) or the piperonyl butoxide group (512 [40.7%] of 1259; 0.99 [0.67-1.45], p=0.9607) relative to the pyrethroid-only reference group (549 [45.8%] of 1199); however, prevalence was significantly lower in the chlorfenapyr group (326 [25.6%] of 1272; 0.45 [0.30-0.67], p=0.0001) than in the pyrethroid-only group. |
| **Effect type** | OR or RR |
| **ALL-CAUSE MORTALITY**  Number of deaths over the population at risk or person-time. | |
| **Name** | All cause mortality |
| **Definition/ assessment metric** | Number of deaths over the population at risk or person-time |
| **Events** | PYRETHROID-ONLY GROUP Year 1: 0/743 Year 2: 2/842  PYRIPROXYFEN GROUP Year 1: 0/748 Year 2: 2/844  CHLORENAPYR GROUP Year 1: 1/742 Year 2: 0/845  PBO GROUP Year 1: 0/749 Year 2: 0/840 |
| **Total (or unit time)** | Reported above |
| **Time of outcome assessment** | Reported above |
| **Results**  (i.e., main results – epidemiological, unadjusted and adjusted, secondary outcomes, sensitivity analyses, subgroups, and clusters) | Five deaths among cohort children were reported. While these deaths have been reported per group and year, the reasons have not been separated by group or year. As reported by the authors three deaths were from drowning, one was due to severe malaria, and one due to pneumonia, all of which were judged to be unrelated to the study interventions. |
| **Effect type** | RR or OR |
| **MALARIA MORTALITY**  Number of deaths attributed to malaria over the population at risk or person-time. | |
| **Name** | Malaria mortality |
| **Definition/ assessment metric** | Number of deaths attributed to malaria over the population at risk or person-time. |
| **Events** | Reported below |
| **Total (or unit time)** | Reported below |
| **Time of outcome assessment** | Reported below |
| **Results**  (i.e., main results – epidemiological, unadjusted and adjusted, secondary outcomes, sensitivity analyses, subgroups, and clusters) | There was only 1 death attributed to malaria throughout the entire study. However, this death has not been categorized to treatment group, or year. |
| **Effect type** | Not synthesizable |
| **PREVALENCE OF ANAEMIA**  Defined by study thresholds of anaemia. | |
| **Name** | Prevalence of anaemia |
| **Definition/ assessment metric** | Moderate or severe anaemia was defined as haemoglobin concentration <8 g/dL. This was measured in children 6 months to 4 years measured at 12, 18, and 24 months during the cross-sectional surveys. In each household, up to two children aged 6 months to 14 years were randomly selected for anaemia assessment. |
| **Events** | PYRETHROID-ONLY GROUP 12 months: 7/1123 18 months: 45/1227 24 months: 29/119  PYRIPROXYFEN GROUP 12 months: 8/1069 18 months: 37/1069 24 months: 38/1258  CHLORENAPYR GROUP 12 months: 11/1126 18 months: 33/1246 24 months: 28/1272  PBO GROUP 12 months: 10/1071 18 months: 23/1160 24 months: 26/1259 |
| **Total (or unit time)** | Reported above |
| **Time of outcome assessment** | Reported above |
| **Results**  (i.e., main results – epidemiological, unadjusted and adjusted, secondary outcomes, sensitivity analyses, subgroups, and clusters) | No significant difference in moderate and severe anaemia was observed in any of the intervention groups at any time point |
| **Effect type** | RR or OR |
| **COSTS** | |
| **Event/raw costs** | Costs- constant USD  Standard 2-yearly  Cases: 4,785  DALYs: 412  Societal - 50,329  Household - 22,486  Public provider - 28,040  Donor - 24,281  PBO 2-yearly  Cases: 4,378  DALYs: 375  Societal - 53,007  Household - 20,569  Public provider - 32,856  Donor - 29,333  Pyriproxyfen 2-yearly  Cases: 4,886  DALYs: 422  Societal - 60,410  Household - 22,808  Public provider - 37,661  Donor - 33,810  Chlorfenapyr 2-yearly  Cases: 3,026  DALYs: 260  Societal - 45,016  Household - 14,087  Public provider - 30,934  Donor - 28,585 |
| **Results** | Chlorfenapyr LLINs were estimated to avert the most DALYs (mean 152 DALYs averted [SD 72] per 10 000 total population), followed by piperonyl butoxide LLINs (37 DALYs averted [72] per 10 000 population), while pyriproxyfen LLINs incurred 9 more DALYs [71] per 10 000 population than did pyrethroid-only LLINs.  From public provider and donor perspectives, chlorfenapyr LLINs would be the most cost-effective of the three dual-active-ingredient LLINs, costing an additional $19 (95% uncertainty interval 1-105) to public providers or $28 (11-120) to donors per DALY averted relative to pyrethroid-only LLINs-well below plausible costeffectiveness thresholds ($292-393 per DALY averted) |
| **Time of cost assessment** | Cost effectiveness was modelled over the 2-year trial. Malaria incidence estimates for each trial year were combined with probabilities of progression to severe disease and death on the basis of secondary sources |
| **Resources needed/ used** | Nets and active ingredients |
| **ADDITIONAL DATA** | |
| **Unintended benefits** | Not reported |
| **Harms** | Side-effect related to use of standard pyrethroid-only LLINs were reported in 90 (44.1%) of 204 participants at 3 months post-distribution, 167 (10.1%) of 1647 at 12 months, two (0.1%) of 1579 at 18 months, and 11 (0.7%) of 1683 at 24 months.  Pyriproxyfen LLINs side-effects were reported in 80 (38.8%) of 206 participants at 3 months, 143 (9.3%) of 1543 at 12 months, none of 1425 at 18 months, and seven (0.4%) of 1692 at 24 months.  Piperonyl butoxide group (17 [8.5%] of 199) at 3 months  Chlorfenapyr group (17 [8.5%] of 199) at 3 months   Skin irritation or paraesthesia was the most commonly reported side-effect in all groups, reported in 497 (83.1%) of 598 participants who reported side-effects. No serious or severe side effects were reported. |
| **Other contextual information present/measured/reported)** (feasibility, acceptability, preferences/values, impact on equity) | The proportion of study LLINs that were torn (defined as hole area ≥790 cm²)23 was 86 (28%) of 303 in the pyrethroid-only group, 109 (39%) of 282 in the pyriproxyfen group, 96 (34%) of 284 in the chlorfenapyr group, and 81 (43%) of 188 in the piperonyl butoxide group. Chemical analysis on 30 nets per group showed that the active ingredient concentration in each type of LLIN met the specification criteria when new. At 24 months, partner active ingredient retention was 28% for pyriproxyfen, 18% for chlorfenapyr, and 30% for piperonyl butoxide. |
| **Entomological outcomes measured** (list) | Entomological inoculation rate Vector density Sporozoite rate Insecticide resistance |
| **Other** | Not reported. |
| **Source of funding** | The trial was funded by UK Research and Innovation Joint Global Health Trials programme (reference number MR/R006040/1). |
| **Possible conflicts of interest** | The authors declared no conflicts of interest. The funders of the study had no role in study design, data collection, data analysis, data interpretation, or writing of the report. |

| **Domain** | **Signalling question** | **Response** | **Comments** |
| --- | --- | --- | --- |
| **Bias arising from the randomization process** | 1a.1 Was the allocation sequence random? | Y | An independent statistician conducted constrained randomisation to allocate the 84 clusters to the four study  groups at a ratio of 1:1:1:1, ensuring that absolute differences in cluster means between study groups were  within the specified ranges for specific cluster characteristics.   Randomisations was verified by checking the frequency of allocations to the same study group of all pairs of clusters.  The inhabitants of each cluster and the field staff who were responsible for enrolment and collected data were masked to the type of LLIN allocated. LLINs of each type were similar in appearance apart from a colour-coded  loop and a unique identifying code. |
|  | 1a.2 Was the allocation sequence concealed until clusters were enrolled and assigned to interventions? | Y |  |
|  | 1a.3 Did baseline differences between intervention groups suggest a problem with the randomization process? | N | No differences detected |
|  | **Risk of bias judgement** | **Low** | An independent statistician conducted constrained randomisation to allocate the 84 clusters to the four study groups at a ratio of 1:1:1:1, ensuring that absolute differences in cluster means between study groups were within the specified ranges for specific cluster characteristics.  Randomisations was verified by checking the frequency of allocations to the same study group of all pairs of clusters. The inhabitants of each cluster and the field staff who were responsible for enrolment and collected data were masked to the type of LLIN allocated. LLINs of each type were similar in appearance apart from a colour-coded loop and a unique identifying code. No differences detected |
| **Bias arising from the randomization process (FOR PYRETHROID-PBO COMPARISONS)** | 1a.1 Was the allocation sequence random? | Y | An independent statistician conducted constrained randomisation to allocate the 84 clusters to the four study  groups at a ratio of 1:1:1:1, ensuring that absolute differences in cluster means between study groups were  within the specified ranges for specific cluster characteristics.   Randomisations was verified by checking the frequency of allocations to the same study group of all pairs of clusters.  The inhabitants of each cluster and the field staff who were responsible for enrolment and collected data were masked to the type of LLIN allocated. LLINs of each type were similar in appearance apart from a colour-coded  loop and a unique identifying code. |
|  | 1a.2 Was the allocation sequence concealed until clusters were enrolled and assigned to interventions? | Y |  |
|  | 1a.3 Did baseline differences between intervention groups suggest a problem with the randomization process? | N | No differences detected |
|  | **Risk of bias judgement** | **High** | While randomisation techniques were the same as described above, the comparative data for DAI ITNs against pyrethroid-PBO ITNs has not considered the ICC in the analysis. Therefore there is a high risk of bias. |
| **Bias arising from the timing of identification or recruitment of participants** | 1b.1 Were all the individual participants identified and recruited (if appropriate) before randomization of clusters? | **Y** | Participants belonged to households which were then segmented into clusters based on a mapping census |
|  | 1b.2 If N/PN/NI to 1b.1: Is it likely that selection of individual participants was affected by knowledge of the intervention assigned to the cluster? | **NA** |  |
|  | 1b.3 Were there baseline imbalances that suggest differential identification or recruitment of individual participants between intervention groups? |  | N |
|  | **Risk of bias judgement** | **Low** | Participants belonged to households which were then segmented into clusters based on a mapping census |
| **Bias due to deviations from intended interventions** | 2.1a Were participants aware that they were in a trial? | Y | Informed consent was given by all participants |
|  | 2.1b If Y/PY/NI to 2.1a: Were participants aware of their assigned intervention during the trial? | N | The inhabitants of each cluster and the field staff who were responsible for enrolment and collected data were masked to the type of LLIN allocated. LLINs of each type were similar in appearance apart from a colour-coded loop and a unique identifying code. |
|  | 2.2 Were carers and people delivering the interventions aware of participants' assigned intervention during the trial? | N |  |
|  | 2.3 If Y/PY/NI to 2.1b or 2.2: Were there deviations from the intended intervention that arose because of the trial context? | NA |  |
|  | 2.4 If Y/PY to 2.3: Were these deviations likely to have affected the outcome? | NA |  |
|  | 2.5 If Y/PY/NI to 2.4: Were these deviations from intended intervention balanced between groups? | NA |  |
|  | 2.6 Was an appropriate analysis used to estimate the effect of assignment to intervention? | Y | ITT was used throughout the study |
|  | 2.7 If N/PN/NI to 2.6: Was there potential for a substantial impact (on the result) of the failure to analyse participants in the group to which they were randomized ? | NA |  |
|  | **Risk of bias judgement** | **Low** | Informed consent was given by all participants  The inhabitants of each cluster and the field staff who were responsible for enrolment and collected data were masked to the type of LLIN allocated. LLINs of each type were similar in appearance apart from a colour-coded loop and a unique identifying code.  ITT was used throughout the study |
| **Outcome: Malaria Case Incidence** | | | |
| **Bias due to missing outcome data** | 3.1a Were data for this outcome available for all clusters that recruited participants? | Y | To assess malaria case incidence, 35 households per cluster were enrolled after LLIN distribution in year 1. From each household, one child aged 6 months to 10 years was selected at random and actively followed up for 1 year. A second independent cohort of 40 children per cluster (number increased because of the low incidence in the first year) was recruited 1 year after distribution. |
|  | 3.1b Were data for this outcome available for all, or nearly all, participants within clusters? | N |  |
|  | 3.2 If N/PN/NI to 3.1a or 3.1b: Is there evidence that the result was not biased by missing data? | N | No evidence that the results was not biased |
|  | 3.3 If N/PN to 3.2 Could missingness in the outcome depend on its true value? | N | Children were randomly selected, so it is unlikely that missingness in the outcome may depend on its true value. |
|  | 3.4 If Y/PY/NI to 3.3: Is it likely that missingness in the outcome depended on its true value? | NA |  |
|  | **Risk of bias judgement** | **Low** | To assess malaria case incidence, 35 households per cluster were enrolled after LLIN distribution in year 1. From each household, one child aged 6 months to 10 years was selected at random and actively followed up for 1 year. A second independent cohort of 40 children per cluster (number increased because of the low incidence in the first year) was recruited 1 year after distribution.  No evidence that the results was not biased  Children were randomly selected, so it is unlikely that missingness in the outcome may depend on its true value |
| **Bias in measurement of the outcome** | 4.1 Was the method of measuring the outcome inappropriate? | N | Prevalence of malaria infection diagnosed with a positive rapid diagnostic test |
|  | 4.2 Could measurement or ascertainment of the outcome have differed between intervention groups? | N |  |
|  | 4.3a If N/PN/NI to 4.1 and 4.2: Were outcome assessors aware that a trial was taking place? | Y |  |
|  | 4.3b If Y/PY/NI to 4.3a: Were outcome assessors aware of the intervention received by study participants? | N | The inhabitants of each cluster and the field staff who were responsible for enrolment and collected data were masked to the type of LLIN allocated. LLINs of each type were similar in appearance apart from a colour-coded loop and a unique identifying code. |
|  | 4.4 If Y/PY/NI to 4.3b: Could assessment of the outcome have been influenced by knowledge of intervention received? | NA |  |
|  | 4.5 If Y/PY/NI to 4.4: Is it likely that assessment of the outcome was influenced by knowledge of intervention received? | NA |  |
|  | **Risk of bias judgement** | **Low** | Prevalence of malaria infection diagnosed with a positive rapid diagnostic test  The inhabitants of each cluster and the field staff who were responsible for enrolment and collected data were masked to the type of LLIN allocated. LLINs of each type were similar in appearance apart from a colour-coded loop and a unique identifying code. |
| **Bias in selection of the reported result** | 5.1 Were the data that produced this result analysed in accordance with a pre-specified analysis plan that was finalized before unblinded outcome data were available for analysis? | Y | Protocol provided and the results present align.  Some outcomes have not been fully reported, but that is because the trial is ongoing for outcomes longer than 2 years post intervention. |
|  | 5.2 ... multiple eligible outcome measurements (e.g. scales, definitions, time points) within the outcome domain? | N | Yes, but does not impact bias, as all time points were reported in full |
|  | 5.3 ... multiple eligible analyses of the data? | N |  |
|  | **Risk of bias judgement** | **Low** | Protocol provided and the results present align.  Some outcomes have not been fully reported, but that is because the trial is ongoing for outcomes longer than 2 years post intervention. |
| **Outcome: Parasite Prevalence** | | | |
| **Bias due to missing outcome data** | 3.1a Were data for this outcome available for all clusters that recruited participants? | Y | Prevalence of malaria infection diagnosed with a positive rapid diagnostic test in children aged 6 months to 14 years. In each household, up to two children aged 6 months to 14 years were randomly selected for detection of malaria parasitaemia. |
|  | 3.1b Were data for this outcome available for all, or nearly all, participants within clusters? | N |  |
|  | 3.2 If N/PN/NI to 3.1a or 3.1b: Is there evidence that the result was not biased by missing data? | N | No evidence that the results was not biased |
|  | 3.3 If N/PN to 3.2 Could missingness in the outcome depend on its true value? | N | Children were randomly selected, so it us unlikely that missingness in the outcome may depend on its true value |
|  | 3.4 If Y/PY/NI to 3.3: Is it likely that missingness in the outcome depended on its true value? | NA |  |
|  | **Risk of bias judgement** | **Low** | Prevalence of malaria infection diagnosed with a positive rapid diagnostic test in children aged 6 months to 14 years. In each household, up to two children aged 6 months to 14 years were randomly selected for detection of malaria parasitaemia.   No evidence that the results was not biased  Children were randomly selected, so it us unlikely that missingness in the outcome may depend on its true value |
| **Bias in measurement of the outcome** | 4.1 Was the method of measuring the outcome inappropriate? | N | Prevalence of malaria infection diagnosed with a positive rapid diagnostic test |
|  | 4.2 Could measurement or ascertainment of the outcome have differed between intervention groups? | N |  |
|  | 4.3a If N/PN/NI to 4.1 and 4.2: Were outcome assessors aware that a trial was taking place? | Y |  |
|  | 4.3b If Y/PY/NI to 4.3a: Were outcome assessors aware of the intervention received by study participants? | N | The inhabitants of each cluster and the field staff who were responsible for enrolment and collected data were masked to the type of LLIN allocated. LLINs of each type were similar in appearance apart from a colour-coded loop and a unique identifying code. |
|  | 4.4 If Y/PY/NI to 4.3b: Could assessment of the outcome have been influenced by knowledge of intervention received? | NA |  |
|  | 4.5 If Y/PY/NI to 4.4: Is it likely that assessment of the outcome was influenced by knowledge of intervention received? | NA |  |
|  | **Risk of bias judgement** | **Low** | Prevalence of malaria infection diagnosed with a positive rapid diagnostic test  The inhabitants of each cluster and the field staff who were responsible for enrolment and collected data were masked to the type of LLIN allocated. LLINs of each type were similar in appearance apart from a colour-coded loop and a unique identifying code. |
| **Bias in selection of the reported result** | 5.1 Were the data that produced this result analysed in accordance with a pre-specified analysis plan that was finalized before unblinded outcome data were available for analysis? | Y | Protocol provided and the results present align.  Some outcomes have not been fully reported, but that is because the trial is ongoing for outcomes longer than 2 years post intervention. |
|  | 5.2 ... multiple eligible outcome measurements (e.g. scales, definitions, time points) within the outcome domain? | N | Yes, but does not impact bias, as all time points were reported in full |
|  | 5.3 ... multiple eligible analyses of the data? | N |  |
|  | **Risk of bias judgement** | **Low** | Protocol provided and the results present align.  Some outcomes have not been fully reported, but that is because the trial is ongoing for outcomes longer than 2 years post intervention.  But does not impact bias, as all time points were reported in full |
| **Outcome: Prevalence of Anaemia** | | | |
| **Bias due to missing outcome data** | 3.1a Were data for this outcome available for all clusters that recruited participants? | Y |  |
|  | 3.1b Were data for this outcome available for all, or nearly all, participants within clusters? | N | Moderate or severe anaemia was defined as haemoglobin concentration <8 g/dL. This was measured in children 6 months to 4 years measured at 12, 18, and 24 months during the cross-sectional surveys. In each household, up to two children aged 6 months to 14 years were randomly selected for anaemia assessment. |
|  | 3.2 If N/PN/NI to 3.1a or 3.1b: Is there evidence that the result was not biased by missing data? | N | No evidence that the results was not biased |
|  | 3.3 If N/PN to 3.2 Could missingness in the outcome depend on its true value? | N | Children were randomly selected, so it us unlikely that missingness in the outcome may depend on its true value |
|  | 3.4 If Y/PY/NI to 3.3: Is it likely that missingness in the outcome depended on its true value? | NA |  |
|  | **Risk of bias judgement** | **Low** | Moderate or severe anaemia was defined as haemoglobin concentration <8 g/dL. This was measured in children 6 months to 4 years measured at 12, 18, and 24 months during the cross-sectional surveys. In each household, up to two children aged 6 months to 14 years were randomly selected for anaemia assessment.  No evidence that the results was not biased  Children were randomly selected, so it us unlikely that missingness in the outcome may depend on its true value |
| **Bias in measurement of the outcome** | 4.1 Was the method of measuring the outcome inappropriate? | N | Moderate or severe anaemia was defined as haemoglobin concentration <8 g/dL. This was measured in children 6 months to 4 years measured at 12, 18, and 24 months during the cross-sectional surveys. In each household, up to two children aged 6 months to 14 years were randomly selected for anaemia assessment. |
|  | 4.2 Could measurement or ascertainment of the outcome have differed between intervention groups? | N |  |
|  | 4.3a If N/PN/NI to 4.1 and 4.2: Were outcome assessors aware that a trial was taking place? | Y |  |
|  | 4.3b If Y/PY/NI to 4.3a: Were outcome assessors aware of the intervention received by study participants? | N | The inhabitants of each cluster and the field staff who were responsible for enrolment and collected data were masked to the type of LLIN allocated. LLINs of each type were similar in appearance apart from a colour-coded loop and a unique identifying code. |
|  | 4.4 If Y/PY/NI to 4.3b: Could assessment of the outcome have been influenced by knowledge of intervention received? | NA |  |
|  | 4.5 If Y/PY/NI to 4.4: Is it likely that assessment of the outcome was influenced by knowledge of intervention received? | NA |  |
|  | **Risk of bias judgement** | **Low** | Moderate or severe anaemia was defined as haemoglobin concentration <8 g/dL. This was measured in children 6 months to 4 years measured at 12, 18, and 24 months during the cross-sectional surveys. In each household, up to two children aged 6 months to 14 years were randomly selected for anaemia assessment.  The inhabitants of each cluster and the field staff who were responsible for enrolment and collected data were masked to the type of LLIN allocated. LLINs of each type were similar in appearance apart from a colour-coded loop and a unique identifying code. |
| **Bias in selection of the reported result** | 5.1 Were the data that produced this result analysed in accordance with a pre-specified analysis plan that was finalized before unblinded outcome data were available for analysis? | Y | Protocol provided and the results present align.  Some outcomes have not been fully reported, but that is because the trial is ongoing for outcomes longer than 2 years post intervention. |
|  | 5.2 ... multiple eligible outcome measurements (e.g. scales, definitions, time points) within the outcome domain? | N | Yes, but does not impact bias, as all time points were reported in full |
|  | 5.3 ... multiple eligible analyses of the data? | N |  |
|  | **Risk of bias judgement** | **Low** | Protocol provided and the results present align.  Some outcomes have not been fully reported, but that is because the trial is ongoing for outcomes longer than 2 years post intervention.  But does not impact bias, as all time points were reported in full |

## Tiono 2018

| **Study:** | |
| --- | --- |
| **DESIGN** | |
| **Lead Author** | Alfred B Tiono |
| **Publication year** | 2018 |
| **Trial name** | AvecNet Trial |
| **Other reports of this study**  (i.e., author, year, title, doi) | Sagnon 2015 (Trial Protocol 1)  Tiono 2015 (Trail Protocol 2)  Tiono 2016 (Conference Presentation)  Tiono 2018b (Supplementary Material to Manuscript) |
| **Time period study was conducted** | Protocol 1: 2014 to 2017.  Protocol 2: May 2014 to May 2015. |
| **Design** | Two-group, step wedge,  cluster-randomised, controlled, superiority trial |
| **Eligibility criteria of participants** | Protocol 2: Children resident in Burkina Faso, aged 6 months to 5 years old.  Eligibility criteria:  Resident children, aged 6 months to 5 years old will be enumerated and an average of 50 per cluster (range 30-100, depending on village size) will be selected randomly, stratified by age and invited to participate in the clinical surveys and passive case detection (PCD). No distinctions will be made regarding gender, ethnic group, medical condition or physical health.  -Gender:Both  -Target number of participants: 2000  -Participant exclusion criteria: Those who have not provided their consent for inclusion in the study |
| **Recruitment methods and rate** | Protocol 1: The study villages will be enumerated following normal demographic surveillance procedures and compounds mapped before being randomised to net type.  Protocol 2: Recruitment start date: 01/05/2014  Recruitment end date: 01/05/2015 |
| **Unit of allocation** | Cluster (consisting of one to four neighbouring villages, aka compound) |
| **Method of Randomization/ matching or other** | Random selection was done with Stata version 10. five randomly selected clusters of villages were provided with PPF-treated LLINs and the remaining 35 village clusters with standard LLINs |
| **Number of clusters per arm** | This was stepped wedged trial. In the first month, only 5 clusters were randomly selected to receive the PPF-LLINs. Then five clusters were randomly selected each month from July, 2014, to September, 2014, for replacement of standard LLINs with PPF-treated LLINs, so that, by the end of 2014, each study group had an equal number of clusters. By September of 2015, every cluster had received the PPF-LLIN.  Step-wedge design was utilized because it represented the type of deployment used by net distribution programmes. |
| **Adjustment for clustering** | Poisson regression models were used, with log-transformed time at risk as an offset, and with inclusion of village cluster as a random effect and calendar month and health facility as fixed effects.  Protocol 2: Adjusting for a coefficient of up to 0.5 for the differences  in cluster size. Within each model, effect size (that is, difference between PPF-LLIN and LLIN clusters) will, as appropriate, be adjusted for clustering within the groups of villages, the shortest distance between each PPF-LLIN cluster of villages and its nearest control, village-cluster size, and village-cluster. |
| **Cluster details**  (including buffer sizes between clusters, other indication of dilution effects) | Number of clusters: 40  Number of villages: 91  Number of households: 6062  Protocol 2: i) LLINs are a community-level intervention; and ii) the village cluster is a suitable unit for randomization since they represent a discrete spatial cluster.  It is important that within the study area there are no villages that are not enrolled in the study in order for the PPF-LLIN to have maximum impact on larval populations and minimize the spill-over of mosquitoes from villages without these nets.  The main endpoint analysis will adjust for distance between village clusters with different types of nets. This adjustment is critical since the impact of PPF-LLIN is likely to increase as they cover larger areas and so reduce the spill over of mosquitoes from villages with LLINs. |
| **Number of participants per arm** (including number of exclusions and reasons) | At baseline 1980 children were enrolled into 40 clusters. However, by the fourth survey at the end of study, the cohort comprised 2148 children. A mean of 46–56 additional children per cluster were included in surveys two to four.  Protocol 2: A minimum of 40 children per cluster (range 40 to 75), depending on cluster size, will be randomly selected, stratified by age and invited to participate in the clinical and travel/residence surveys and passive case detection (PCD). |
| **Outcomes assessed** | Malaria case incidence rate  Parasite prevalence  All-cause mortality  Prevalence of anaemia |
| **SETTING** | |
| **Country** | Burkina Faso |
| **Site/s**  (town/settlements/region) | Protocol 1: The study will be carried out in two villages in the Cascades  Region of Burkina Faso.  Dalamba (village A)  Sanako (village B)  Protocol 2: The study site is situated south of the road from Banfora and Sideradougou (10° 56’ 00” N, 004° 46’ 00” W) and is approximately 1,250 km2, bisected by a river in a north- south  axis. |
| **Peak transmission season** | Rainy season from May to October with little rain in other months. This defines the seasonal malaria transmission with most malaria episodes  experienced during or immediately following the rainy season. |
| **Baseline malaria endemicity/ level of transmission** | South-east of Banfora town in Burkina Faso, a country where malaria is relatively stable but highly endemic.  Malaria prevalence dropped by half and the incidence of clinical disease fell by 40% from 2000-15. Burkina Faso, with more than 10 million uncomplicated cases of malaria annually, is one of 20 sub-Saharan countries where malaria cases increased between 2015 and 2016. 61% of children aged six months to five years infected in 2014. |
| **Study site (e.g., rural/ urban/ peri-urban/ level of urbanicity)** | Urbanicity was not specifically reported. However, the authors stated that most people were living in small rural villages in houses made  with mud or cement walls and thatched or metal roofs. |
| **Vector species and vector profile details**  (i.e., behaviours, resistance profile/ susceptibility tests, parity, sporozoite rates and all other reported information) | *A gambiae* sensu stricto and *Anopheles coluzzii* were the two most common vectors between groups.  The authors discuss in the introduction that these vectors are highly resistant but have not conducted any resistance testing themselves in this paper.  They have presented the following information regarding parity and sporozoite proportions, by each group:  PROPORTION OF PAROUS MOSQUITOES  Standard LLIN = 60% (625/1038)  PPF LLIN = 62% (1364/2198)  PROPORTION OF MOSQUITOES WITH SPOROZOITES  Standard LLIN = 4% (206/4858)  PPF LLIN = 3% (273/8935) |
| **Malaria species** | *P falciparum* |
| **PARTICIPANTS (those who received the intervention and on whom impact was measured)** | |
| **Characteristics, numbers, and demographics of participants who received the intervention** | 1980 total children were enrolled in the study.  Data are n (%), median (IQR) or n/N (%)  Girls = 962 (49%)  Boys = 1018 (51%)  Age (months) = 35 (22-48)  Sleeps under a mosquito net = 1828 (92%)  Received antimalarials in past 14 days = 143 (7%)  Sick with a fever during past 48h = 250 (13%)  Axillary temp degC = 36.6 (36.3-36.0)  Positive RDT = 226/321 (70%)  Presence of P. falciparum parasites by microscopy = 981/1918 (51%)  >5000 P falciparum parasites per uL = 271/1918 (14%)  P falciparum parasite density (per UL) = 1698 (6.1) (Geometric mean and SD)  Presence of P falciparum gametocytes = 380/1918 (20%)  Haemoglobin (g/L) = 102.0 (94.0-1110.0)  Moderate anaemia = 131/1800 (7%)  Severe anaemia = 2/1800 (0%) |
| **Characteristics, numbers, and demographics of participants on whom the impact was measured.** | Only available for replacement children enrolled into study at third survey (totals below).  Female, N (%) 342/675 (51%)  Age (months), median (IQR): 29 (20,42)  Sleeps under a mosquito net, N (%): 669/675 (99%)  Took anti-malarials in last 14 days, N (%): 5/675 (1%)  Sick with a fever during previous 48 hours N (%): 29/675 (4%)  Positive rapid diagnostic test, N (%): 24/37 (65%)  Supplementary Material: At the first survey, 1,980 children were enrolled in the cohort, with 675 children added at the third survey to replace those lost or exited. By the fourth survey at the end of study, there were 2,148 children in the cohort. An average of 46-56 additional children per cluster were included in surveys two to four. |
| **Frequency of travel in the last month** | Protocol 2: History of travel away from the village for periods of over a week will be captured by the monthly surveys and time at risk will be censored for such periods. In addition, malaria cases in children who resided outside their study village for more than half the elapsed study period at the time of illness will be censored. However this was not reported. |
| **INTERVENTION (Repeat if multiple arms)** | |
| **Dual AI net brand, insecticide type, dose, material of net**  (including active ingredient, timing and frequency of application, durability of the net and insecticide) | PPF-LLIN  The nets contain 2% w/w permethrin and 1% w/w pyriproxyfen incorporated into polyethylene fibres giving adequate release of permethrin and pyriproxyfen for an estimated 3 years (Sumitomo Chemical)  The PPF-LLINs were distributed according to the step-wedged design detailed above.  The nets were white and sized at 1.8m wide by 1.9m long by 15m high |
| **Net treatment strategy**  (e.g., how were the nets treated with the insecticide(s)) | Chemical content of 30 randomly selected LLINs and 30 PPF-treated LLINs was checked with high-performance liquid chromatography  at the Liverpool School of Tropical Medicine (Liverpool, UK), which confirmed the target doses. |
| **Deployment strategy of nets**  (e.g., who received the nets? Where the houses in which they were installed permanent? What was the frequency of distribution?) | Nets were distributed to achieve one LLIN per bed or sleeping place at the beginning of the transmission season in 2014. Each month for the next 4 months after LLIN donation, residents in 5 randomly selected village clusters will be asked to exchange their LLINs for PPF-LLIN. (According to stepped-wedge design) |
| **How was the intervention measured?**  (e.g. how was net usage monitored/observed by the authors? How was coverage of nets measured?) | Coverage was assessed as number of children who were sleeping under a net, at each survey (4 different time points, survey 1 – June 2014, survey 2 – December 2014, survey 3 – May 2015, survey 4 – July 2015). |
| **Coverage of household / person**  (% of households with at least 1 ITN  % of households with at least 1 ITN for every 2 people  % of population with access to an ITN  Nets per HH or per person) | 29 084 LLINs (24 357 standard and 4727 PPF treated) were distributed to cover the 30 608 sleeping places identified during the pre-study population census, yielding an overall coverage of 95%.  This decreased to 92% in survey 1, but then increased to greater than 99% in surveys 2, 3 and 4. |
| **Coverage across cluster/site/jurisdiction** | Coverage exceeded 80% in all clusters except for one in which residents’ multiple absences from home for traditional gold-mining activities made it impossible to deliver nets despite numerous attempts |
| **Length of intervention and time points of outcome measurement** | Outcomes were measured at 4 different time points (for the cross-sectional surveys, described above).  Length of the intervention lasted from June 2014 to December 2015. However as this was a stepped-wedge design, not all clusters experienced the intervention for the same time. (detailed above). |
| **Changes in human behaviour** (e.g., sleeping behaviour, non-intervention-based spraying of houses) | Collected according to protocol 2, however not reported |
| **Background interventions**  (all reported in primary study – e.g., spraying prior to treatment period (and during) including coverage of background interventions. Other malaria or vector-specific control interventions [indoor surface treatment, nets/ other insecticides/ barrier], cointerventions, treatment of individuals that may impact outcome) | Burkina Faso Government’s Roll Back Malaria information, education, and communication procedures were followed to encourage correct net use and maintenance for both type of nets. |
| **COMPARISON** | |
| **Dual AI net brand, insecticide type, dose, material of net**  (including active ingredient, timing and frequency of application, durability of the net and insecticide) | Standard LLIN  2% w/w permethrin incorporated into polyethylene fibres giving adequate release of permethrin for about 5 years.  The PPF-LLINs were distributed according to the step-wedged design detailed above.  The nets were white and sized at 1.8m wide by 1.9m long by 15m high |
| **Net treatment strategy**  (e.g., how were the nets treated with the insecticide(s)) | Chemical content of 30 randomly selected LLINs and 30 PPF-treated LLINs was checked with high-performance liquid chromatography  at the Liverpool School of Tropical Medicine (Liverpool, UK), which confirmed the target doses. |
| **Deployment strategy of nets**  (e.g., who received the nets? Where the houses in which they were installed permanent? What was the frequency of distribution?) | Nets were distributed to achieve one LLIN per bed or sleeping place at the beginning of the transmission season in 2014 |
| **How was the intervention measured?**  (e.g. how was net usage monitored/observed by the authors? How was coverage of nets measured?) | Coverage was assessed as number of children who were sleeping under a net, at each survey (4 different time points, survey 1 – June 2014, survey 2 – December 2014, survey 3 – May 2015, survey 4 – July 2015). |
| **Coverage of household / person**  (% of households with at least 1 ITN  % of households with at least 1 ITN for every 2 people  % of population with access to an ITN  Nets per HH or per person) | 29 084 LLINs (24 357 standard and 4727 PPF treated) were distributed to cover the 30 608 sleeping places identified during the pre-study population census, yielding an overall coverage of 95%.  This decreased to 92% in survey 1, but then increased to greater than 99% in surveys 2, 3 and 4. |
| **Coverage across cluster/site/jurisdiction** | Coverage exceeded 80% in all clusters except for one in which residents’ multiple absences from home for traditional gold-mining activities made it impossible to deliver nets despite numerous attempts |
| **Length of intervention and time points of outcome measurement** | Outcomes were measured at 4 different time points (for the cross-sectional surveys, described above).  Length of the intervention lasted from June 2014 to December 2015. However as this was a stepped-wedge design, not all clusters experienced the intervention for the same time. (detailed above). |
| **Changes in human behaviour** (e.g., sleeping behaviour, non-intervention-based spraying of houses) | Not reported |
| **Any other details regarding comparator not described elsewhere.** | Burkina Faso Government’s Roll Back Malaria information, education, and communication procedures were followed to encourage correct net use and maintenance for both type of nets. |
| **MALARIA CASE INCIDENCE RATE**  Defined as symptoms plus parasitaemia, over a population at risk or person-time. Detected either through passive or active surveillance. | |
| **Name** | As above |
| **Definition/ assessment metric** | Incidence of clinical episodes of malaria among cohort children presenting at health facilities. Confirmed through presence of a fever and RDT. |
| **Events** | Malaria Episodes  Standard LLIN = 1691  PPF-LLIN = 2047 |
| **Total (or unit time)** | Years of Exposure  Standard LLIN = 844  PPF-LLIN = 1351 |
| **Time of outcome assessment** | Ongoing reporting throughout duration of trial |
| **Results**  (i.e., main results – epidemiological, unadjusted and adjusted, secondary outcomes, sensitivity analyses, subgroups, and clusters) | The overall incidence of clinical malaria was 1.5 per child-year at risk in the PPF-treated LLIN group versus 2.0 per child-year at risk in the LLIN group.  The adjusted IRR (adjusted for cluster, month and health facility was 0.88 (0.77-0.99) |
| **Effect type** | IRR |
| **MALARIA INFECTION INCIDENCE**  Defined as parasitaemia with or without symptoms, over a population at risk or person-time. Detected through passive or active surveillance. | |
| **Name** | N/A |
| **Definition/ assessment metric** | N/A |
| **Events** | N/A |
| **Total (or unit time)** | N/A |
| **Time of outcome assessment** | N/A |
| **Results**  (i.e., main results – epidemiological, unadjusted and adjusted, secondary outcomes, sensitivity analyses, subgroups, and clusters) | N/A |
| **Effect type** | N/A |
| **INCIDENCE OF SEVERE DISEASE**  Defined as hospitalization with parasitaemia, over a population at risk or person-time. | |
| **Name** | N/A |
| **Definition/ assessment metric** | N/A |
| **Events** | N/A |
| **Total (or unit time)** | N/A |
| **Time of outcome assessment** | N/A |
| **Results**  (i.e., main results – epidemiological, unadjusted and adjusted, secondary outcomes, sensitivity analyses, subgroups, and clusters) | N/A |
| **Effect type** | N/A |
| **PARASITE PREVALENCE**  Parasitaemia with or without symptoms, over the population sampled. Detected through cross-sectional surveys. | |
| **Name** | As above |
| **Definition/ assessment metric** | Presence of malaria parasites detected through cross-sectional surveys. Finger prick samples were collected for thick blood films. Children with fever within the last 24 hours were tested for malaria using a RDT. |
| **Events** | Survey 1  Standard LLIN – 851/1627 (52%)  PPF-LLIN - …  Survey 2  Standard LLIN – 1096/1761 (62%)  PPF-LLIN – 1124/1843 (61%)  Survey 3  Standard LLIN – 604 /1388 (44%)  PPF-LLIN – 757/1854 (41%)  Survey 4  Standard LLIN - …  PPF-LLIN – 2159/3758 )57%) |
| **Total (or unit time)** | Reported above |
| **Time of outcome assessment** | Survey 1 – June 2014  Survey 2 – December 2014  Survey 3 – May 2015  Survey 4 – July 2015 |
| **Results**  (i.e., main results – epidemiological, unadjusted and adjusted, secondary outcomes, sensitivity analyses, subgroups, and clusters) | At the second survey, the proportion of children with P falciparum infection was not significantly different between the groups (odds ratio [OR] 0·93, 95% CI 0·74 to 1·15).  The authors have not provided a report for the results for survey 3. |
| **Effect type** | OR/RR |
| **ALL-CAUSE MORTALITY**  Number of deaths over the population at risk or person-time. | |
| **Name** | As above |
| **Definition/ assessment metric** | As above |
| **Events** | Standard LLINs = 1  PPF-LLINs = 5 |
| **Total (or unit time)** | The authors have simply reported that of all 19 serious adverse events encountered six resulted in death. |
| **Time of outcome assessment** | Not reported |
| **Results**  (i.e., main results – epidemiological, unadjusted and adjusted, secondary outcomes, sensitivity analyses, subgroups, and clusters) | This can only be reported narratively.  The authors have not reported the months in which these AEs were encountered, and so we do not have an appropriate denominator to use for analysis |
| **Effect type** | Not synthesisable |
| **MALARIA MORTALITY**  Number of deaths attributed to malaria over the population at risk or person-time. | |
| **Name** | N/A |
| **Definition/ assessment metric** | N/A |
| **Events** | N/A |
| **Total (or unit time)** | N/A |
| **Time of outcome assessment** | N/A |
| **Results**  (i.e., main results – epidemiological, unadjusted and adjusted, secondary outcomes, sensitivity analyses, subgroups, and clusters) | N/A |
| **Effect type** | N/A |
| **PREVALENCE OF ANAEMIA**  Defined by study thresholds of anaemia. | |
| **Name** | As above |
| **Definition/ assessment metric** | Moderate anaemia was defined as haemoglobin concentration of <80g/L  Severe anaemia was defined as haemoglobin concentrations of <50g/L.  Finger prick samples were collected for haemoglobin measurement at each cross-sectional survey. This was performed using a portable spectrophotometer. |
| **Events** | Survey 1  Standard LLIN  Moderate – 104/1511 (7%)  Severe – 2/1511 (<1%)  PPF-LLIN  Moderate - …  Severe - …  Survey 2  Standard LLIN  Moderate – 113/1768 (6%)  Severe – 7/1768 (<1%)  PPF-LLIN  Moderate - 54/1782 (3%)  Severe - 0 (0%)  Survey 3  Standard LLIN  Moderate – 62/1420 (4%)  Severe – 1/1420 (<1%)  PPF-LLIN  Moderate – 51/1834 (3%)  Severe – 0 (0%)  Survey 4  Standard LLIN  Moderate -…  Severe - …  PPF-LLIN  Moderate – 62/3726 (2%)  Severe – 0 (0%) |
| **Total (or unit time)** | As above |
| **Time of outcome assessment** | Survey 1 – June 2014  Survey 2 – December 2014  Survey 3 – May 2015  Survey 4 – July 2015 |
| **Results**  (i.e., main results – epidemiological, unadjusted and adjusted, secondary outcomes, sensitivity analyses, subgroups, and clusters) | The prevalence of moderate anaemia was lower in the PPF-treated LLIN  group than in the standard LLIN group (OR 0·48, 95% CI 0·24–0·96; p=0·04; |
| **Effect type** | OR/RR |
| **COSTS** | |
| **Event/raw costs** | Not reported |
| **Results** | Not reported |
| **Time of cost assessment** | Not reported |
| **Resources needed/ used** | Not reported |
| **ADDITIONAL DATA** | |
| **Unintended benefits** | Not reported |
| **Harms** | There were 21 non-serious adverse events in the standard LLIN group and one in the PPF-treated LLIN group.  The PPF-LIN group was a case of bronchitis. The AEs in the standard LLIN-group included bronchitis, conjunctivitis, eye pruritus, pelvic pain, prurities, rhinitis, cough and watering eyes. All of which were resolved by study staff.  Severe AEs were also considered. There were 10 SAEs in the standard group and 9 in the PPF-LLIN group. These included severe malaria with other comorbidities, uncomplicated malaria with vomiting, gastroenteritis with severe dehydration and pneumonia. The others have provided the number of each that died (reported above) and were hospitalized (not reported above, as authors have not disambiguated between hospitalization due to malaria and other SAEs). |
| **Other contextual information present/measured/reported)** (feasibility, acceptability, preferences/values, impact on equity) | Not reported |
| **Entomological outcomes measured** (list) | Protocol 1: Knockdown and mortality rates, egg larvae production. geno and phenotype testing for resistance.  Protocol 2: Microscopy confirmed gametocyte carriers (GC), Entomological inoculation rate (EIR), exposure to mosquitos. |
| **Other** | The step-wedge design was adopted since it represents the type of deployment used by net distribution programmes. |
| **Source of funding** | EU Seventh Framework Programme.  Protocol 1 and 2: The study is supported by the European Union Seventh Framework Programme FP7/2007-2013 under grant agreement number 265660, AvecNet. We are grateful to the Sumitomo Chemical Company Limited who will donate the nets for this trial. |
| **Possible conflicts of interest** | The funder of the study and net manufacturers had no role in study design, data collection, data analysis, data interpretation, or writing of the report.  The authors have declared that they have no competing interests. |

| **Domain** | **Signalling question** | **Response** | **Comments** |
| --- | --- | --- | --- |
| **Bias arising from the randomization process** | 1a.1 Was the allocation sequence random? | Y | Random selection was done with Stata version 10. five randomly selected clusters of villages were provided with PPF-treated LLINs and the remaining 35 village clusters with standard LLINs.  No information provided regarding allocation concealment |
|  | 1a.2 Was the allocation sequence concealed until clusters were enrolled and assigned to interventions? | NI |  |
|  | 1a.3 Did baseline differences between intervention groups suggest a problem with the randomization process? | N | This was a stepped-wedged trial |
|  | **Risk of bias judgement** | **Some concerns** | Random selection was done with Stata version 10. five randomly selected clusters of villages were provided with PPF-treated LLINs and the remaining 35 village clusters with standard LLINs.  No information provided regarding allocation concealment  This was a stepped-wedged trial |
| **Bias arising from the timing of identification or recruitment of participants** | 1b.1 Were all the individual participants identified and recruited (if appropriate) before randomization of clusters? | **Y** | Pariticpants belonged to households within villages which were then organised into clusters |
|  | 1b.2 If N/PN/NI to 1b.1: Is it likely that selection of individual participants was affected by knowledge of the intervention assigned to the cluster? |  |  |
|  | 1b.3 Were there baseline imbalances that suggest differential identification or recruitment of individual participants between intervention groups? | **N** |  |
|  | **Risk of bias judgement** | **Low** | Low |
| **Bias due to deviations from intended interventions** | 2.1a Were participants aware that they were in a trial? | Y | All participants had to provide informed consent to recieve the intervention or control |
|  | 2.1b If Y/PY/NI to 2.1a: Were participants aware of their assigned intervention during the trial? | N | both types of nets were of similar shape, size, and colour, and blood films were read by microscopists masked to the identity and intervention status of the participants |
|  | 2.2 Were carers and people delivering the interventions aware of participants' assigned intervention during the trial? | N |  |
|  | 2.3 If Y/PY/NI to 2.1b or 2.2: Were there deviations from the intended intervention that arose because of the trial context? | NA |  |
|  | 2.4 If Y/PY to 2.3: Were these deviations likely to have affected the outcome? | NA |  |
|  | 2.5 If Y/PY/NI to 2.4: Were these deviations from intended intervention balanced between groups? | NA |  |
|  | 2.6 Was an appropriate analysis used to estimate the effect of assignment to intervention? | Y | Yes ITT was followed |
|  | 2.7 If N/PN/NI to 2.6: Was there potential for a substantial impact (on the result) of the failure to analyse participants in the group to which they were randomized ? | NA |  |
|  | **Risk of bias judgement** | **Low** | All participants had to provide informed consent to recieve the intervention or control  both types of nets were of similar shape, size, and colour, and blood films were read by microscopists masked to the identity and intervention status of the participants. |
| **Outcome: Malaria Case Incidence** | | | |
| **Bias due to missing outcome data** | 3.1a Were data for this outcome available for all clusters that recruited participants? | Y |  |
|  | 3.1b Were data for this outcome available for all, or nearly all, participants within clusters? | N | Only children aged 6 months to 5 years provided outcome data if they presented to a health facility with malaria symptoms |
|  | 3.2 If N/PN/NI to 3.1a or 3.1b: Is there evidence that the result was not biased by missing data? | Y | This is an appropriate method for assessing this outcome (passive case detection) |
|  | 3.3 If N/PN to 3.2 Could missingness in the outcome depend on its true value? | NA |  |
|  | 3.4 If Y/PY/NI to 3.3: Is it likely that missingness in the outcome depended on its true value? | NA |  |
|  | **Risk of bias judgement** | **Low** | Only children aged 6 months to 5 years provided outcome data if they presented to a health facility with malaria symptoms  This is an appropriate method for assessing this outcome (passive case detection) |
| **Bias in measurement of the outcome** | 4.1 Was the method of measuring the outcome inappropriate? | N | Incidence of clinical episodes of malaria among cohort children presenting at health facilities. Confirmed through presence of a fever and RDT. |
|  | 4.2 Could measurement or ascertainment of the outcome have differed between intervention groups? | N |  |
|  | 4.3a If N/PN/NI to 4.1 and 4.2: Were outcome assessors aware that a trial was taking place? | Y |  |
|  | 4.3b If Y/PY/NI to 4.3a: Were outcome assessors aware of the intervention received by study participants? | N | blood films were read by microscopists masked to the identity and intervention status of the participants |
|  | 4.4 If Y/PY/NI to 4.3b: Could assessment of the outcome have been influenced by knowledge of intervention received? | NA |  |
|  | 4.5 If Y/PY/NI to 4.4: Is it likely that assessment of the outcome was influenced by knowledge of intervention received? | NA |  |
|  | **Risk of bias judgement** | **Low** |  |
| **Bias in selection of the reported result** | 5.1 Were the data that produced this result analysed in accordance with a pre-specified analysis plan that was finalized before unblinded outcome data were available for analysis? | Y | A protocol (2) was provided and followed and there has been full and complete reporting of all outcomes specified. |
|  | 5.2 ... multiple eligible outcome measurements (e.g. scales, definitions, time points) within the outcome domain? | N |  |
|  | 5.3 ... multiple eligible analyses of the data? | N |  |
|  | **Risk of bias judgement** | **Low** |  |
| **Outcome: Parasite Prevalence** | | | |
| **Bias due to missing outcome data** | 3.1a Were data for this outcome available for all clusters that recruited participants? | Y |  |
|  | 3.1b Were data for this outcome available for all, or nearly all, participants within clusters? | N | Children were randomly selected for enrolment in the cross-sectional survey |
|  | 3.2 If N/PN/NI to 3.1a or 3.1b: Is there evidence that the result was not biased by missing data? | Y | Random selection suggests that the result will not be biased |
|  | 3.3 If N/PN to 3.2 Could missingness in the outcome depend on its true value? | NA |  |
|  | 3.4 If Y/PY/NI to 3.3: Is it likely that missingness in the outcome depended on its true value? | NA |  |
|  | **Risk of bias judgement** | **Low** | Children were randomly selected for enrolment in the cross-sectional survey  Random selection suggests that the result will not be biased |
| **Bias in measurement of the outcome** | 4.1 Was the method of measuring the outcome inappropriate? | N | Finger prick samples were collected for thick blood films. Children with fever within the last 24 hours were tested for malaria using an RDT. |
|  | 4.2 Could measurement or ascertainment of the outcome have differed between intervention groups? | N |  |
|  | 4.3a If N/PN/NI to 4.1 and 4.2: Were outcome assessors aware that a trial was taking place? | Y |  |
|  | 4.3b If Y/PY/NI to 4.3a: Were outcome assessors aware of the intervention received by study participants? | N | Blood films were read by microscopists masked to the identity and intervention status of the participants |
|  | 4.4 If Y/PY/NI to 4.3b: Could assessment of the outcome have been influenced by knowledge of intervention received? | NA |  |
|  | 4.5 If Y/PY/NI to 4.4: Is it likely that assessment of the outcome was influenced by knowledge of intervention received? | NA |  |
|  | **Risk of bias judgement** | **Low** |  |
| **Bias in selection of the reported result** | 5.1 Were the data that produced this result analysed in accordance with a pre-specified analysis plan that was finalized before unblinded outcome data were available for analysis? | Y | A protocol (2) was provided and followed and there has been full and complete reporting of all outcomes specified. |
|  | 5.2 ... multiple eligible outcome measurements (e.g. scales, definitions, time points) within the outcome domain? | N | Survey data was reported at 4 different time points, however the results of each time point have been reported in full |
|  | 5.3 ... multiple eligible analyses of the data? | N |  |
|  | **Risk of bias judgement** | **Low** | A protocol (2) was provided and followed and there has been full and complete reporting of all outcomes specified.  Survey data was reported at 4 different time points, however the results of each time point have been reported in full |
| **Outcome: Prevalence of Anaemia** | | | |
| **Bias due to missing outcome data** | 3.1a Were data for this outcome available for all clusters that recruited participants? | Y |  |
|  | 3.1b Were data for this outcome available for all, or nearly all, participants within clusters? | Y |  |
|  | 3.2 If N/PN/NI to 3.1a or 3.1b: Is there evidence that the result was not biased by missing data? | NA |  |
|  | 3.3 If N/PN to 3.2 Could missingness in the outcome depend on its true value? | NA |  |
|  | 3.4 If Y/PY/NI to 3.3: Is it likely that missingness in the outcome depended on its true value? | NA |  |
|  | **Risk of bias judement** | **Low** |  |
| **Bias in measurement of the outcome** | 4.1 Was the method of measuring the outcome inappropriate? | N |  |
|  | 4.2 Could measurement or ascertainment of the outcome have differed between intervention groups? | N |  |
|  | 4.3a If N/PN/NI to 4.1 and 4.2: Were outcome assessors aware that a trial was taking place? | Y |  |
|  | 4.3b If Y/PY/NI to 4.3a: Were outcome assessors aware of the intervention received by study participants? | Y |  |
|  | 4.4 If Y/PY/NI to 4.3b: Could assessment of the outcome have been influenced by knowledge of intervention received? | N |  |
|  | 4.5 If Y/PY/NI to 4.4: Is it likely that assessment of the outcome was influenced by knowledge of intervention received? | NA |  |
|  | **Risk of bias judgement** | **Low** |  |
| **Bias in selection of the reported result** | 5.1 Were the data that produced this result analysed in accordance with a pre-specified analysis plan that was finalized before unblinded outcome data were available for analysis? | Y | A protocol (2) was provided and followed and there has been full and complete reporting of all outcomes specified. |
|  | 5.2 ... multiple eligible outcome measurements (e.g. scales, definitions, time points) within the outcome domain? | N |  |
|  | 5.3 ... multiple eligible analyses of the data? | N |  |
|  | **Risk of bias judgement** | **Low** | A protocol (2) was provided and followed and there has been full and complete reporting of all outcomes specified. |

# Supporting information 4 – Additional conducted analyses

Supplementary Material to New Nets Analyses and SoF’s.

All decisions that were made by the methods team to facilitate meta-analysis have been recorded here. All data have been rounded to the nearest two decimal places where required.

Accrombessi 2021 –

Intervention 1 – Interceptor G2 (chlorfenapyr (4.8 g/kg) and alpha-cypermethrin (2.4 g/kg))

Intervention 2 -Royal Guard (combination of pyriproxyfen (5.5g/kg) and a-cypermethrin (5.5g/kg)

Comparator - Interceptor LLIN (pyrethroid a-cypermethrin 5g/kg)

Mosha 2022 –

Intervention 1 -Royal Guard (combination of pyriproxyfen (5.5g/kg) and a-cypermethrin (5.5g/kg)

Intervention 2 - Interceptor G2 (chlorfenapyr (4.8 g/kg) and alpha-cypermethrin (2.4 g/kg))

Comparator 1 - Interceptor LLIN (pyrethroid a-cypermethrin 5g/kg)

Comparator 2 - Olyset Plus (combining piperonyl butoxide (10 g/kg) and the pyrethroid permethrin (20 g/kg))

Tiono 2018 –

Intervention - Sumitomo Chemical PPF-LLIN (2% w/w permethrin and 1% w/w pyriproxyfen)

Comparator - Sumitomo Chemical Standard LLIN ( 2% w/w permethrin)

**Analysis 1 – Chlorfenapyr-pyrethroid nets versus Pyrethroid-only nets**

**1.8 Parasite Prevalence (furthest possible follow-up)**


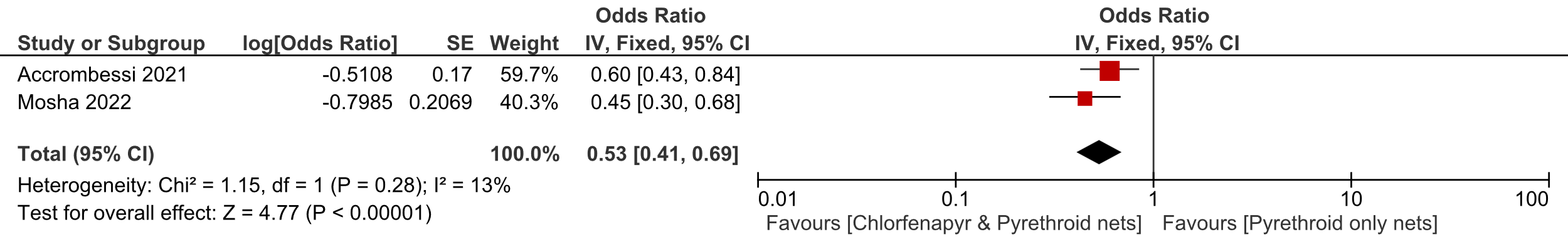


Accrombessi 2021

Have reported the data for 6 months post intervention and 18 months post intervention. The data for 18 months post-intervention has been entered into RevMan as reported.

Mosha 2022

Have reported the data for 12 months, 18 months and 24 months post intervention. The data for 24 months post-intervention has been entered into RevMan as reported.

**1.9 Prevalence of Anaemia (6-months follow-up)**

**
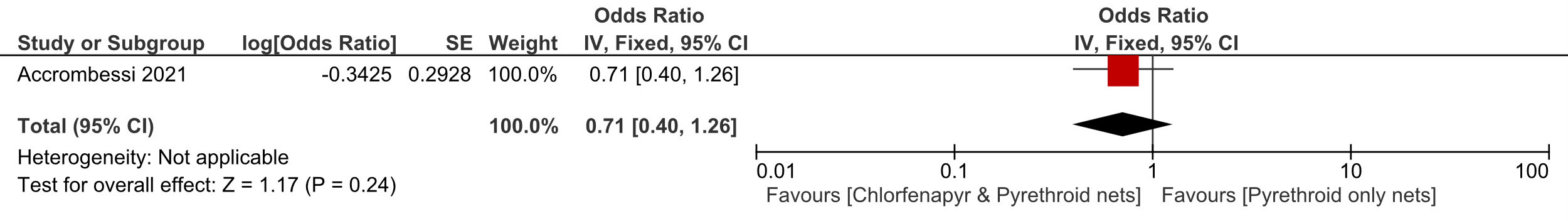
**

**1.10 Prevalence of Anaemia (12-months follow-up)**


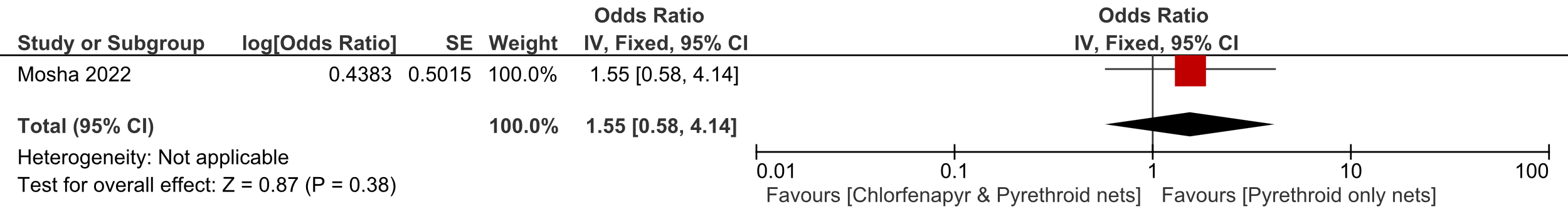


**1.11 Prevalence of Anaemia (18-months follow-up)**

**
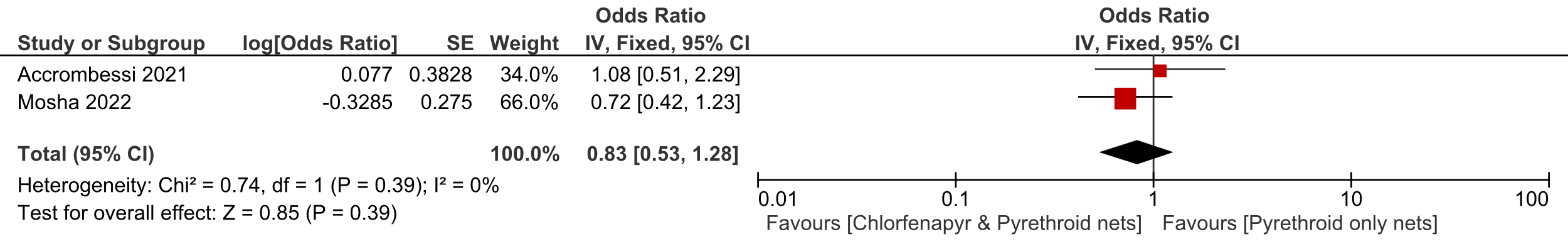
**

**1.12 Prevalence of Anaemia (24-months follow-up)**

**
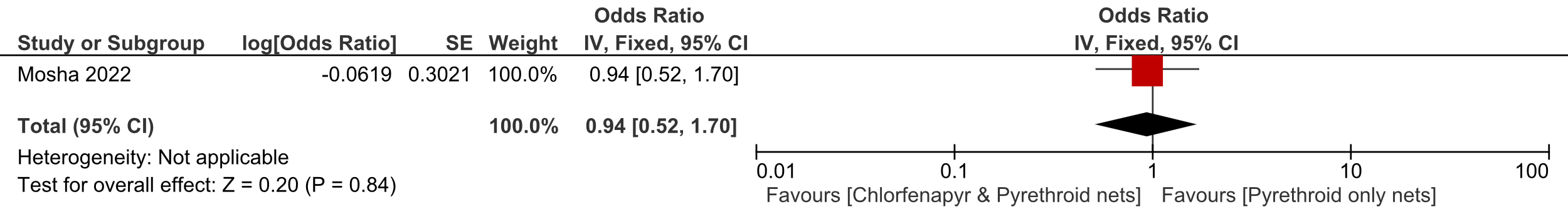
**

**1.13 Prevalence of Anaemia (furthest possible follow-up)**

**
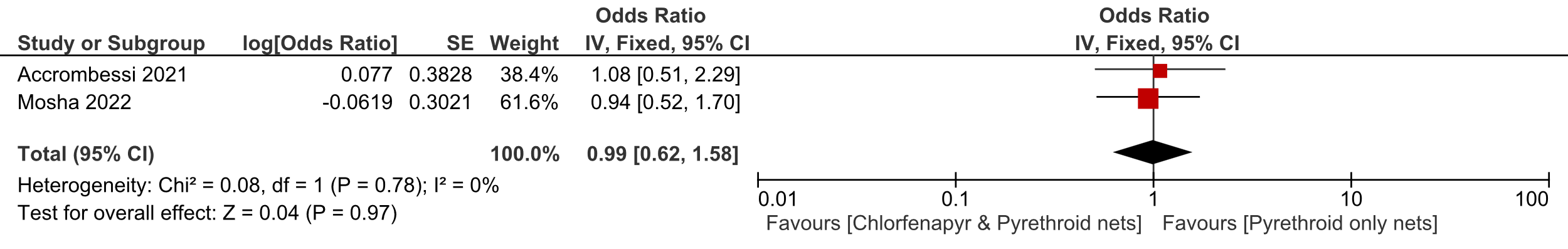
**

Accrombessi 2021

Have reported the data for 6 months post intervention and 18 months post intervention. The data for 18 months post-intervention has been entered into RevMan as reported.

Mosha 2022

Have reported the number of anaemic children (Hb concentrations) over total number surveyed. Have reported the data for 12 months, 18 months and 24 months post intervention. The data for 24 months post-intervention has been entered into RevMan as reported.

**Analysis 2 – Chlorfenapyr-pyrethroid nets dual AI nets versus PBO dual AI nets**

**2.7 Prevalence of Anaemia (12-months follow-up)**

**
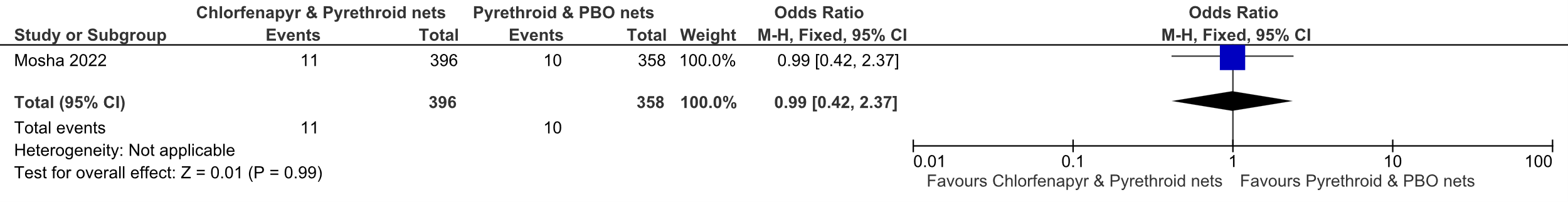
**

**2.8 Prevalence of Anaemia (18-months follow-up)**

**
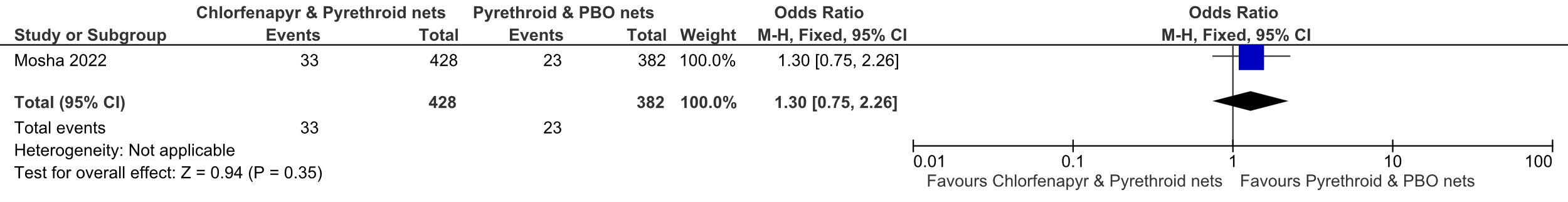
**

**2.9 Prevalence of Anaemia (24-months follow-up)**

**
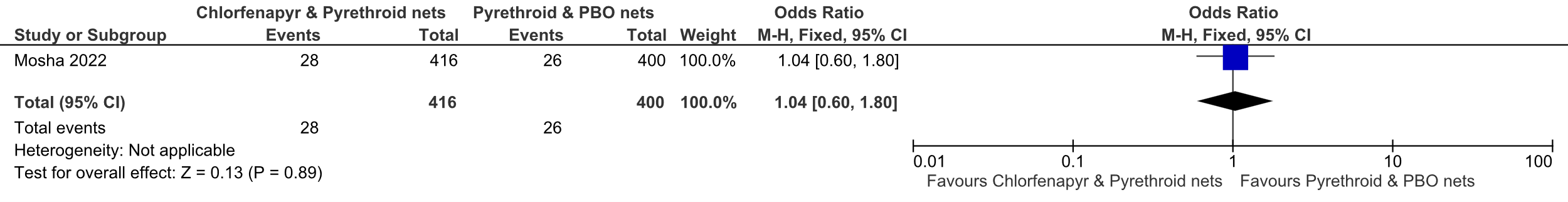
**

**Analysis 3 – Pyriproxyfen-pyrethroid nets versus Pyrethroid-only nets**

**3.8 Parasite Prevalence (furthest possible follow-up)**


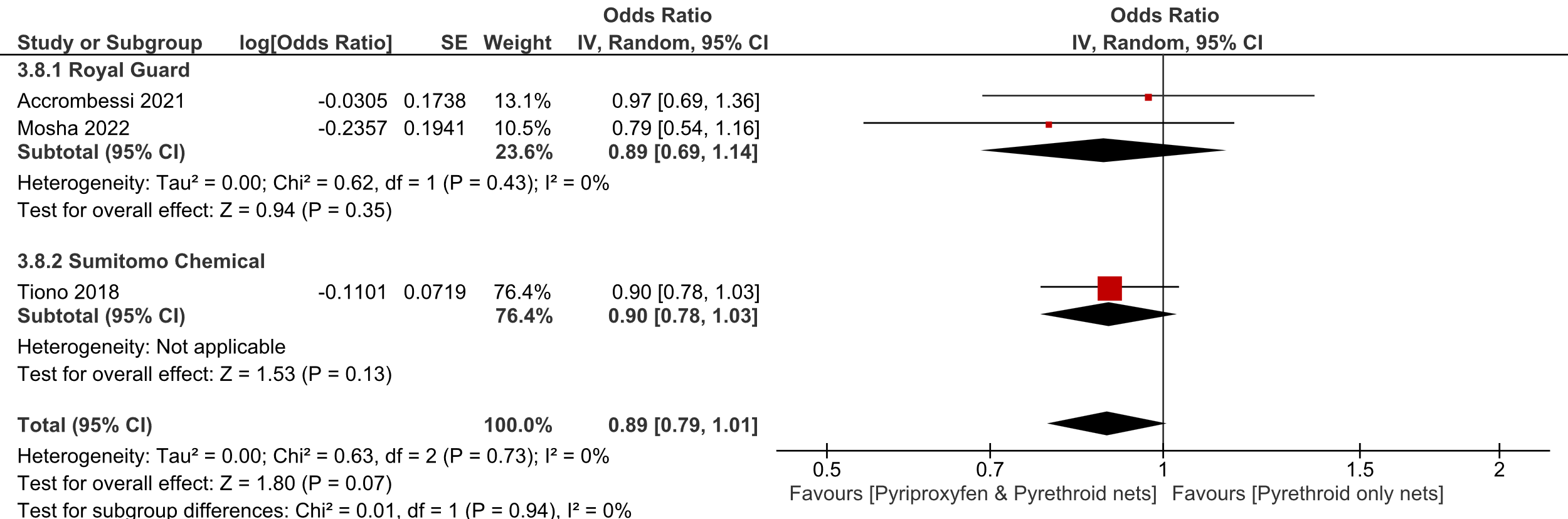


Accrombessi 2021

Have reported the data for 6 months post intervention and 18 months post intervention. The data for 18 months post-intervention has been entered into RevMan as reported.

Mosha 2022

Have reported the data for 12 months, 18 months and 24 months post intervention. The data for 24 months post-intervention has been entered into RevMan as reported.

Tiono 2018

Have reported their data from surveys conducted at 4 different time points. As this was a stepped wedge trial, surveys 2 (December 2014) and 3 (May 2015) represent the time points when intervention control ratio was 50:50. Have reported the data from survey 3 into RevMan as reported. This was chosen as this was the longest time point post-intervention, where the intervention ratio is 50:50.

ICEMAN Credibility Assessments

Very Low Credibility. Very likely no effect modification. Use overall effect for each subgroup.

**3.9 Prevalence of Anaemia (6-months follow-up)**

**
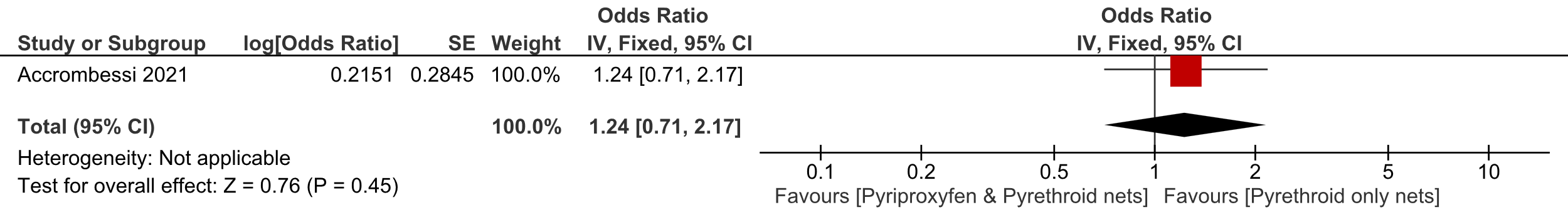
**

**3.10 Prevalence of Anaemia (12-months follow-up)**

**
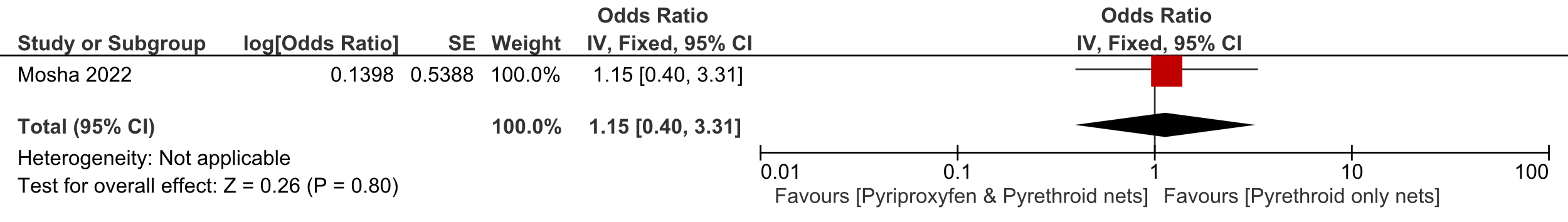
**

**3.11 Prevalence of Anaemia (18-months follow-up)**

**
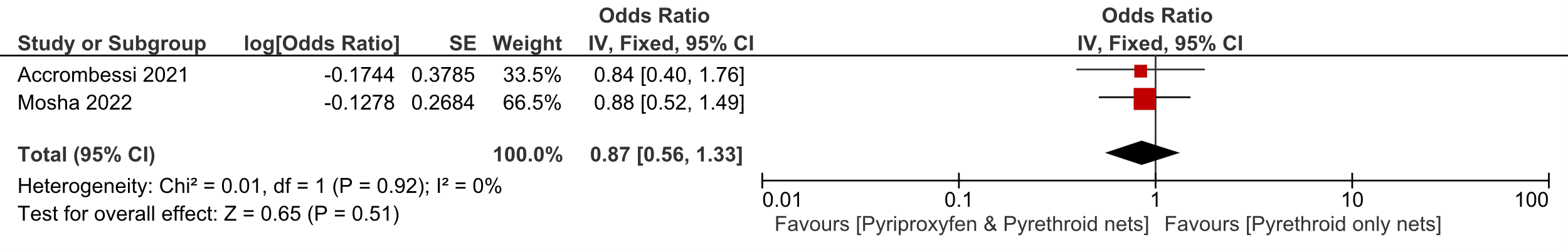
**

**3.12 Prevalence of Anaemia (24-months follow-up)**

**
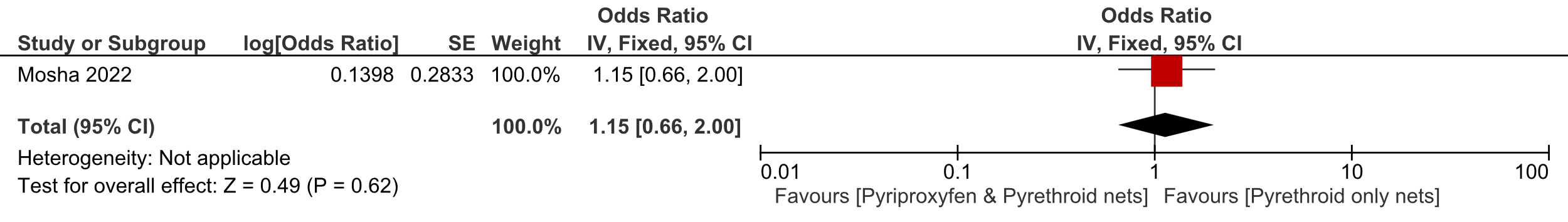
**

**3.13 Prevalence of Anaemia (furthest possible follow-up)**

**
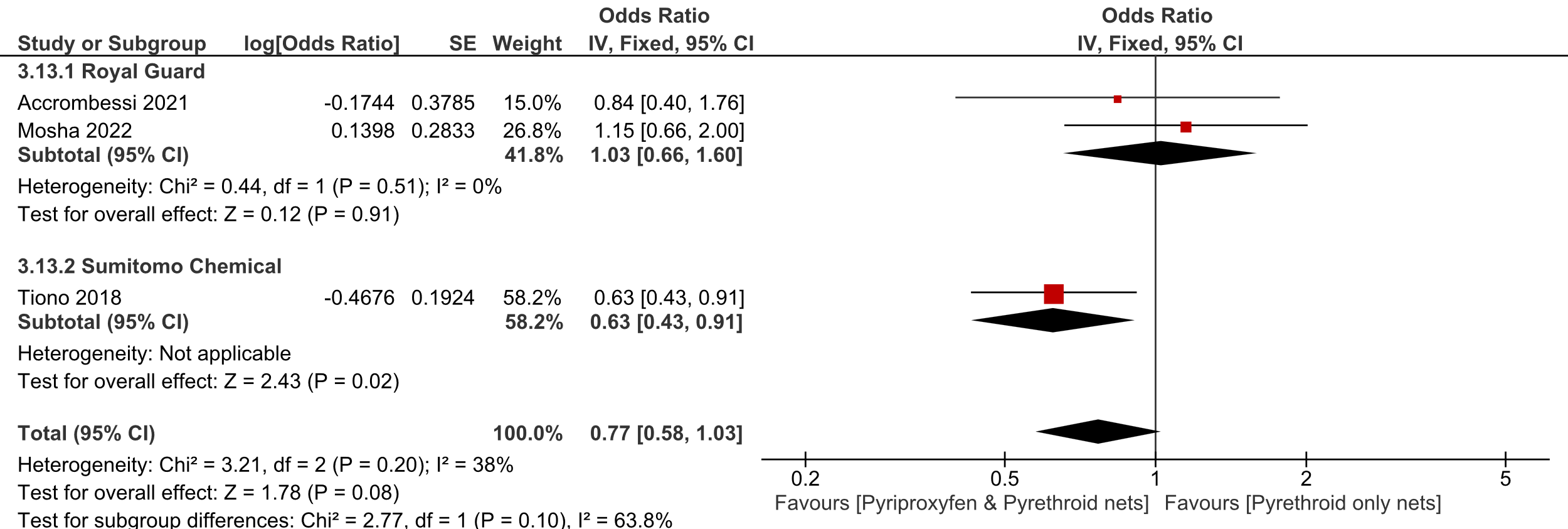
**

Accrombessi 2021

Have reported their data from at 6-, and 18-months post-intervention. The data for 18 months post-intervention has been entered into RevMan as reported.

Mosha 2022

Have reported the data for 12 months, 18 months and 24 months post intervention. The data for 24 months post-intervention has been entered into RevMan as reported.

Tiono 2018

Have reported their data from surveys conducted at 4 different time points. As this was a stepped wedge trial, surveys 2 (December 2014) and 3 (May 2015) represent the time points when intervention control ratio was 50:50. Have reported the data from survey 3 into RevMan as reported. This was chosen as this was the longest time point post-intervention.

ICEMAN Credibility Assessments

Low Credibility. Likely no effect modification. Use overall effect for each subgroup, but note remaining uncertainty.

**Analysis 4 – Pyriproxyfen-pyrethroid dual AI nets versus PBO dual AI nets**

**4.7 Prevalence of Anaemia (12-months follow-up)**

**
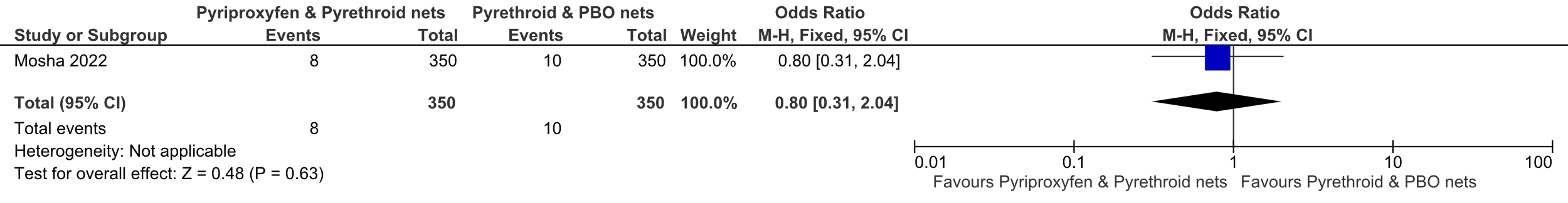
**

**4.8 Prevalence of Anaemia (18-months follow-up)**

**
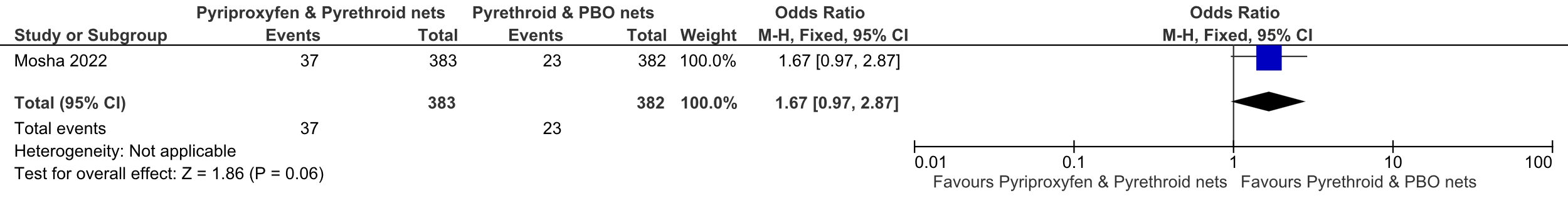
**

**4.9 Prevalence of Anaemia (24-months follow-up)**

**
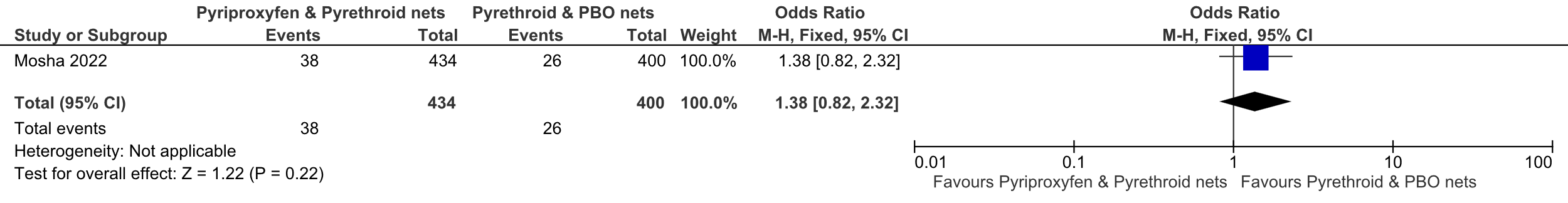
**

**Subgroup analyses (With ICEMAN Assessments)**

**Vector Species (Funestus or Gambiae/Coluzzi)**

**Analysis 5 – Chlorfenapyr-pyrethroid nets versus Pyrethroid-only nets**

**5.1 Malaria case incidence**

**
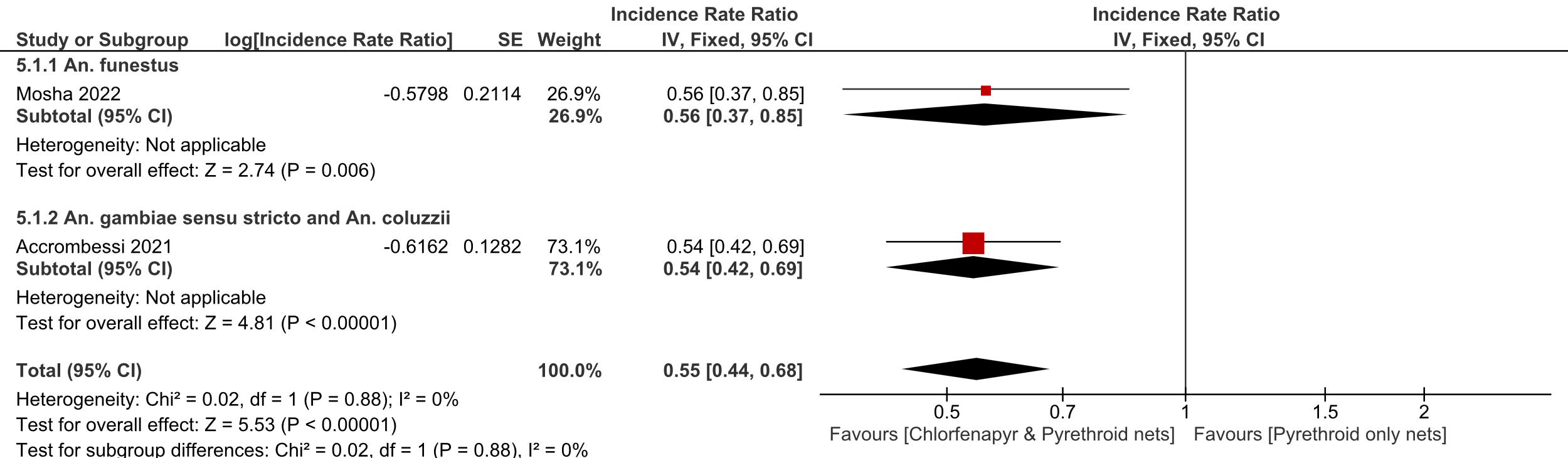
**

ICEMAN Credibility Assessments

Low Credibility. Likely no effect modification. Use overall effect for each subgroup, but note remaining uncertainty

**5.2 Malaria case incidence (1-year post)**

**
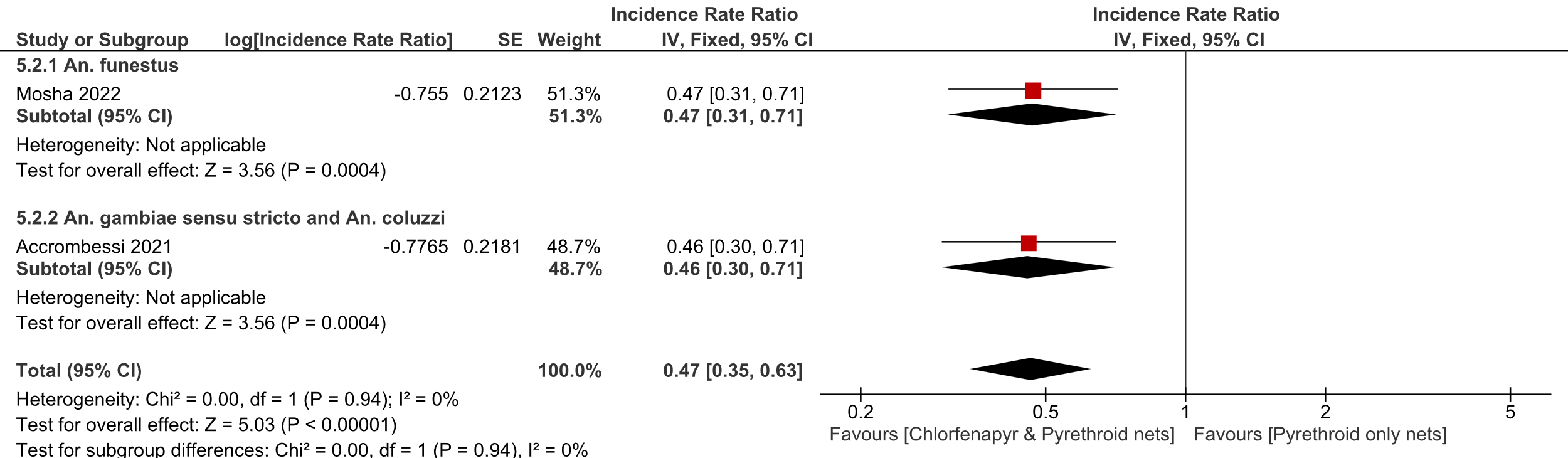
**

ICEMAN Credibility Assessments

Very low Credibility. Very likely no effect modification. Use overall effect for each subgroup.

**5.3 Malaria case incidence (2-year post)**

**
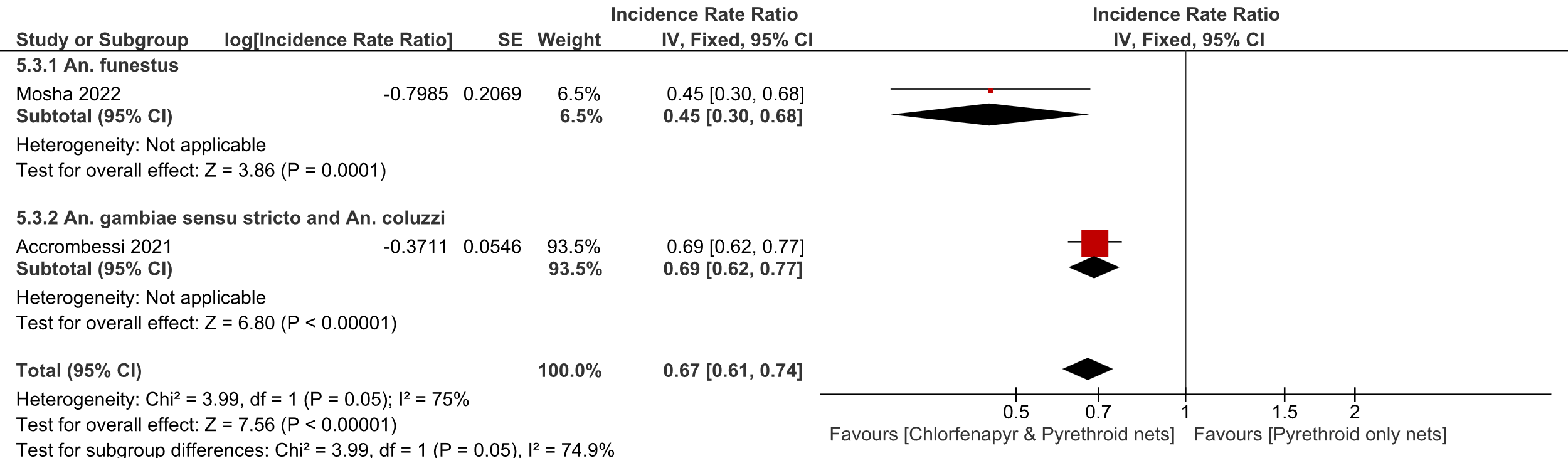
**

ICEMAN Credibility Assessments

Very low Credibility. Very likely no effect modification. Use overall effect for each subgroup.

**5.4 Parasite prevalence (18-months follow-up)**

**
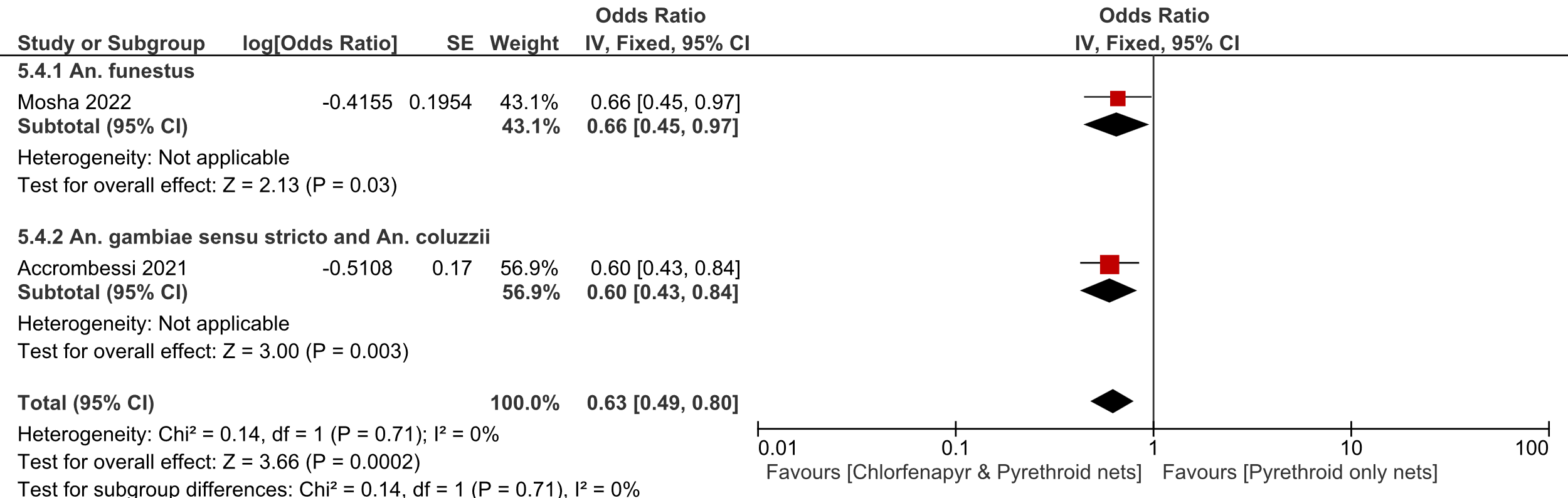
**

ICEMAN Credibility Assessments

Very Low Credibility. Very likely no effect modification. Use overall effect for each subgroup.

**5.5 Parasite prevalence (furthest possible follow-up)**

**
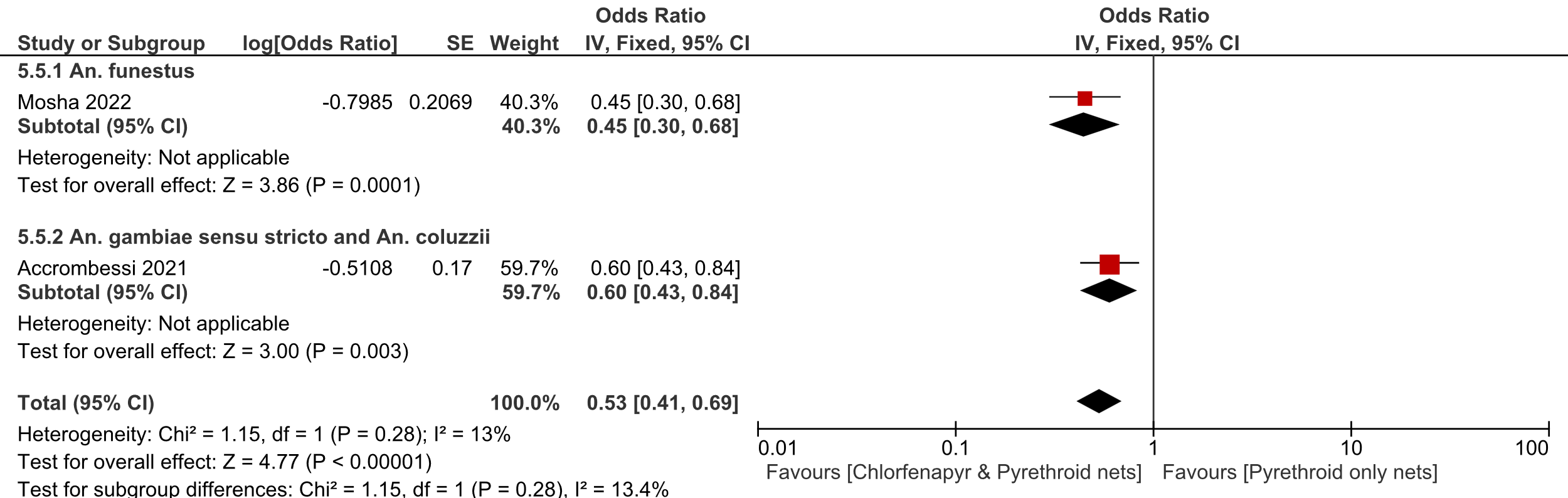
**

ICEMAN Credibility Assessments

Low Credibility. Likely no effect modification. Use overall effect for each subgroup, but note remaining uncertainty

**5.6 Prevalence of anaemia (18-months follow-up)**

**
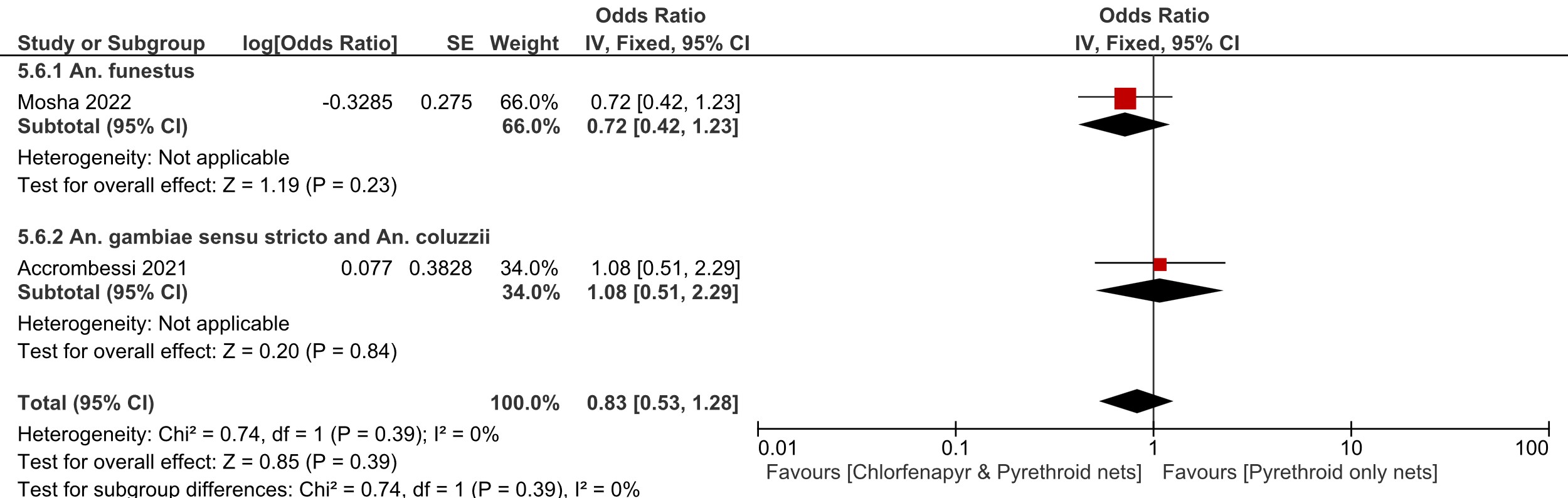
**

ICEMAN Credibility Assessments

Very Low Credibility. Very likely no effect modification. Use overall effect for each subgroup.

**5.7 Prevalence of anaemia (furthest possible follow-up)**

**
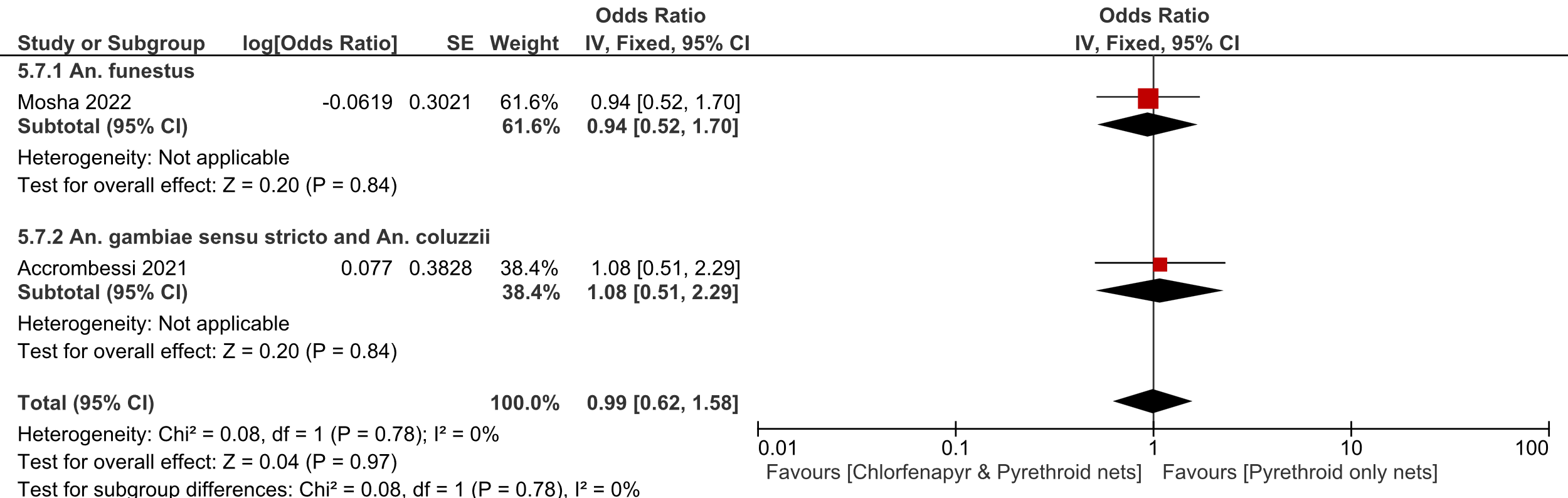
**

ICEMAN Credibility Assessments

Very Low Credibility. Very likely no effect modification. Use overall effect for each subgroup.

**Analysis 6 – Chlorfenapyr-pyrethroid nets versus Pyrethroid-onlynets**

**Setting (Rural or Mixed)**

**6.1 Malaria case incidence (overall)**

**
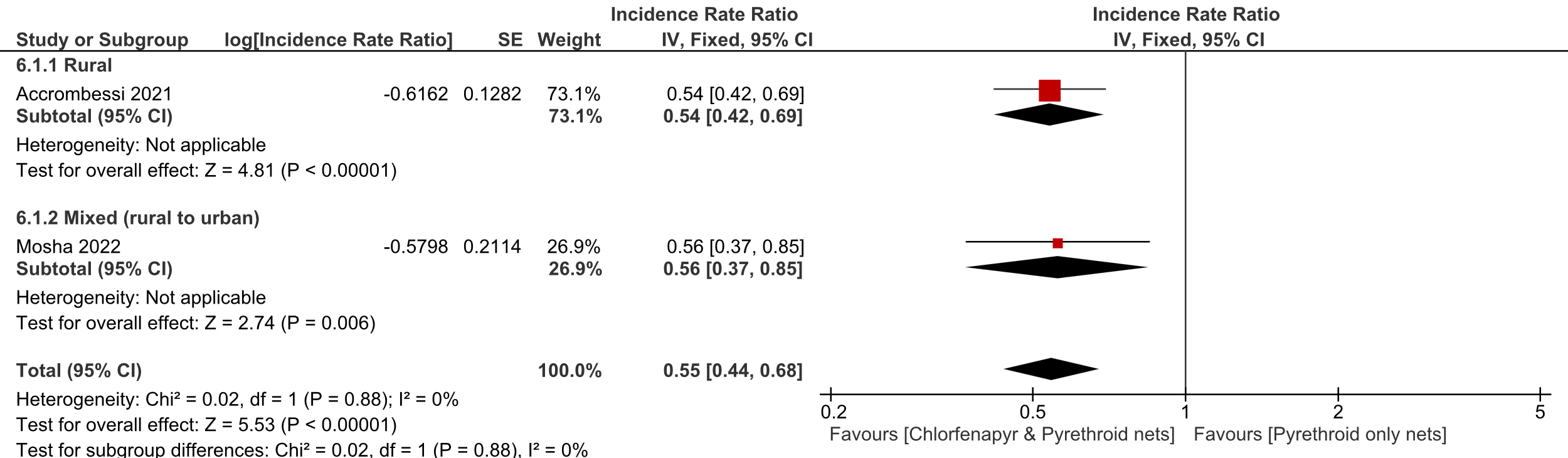
**

ICEMAN Credibility Assessments

Low Credibility. Likely no effect modification. Use overall effect for each subgroup, but note remaining uncertainty

**6.2 Malaria case incidence (1-year post)**

**
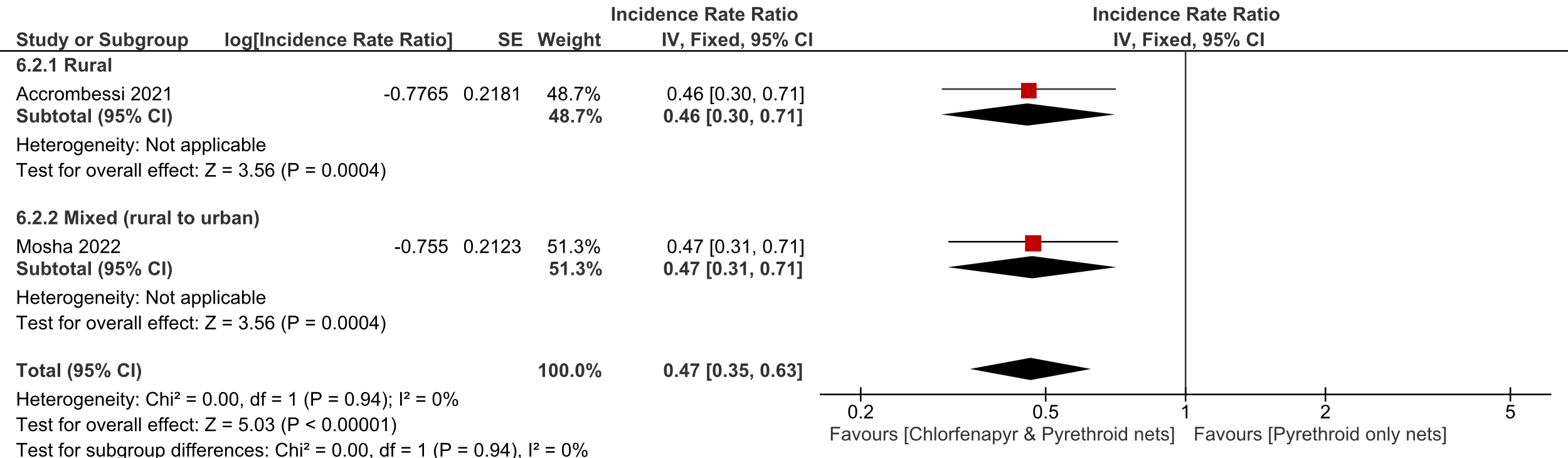
**

ICEMAN Credibility Assessments

Very low Credibility. Very likely no effect modification. Use overall effect for each subgroup.

**6.3 Malaria case incidence (2-year post)**

**
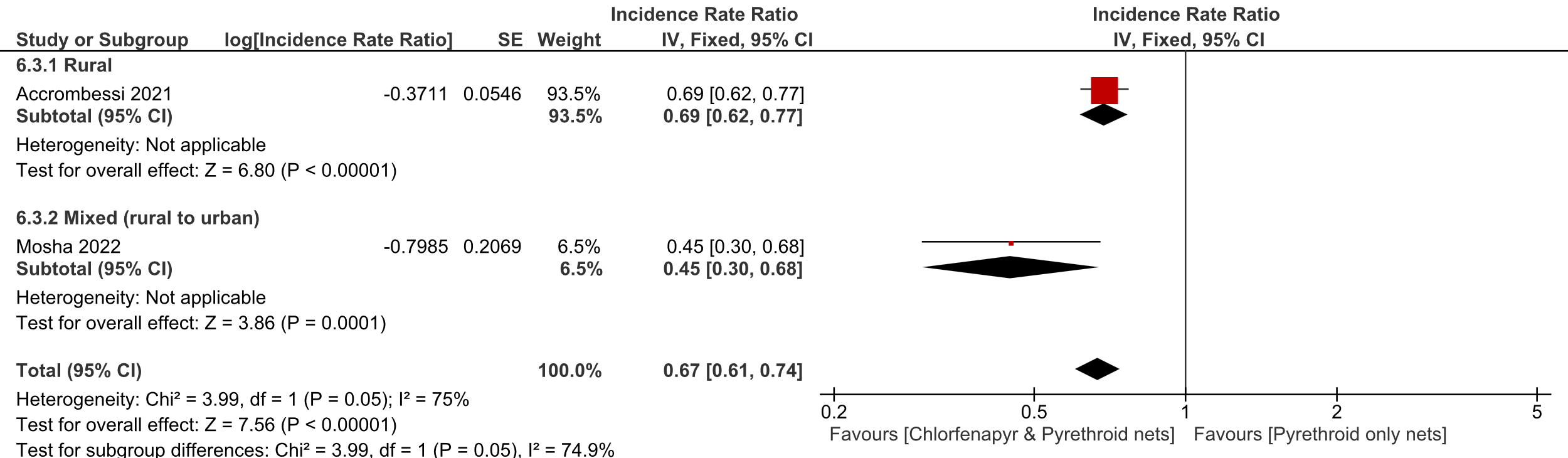
**

ICEMAN Credibility Assessments

Very low Credibility. Very likely no effect modification. Use overall effect for each subgroup.

**6.4 Parasite prevalence (18-months follow-up)**

**
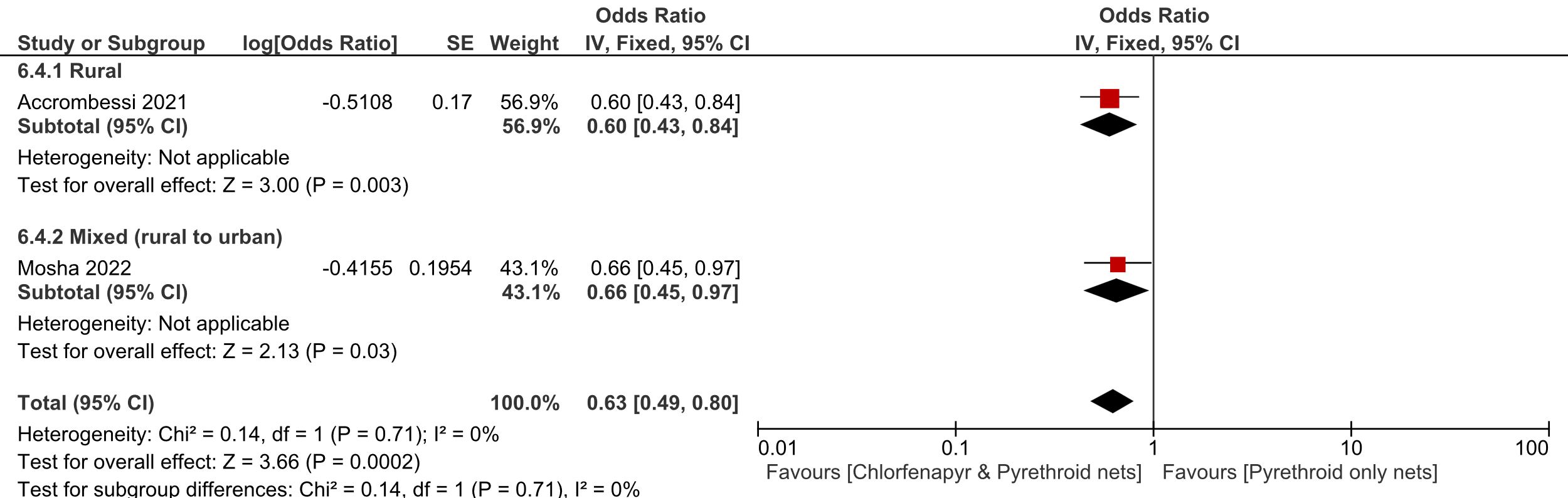
**

ICEMAN Credibility Assessments

Very Low Credibility. Very likely no effect modification. Use overall effect for each subgroup.

**6.5 Parasite prevalence (furthest possible follow-up)**

**
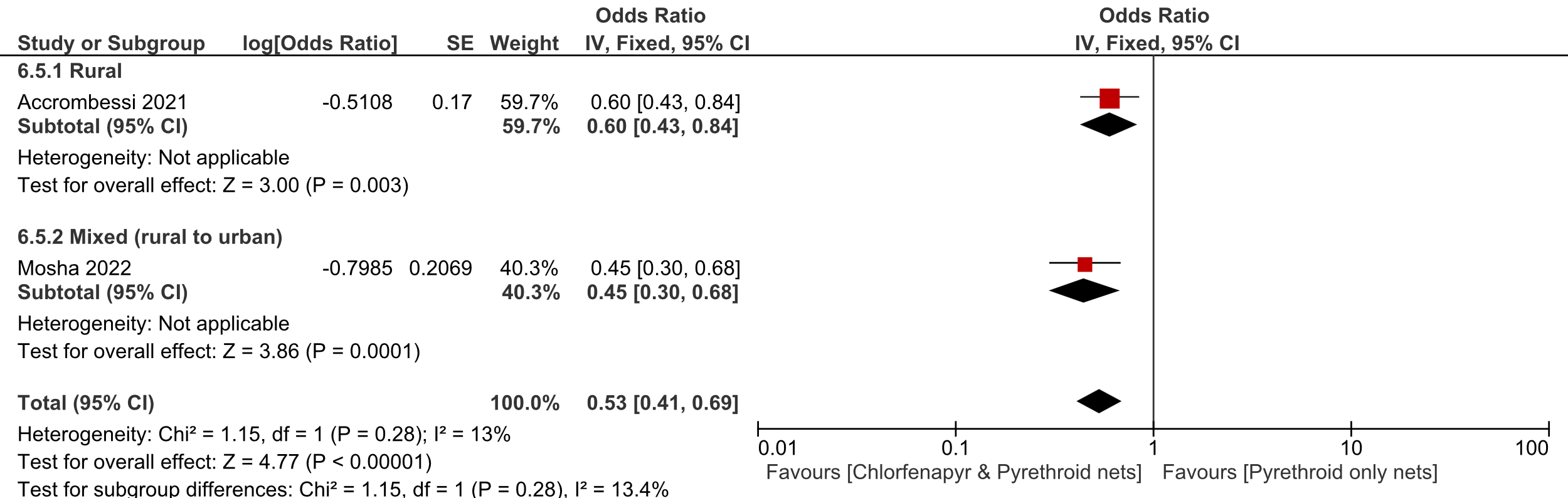
**

ICEMAN Credibility Assessments

Low Credibility. Likely no effect modification. Use overall effect for each subgroup, but note remaining uncertainty

**6.6 Prevalence of anaemia (18-months follow-up)**

**
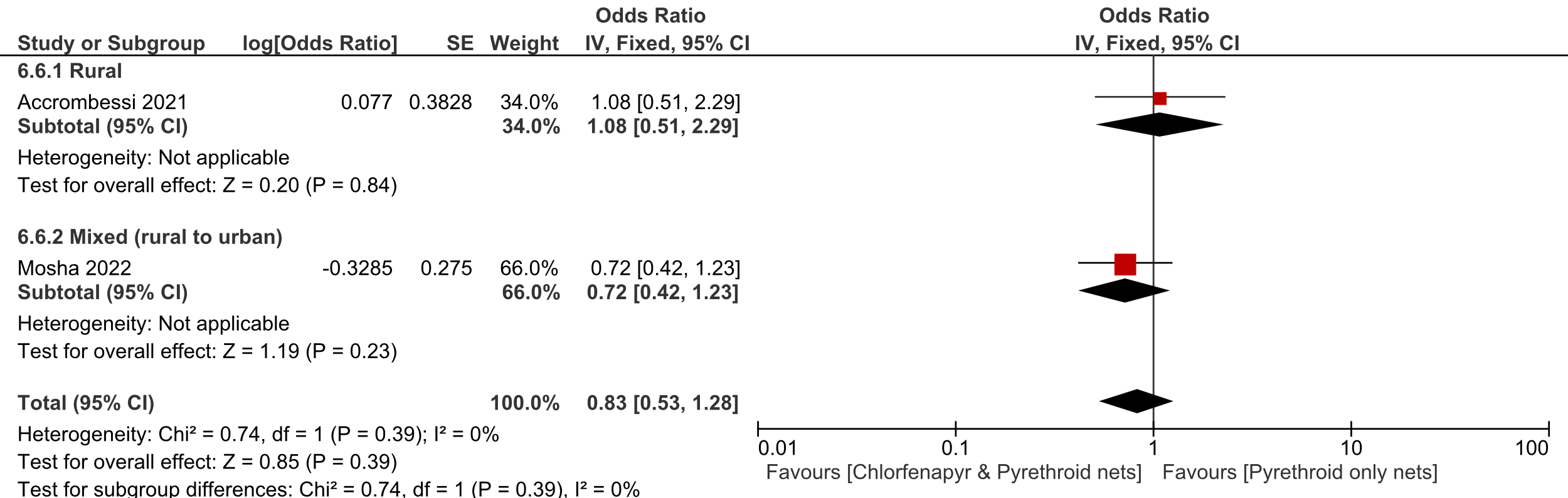
**

ICEMAN Credibility Assessments

Very Low Credibility. Very likely no effect modification. Use overall effect for each subgroup.

**6.7 Prevalence of anaemia (furthest possible follow-up)**

**
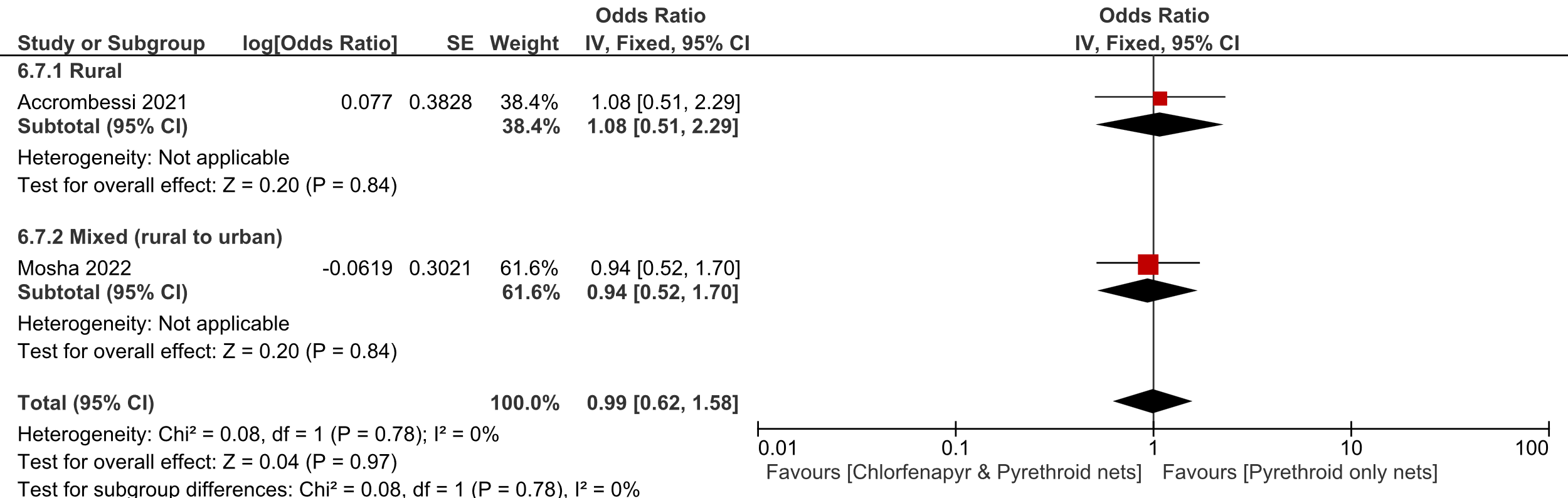
**

ICEMAN Credibility Assessments

Very Low Credibility. Very likely no effect modification. Use overall effect for each subgroup.

**Vector Species (Funestus or Gambiae/Coluzzi)**

**Analysis 7 – Pyriproxyfen-pyrethroid nets versus Pyrethroid-only nets**

**7.1 Malaria case incidence (overall)**

**
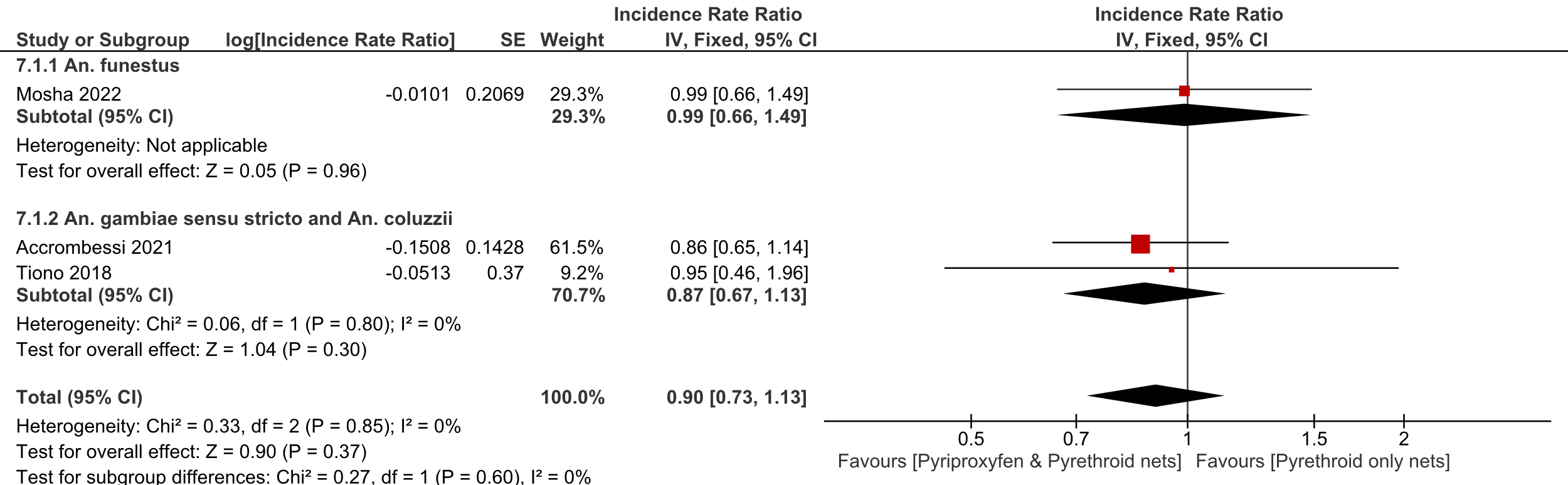
**

ICEMAN Credibility Assessments

Very low Credibility. Very likely no effect modification. Use overall effect for each subgroup.

**7.2 Malaria case incidence (1-year post intervention)**

**
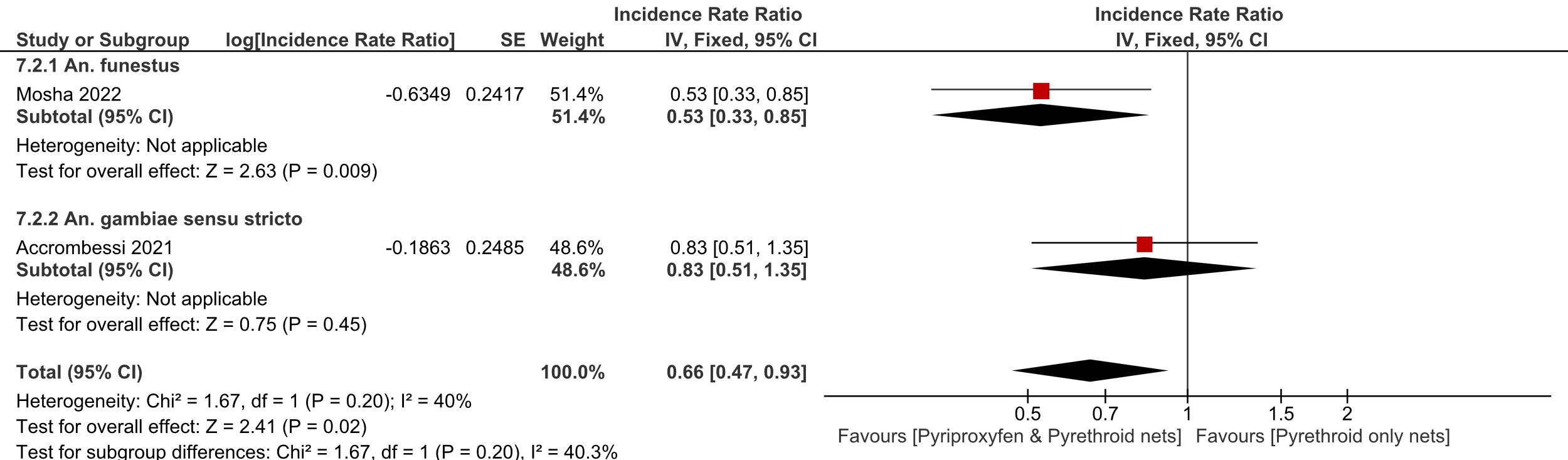
**

ICEMAN Credibility Assessments

Very low Credibility. Likely no effect modification. Use overall effect for each subgroup, but note remaining uncertainty

**7.3 Malaria case incidence (2-year post intervention)**

**
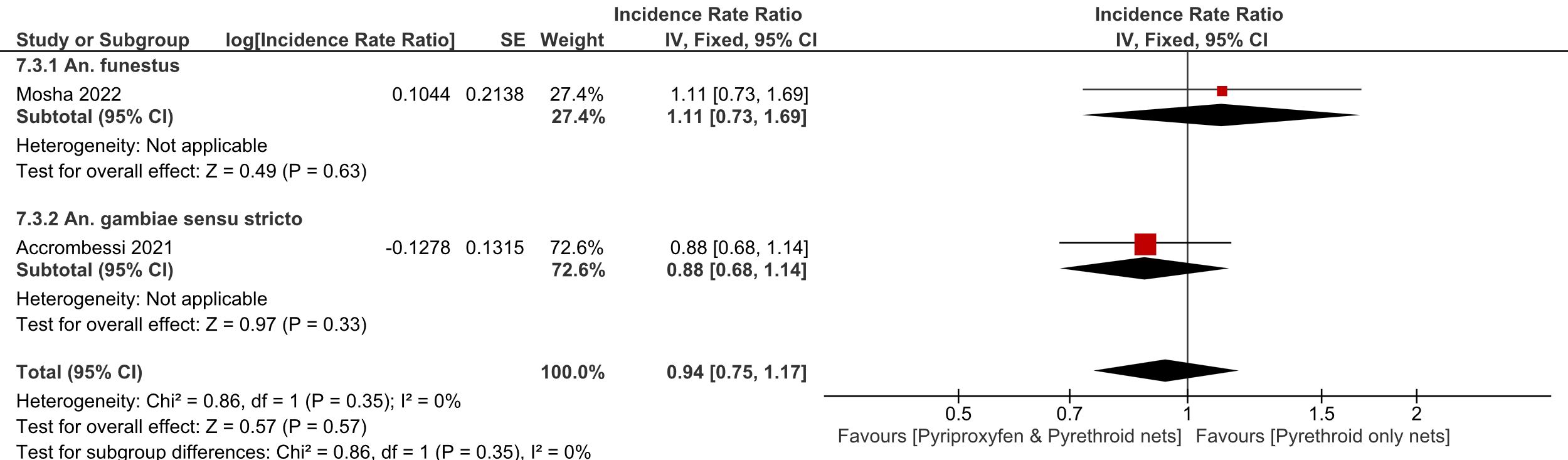
**

ICEMAN Credibility Assessments

Very low Credibility. Likely no effect modification. Use overall effect for each subgroup, but note remaining uncertainty

**7.4 Parasite prevalence (18-months follow-up)**


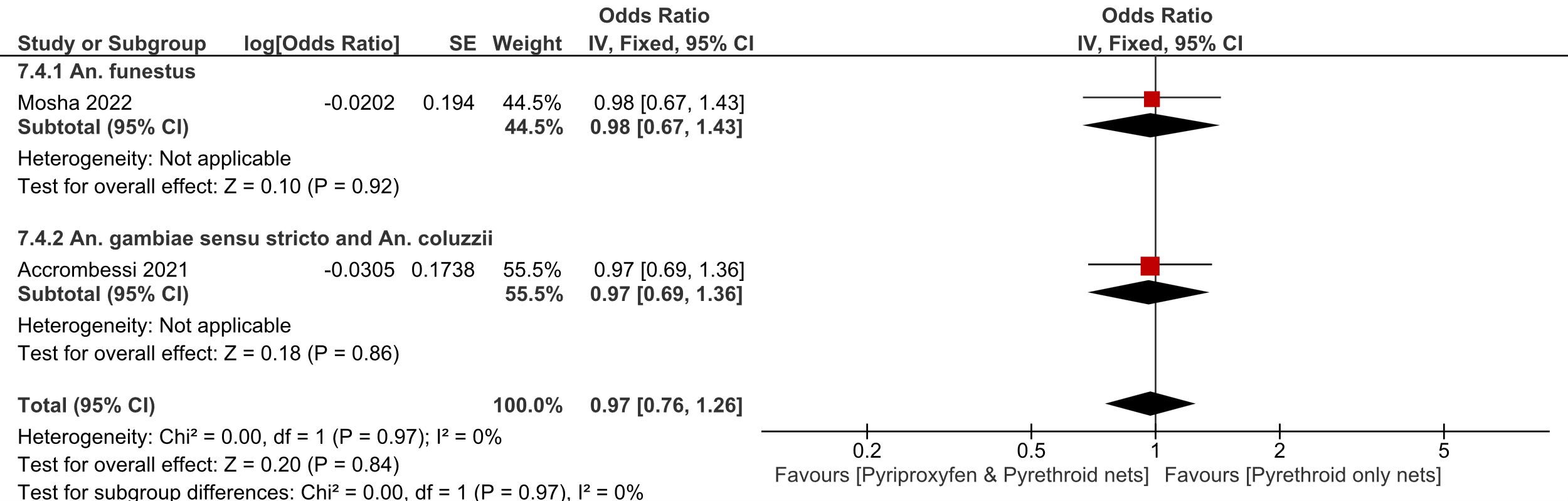


ICEMAN Credibility Assessments

Very Low Credibility. Very likely no effect modification. Use overall effect for each subgroup.

**7.5 Parasite prevalence (furthest possible follow-up)**

**
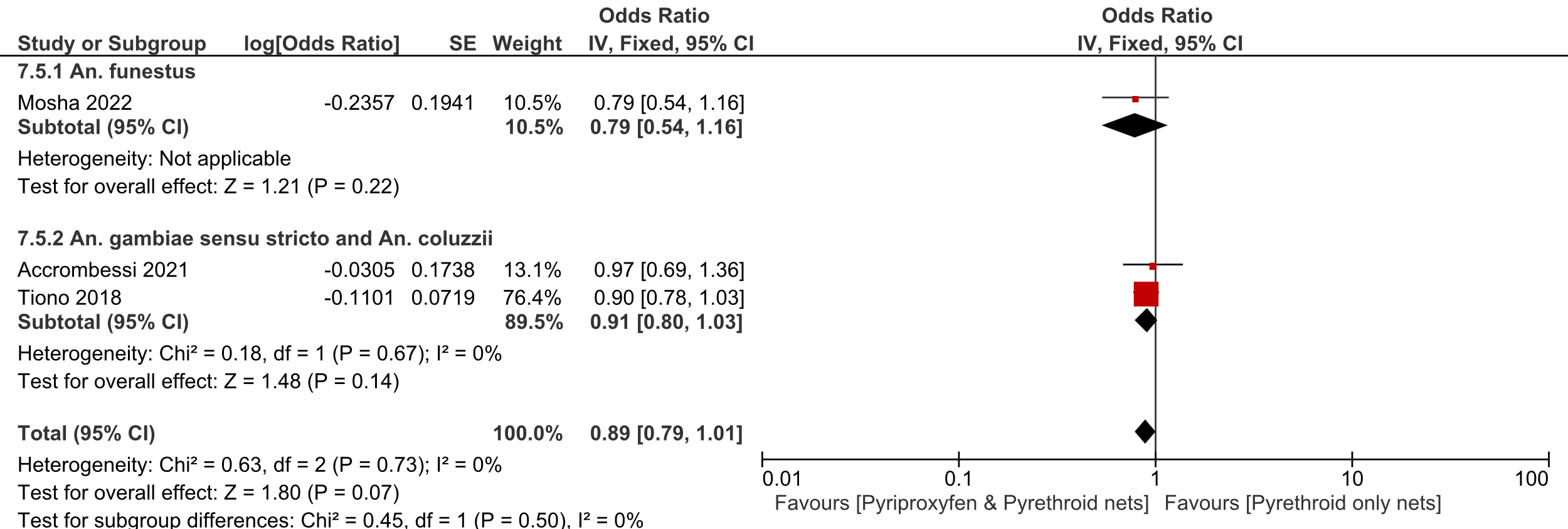
**

ICEMAN Credibility Assessments

Low Credibility. Likely no effect modification. Use overall effect for each subgroup, but note remaining uncertainty

**7.6 Prevalence of anaemia (18-months follow-up)**

**
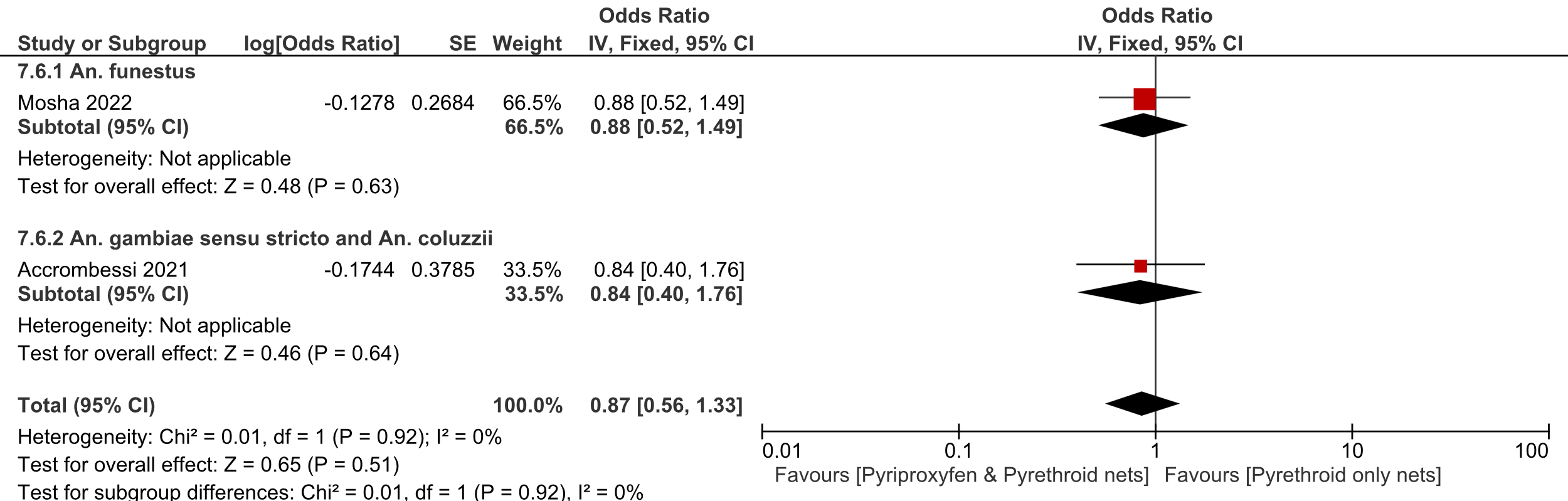
**

ICEMAN Credibility Assessments

Very Low Credibility. Very likely no effect modification. Use overall effect for each subgroup.

**7.7 Prevalence of anaemia (furthest possible follow-up)**

**
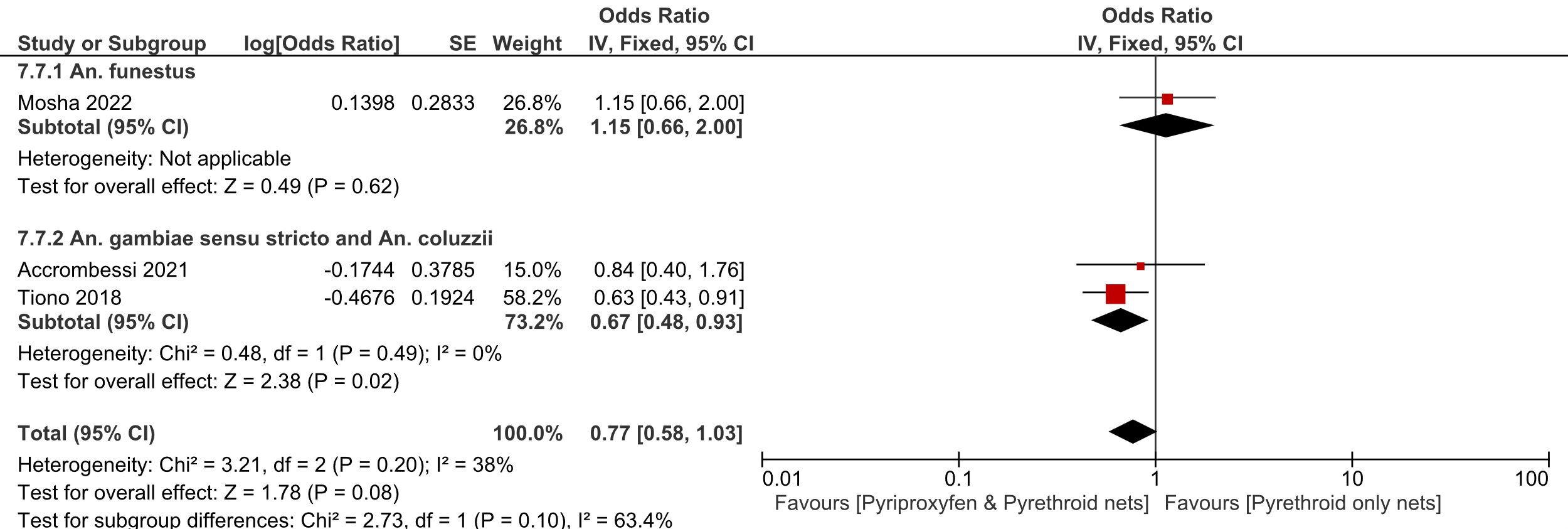
**

ICEMAN Credibility Assessments

Very Low Credibility. Very likely no effect modification. Use overall effect for each subgroup.

**Analysis 8 – Pyriproxyfen-pyrethroid nets versus Pyrethroid-only nets**

**Setting (Rural or Mixed)**

**8.1 Malaria case incidence (overall)**

**
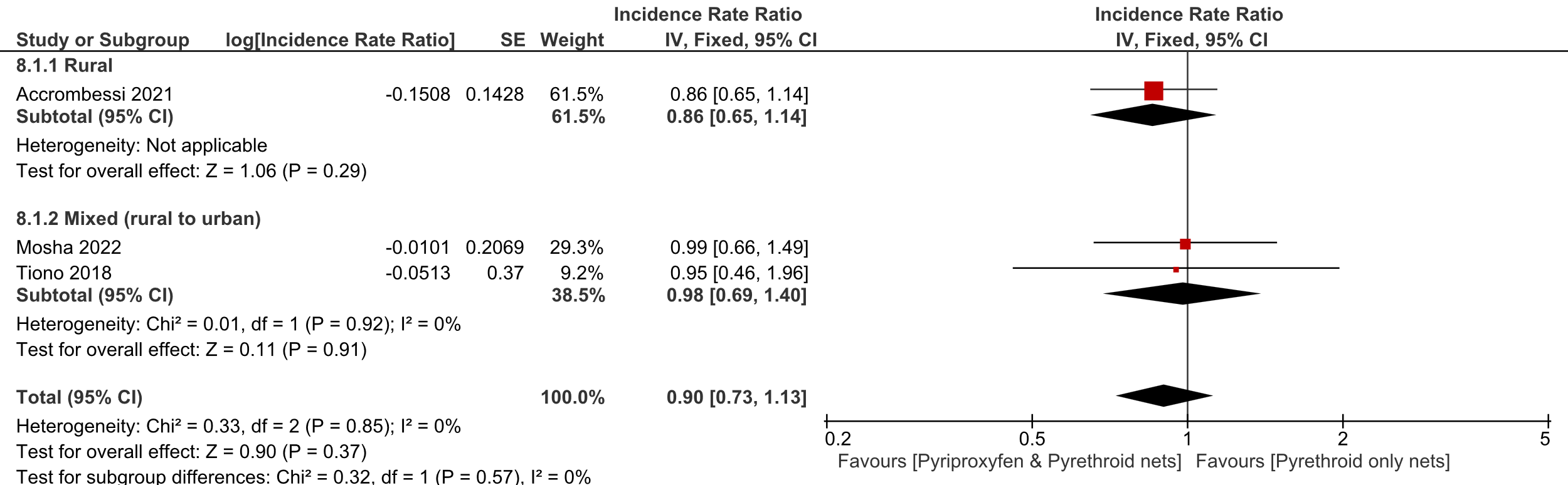
**

ICEMAN Credibility Assessments

Very Low Credibility. Very likely no effect modification. Use overall effect for each subgroup.

**8.2 Malaria case incidence (1-year post)**

**
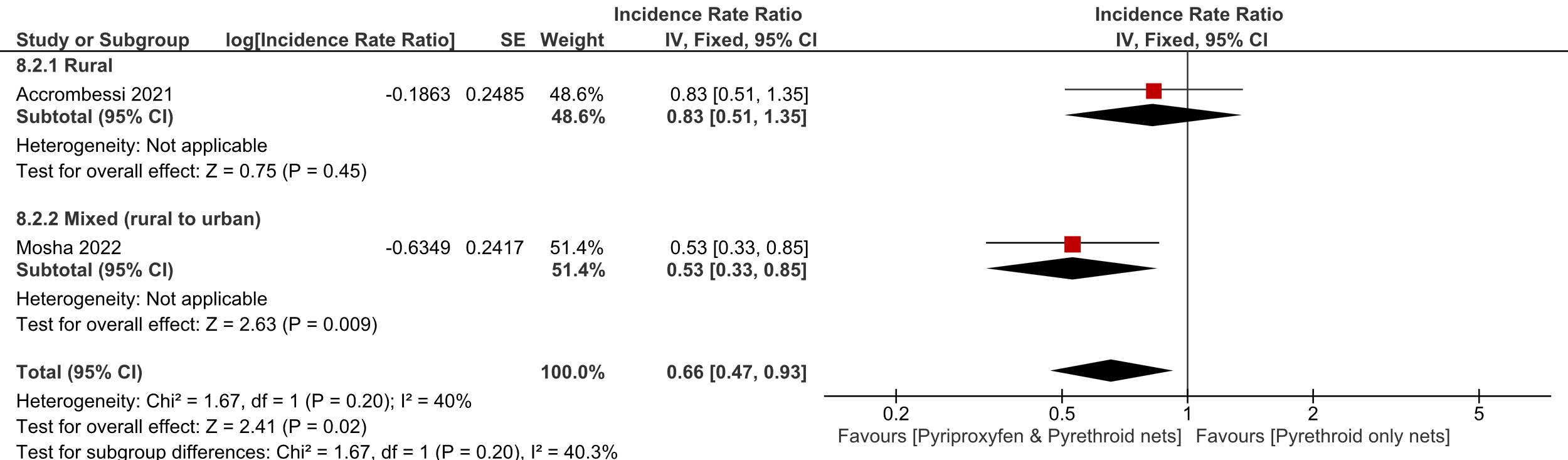
**

ICEMAN Credibility Assessments

Very Low Credibility. Very likely no effect modification. Use overall effect for each subgroup.

**8.3 Malaria case incidence (2-year post)**

**
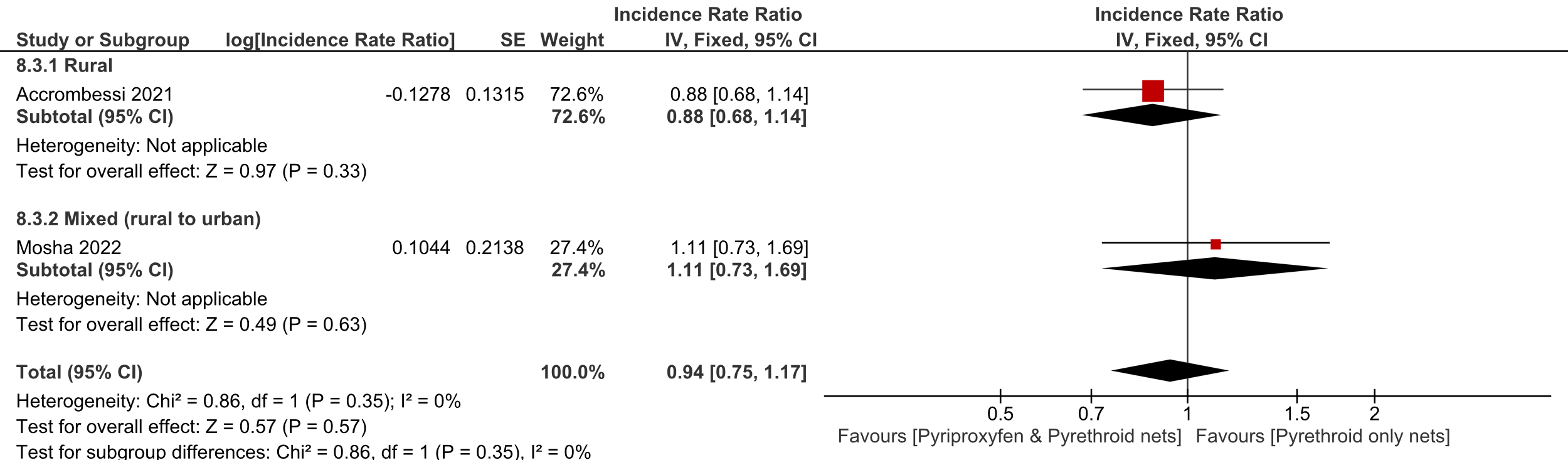
**

ICEMAN Credibility Assessments

Very Low Credibility. Very likely no effect modification. Use overall effect for each subgroup.

**8.4 Parasite prevalence (18-months follow-up)**

**
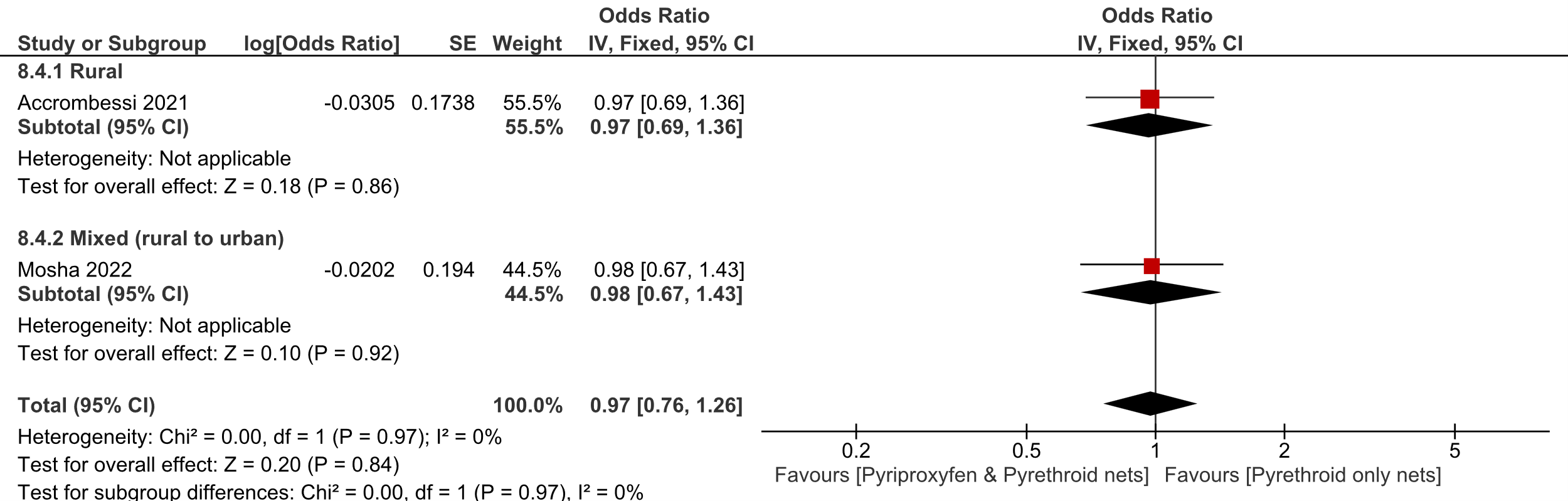
**

ICEMAN Credibility Assessments

Low Credibility. Likely no effect modification. Use overall effect for each subgroup, but note remaining uncertainty

**8.5 Parasite prevalence (furthest possible follow-up)**

**
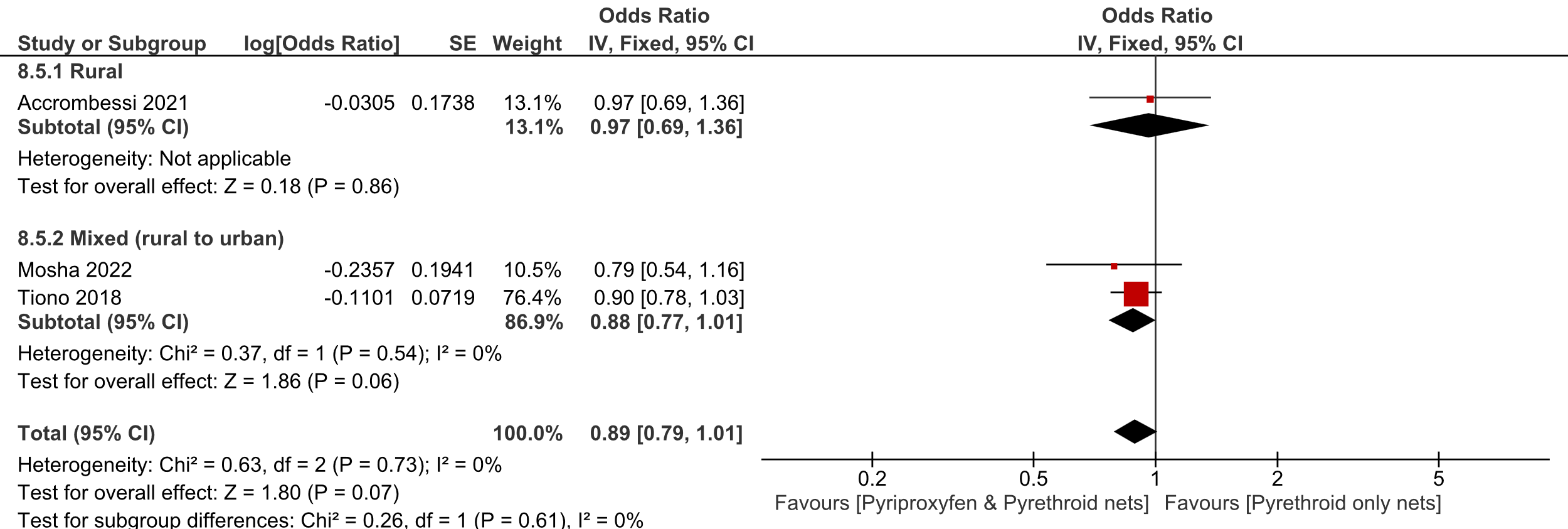
**

ICEMAN Credibility Assessments

Very Low Credibility. Very likely no effect modification. Use overall effect for each subgroup.

**8.6 Prevalence of anaemia (18-months follow-up)**

**
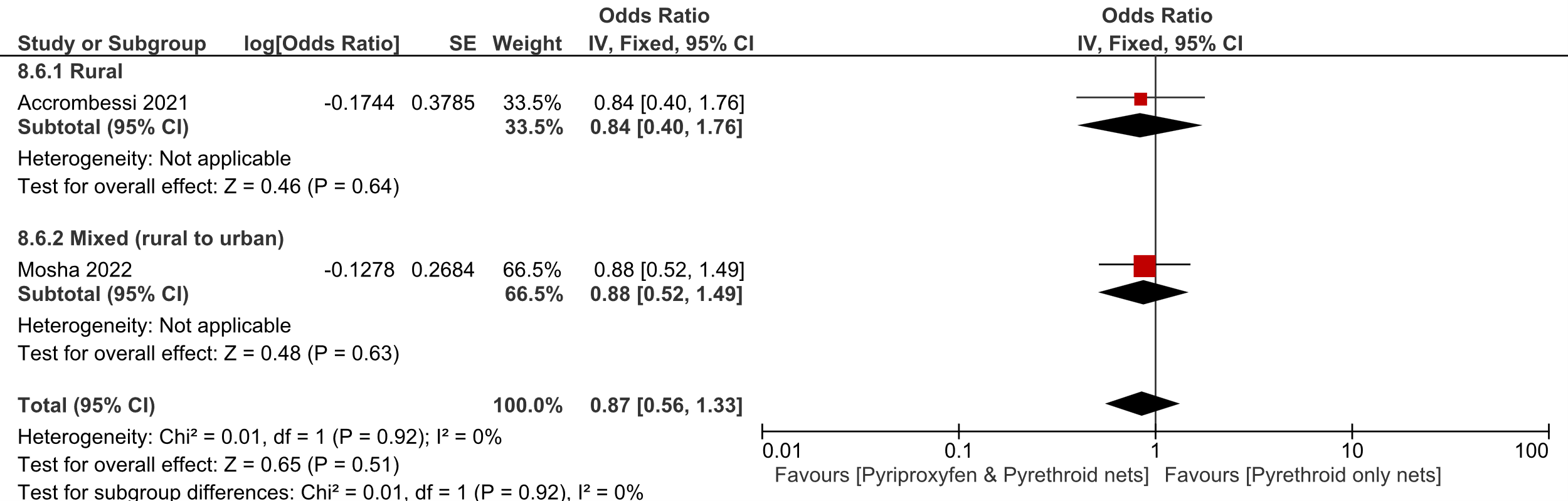
**

ICEMAN Credibility Assessments

Very Low Credibility. Very likely no effect modification. Use overall effect for each subgroup.

**8.7 Prevalence of anaemia (furthest possible follow-up)**

**
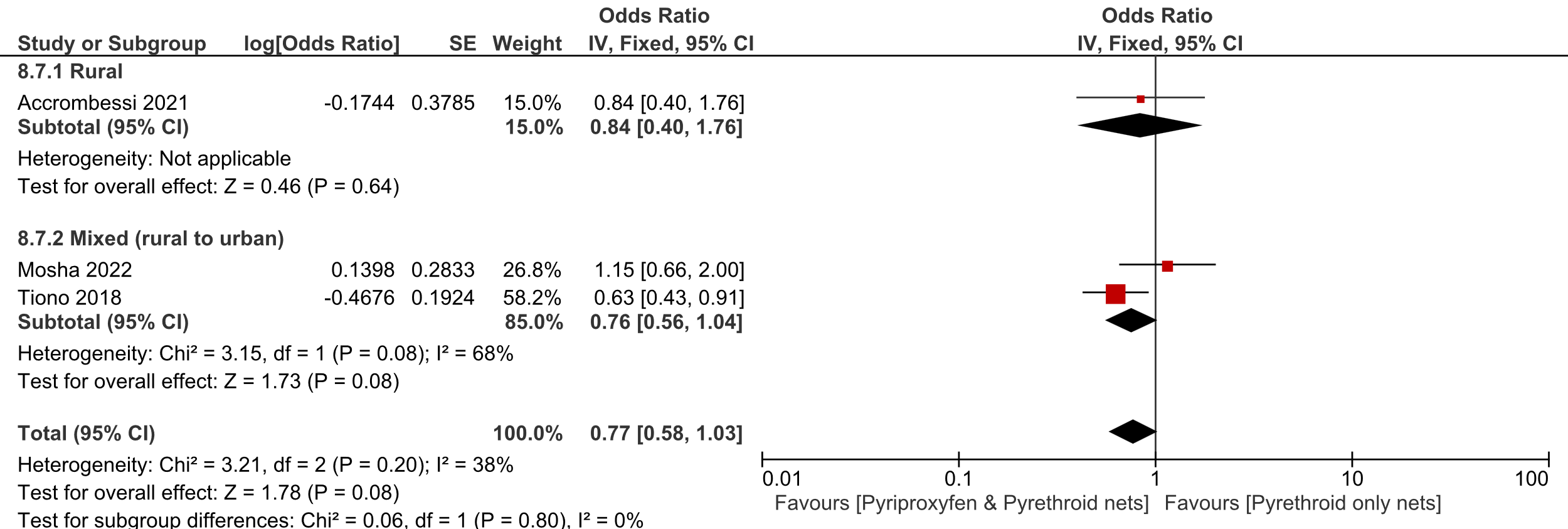
**

ICEMAN Credibility Assessments

Very Low Credibility. Very likely no effect modification. Use overall effect for each subgroup.

# Supporting information 5 – ICEMAN Credibility Assessments

## Active ingredient/manufacturer subgroup

| **Preliminary considerations** |
| --- |
| Study reference(s): |
| If available, protocol reference(s): |
| State a single outcome and, if applicable, time-point of interest (e.g., mortality at 1 year follow-up): |
| State a single effect measure of interest (e.g., relative or absolute risk difference): |
| State a single potential effect modifier of interest (e.g., age or comorbidity): |
| Was the potential effect modifier measured before or at randomization? [ ] yes, continue [ ] no, stop here and refer to manual for further instructions |

| **Credibility assessment** | | | |
| --- | --- | --- | --- |
| **1: Is the analysis of effect modification based on comparison within rather than between trials?** | | | |
| [**x**] Completely between | [ ] Mostly between or unclear | [ ] Mostly within | [ ] Completely within |
| *Subgroup analysis or meta-regression comparing overall effects of each individual trial. This is typical for aggregate data meta-analysis.* | *Subgroup analysis or meta-regression with most information coming from overall effects, but some trials providing within-trial subgroup information* | *Most trials providing within-trial subgroup information; or individual participant data analysis that combines within and between trial information* | *All trials providing within-trial subgroup information or individual participant data; and the analysis separates within from between trial information, e.g., meta-analysis of interactions* |
| Comment: | | | |
| **2: For within-trial comparisons, is the effect modification similar from trial to trial?** [ **x** ] Not applicable: no or one within-RCT comparison | | | |
| [ ] Definitely not similar | [ ] Probably not similar or unclear | [ ] Mostly similar | [ ] Definitely similar |
| *Effect modification reported for two or more trials and clearly different directions* | *Effect modification not reported for individual trials or too imprecise to tell* | *Effect modification reported for two or more trials, mostly similar in direction, but considerable differences in magnitude* | *Effect modification reported for two or more trials, similar in direction, only some differences in magnitude* |
| Comment: | | | |
| **3: For between-trial comparisons, is the number of trials large?** [ ] Not applicable: no between RCT comparison | | | |
| [ **x** ] Very small | [ ] Rather small or unclear | [ ] Rather large | [ ] Large |
| *1 or 2 or in smallest subgroup; 5 or less in continuous meta-regression* | *3-4 in smallest subgroup; 6-10 in continuous meta-regression* | *5-9 in smallest subgroup; 11 to 15 in continuous meta-regression* | *10 or more in smallest subgroup; more than 15 in continuous meta-regression* |
| Comment: | | | |
| **4: Was the direction of effect modification correctly hypothesized a priori?** | | | |
| [ ] Definitely no | [ **x** ] Probably no or unclear | [ ] Probably yes | [ ] Definitely yes |
| *Clearly post-hoc or results inconsistent with hypothesized direction or biologically very implausible* | *Vague hypothesis or hypothesized direction unclear*  *Unclear within studies and not hypothesised for this meta-analysis.* | *No prior protocol available but unequivocal statement of a priori hypothesis with correct direction of effect modification* | *Prior protocol available and includes correct specification of direction of effect modification, e.g., based on a biologic rationale* |
| Comment: | | | |
| **5: Does a test for interaction suggest that chance is an unlikely explanation of the apparent effect modification?** (consider irrespective of number of effect modifiers) | | | |
| [ **x** ] Chance a very likely explanation | [ ] Chance a likely explanation or unclear | [ **x** ] Chance may not explain | [**x**] Chance an unlikely explanation |
| *Interaction or meta-regression p-value >0.05*  ***Royal Guard v Standard***  *Parasite Prevalence*  *Test for differences between subgroups p = 0.57*  ***Interceptor G2 v Standard***  *Malaria Case*  *Test for differences between subgroups p = 0.38* | *Interaction or meta-regression p-value ≤0.05 and >0.01, or no test of interaction reported and not computable* | *Interaction or meta-regression p-value ≤0.01 and >0.005*  ***Royal Guard v Standard***  *Malaria Case*  *Test for differences between subgroups p = 0.02*  *Prevalence of Anaemia*  *Test for differences between subgroups p = 0.08*  ***Interceptor G2 v Standard***  *Prevalence of Anaemia*  *Test for differences between subgroups p = 0.07* | *Interaction or meta-regression p-value ≤0.005*  ***Interceptor G2 v Standard***  *Parasite Prevalence*  *Test for differences between subgroups p = <0.001* |
| Comment: | | | |
| **6: Did the authors test only a small number of effect modifiers or consider the number in their statistical analysis?** | | | |
| [ ] Definitely no | [ ] Probably no or unclear | [ ] Probably yes | [ **x** ] Definitely yes |
| *Explicitly exploratory analysis or large number of effect modifiers tested (e.g., greater than 10) and multiplicity not considered in analysis* | *No mention of number or 4-10 effect modifiers tested and number not considered in analysis* | *No protocol available but unequivocal statement of 3 or fewer effect modifiers tested* | *Protocol available and 3 or fewer effect modifiers tested or number considered in analysis* |
| Comment: | | | |
| **7: Did the authors use a random effects model?** | | | |
| [ **x** ] Definitely no | [ ] Probably no or unclear | [ ] Probably yes | [ ] Definitely yes |
| *Fixed (or common) effect or fixed effects model explicitly stated* | *Probably fixed effect(s) model* | *Probably random (or mixed) effects* | *Random (or mixed) effects explicitly stated* |
| Comment: | | | |
| **8: If the effect modifier is a continuous variable, were arbitrary cut points avoided?** [**x** ] not applicable: not continuous | | | |
| [] Definitely no | [ ] Probably no or unclear | [ ] Probably yes | [ ] Definitely yes |
| *Analysis based on exploratory cut point(s), e.g., picking cut point associated with highest interaction p-value* | *Analysis based on cut point(s) of unclear origin* | *Analysis based on pre-specified cut point(s), e.g., suggested by prior RCT* | *Analysis based on the full continuum, e.g., assuming a linear or logarithmic relationship* |
| Comment: | | | |
| **9 Optional: Are there any additional considerations that may increase or decrease credibility?** (manual section 3.9) [ **x** ] not applicable | | | |
|  | [ ] Yes, probably decrease | [ ] Yes, probably increase | |
| Comment:   \| **10: How would you rate the overall credibility of the proposed effect modification?**  The overall rating should be driven by the items that decrease credibility. The following provides a sensible strategy:   - All responses definitely or probably decrease credibility or unclear 🡪 very low - Two or more responses definitely decrease credibility 🡪 maximum usually low even if all other responses satisfy credibility criteria - One response definitely decreases credibility 🡪 maximum usually moderate even if all other responses satisfy credibility criteria - Two responses probably decrease credibility 🡪 maximum usually moderate even if all other responses satisfy credibility criteria - No response options definitely or probably decrease credibility 🡪 high very likely   Place a mark on the continuous line (or type “x” in editable version) \| \| \| \| \|  \| \| --- \| --- \| --- \| --- \| --- \| --- \| \|  \|  \| \| \| \|  \| \|  \|  \| \| \| \|  \| \|  \|  \| \|  \|  \| \| \| \|  \| \|  \|  \| \| \| \|  \| \|  \| **Very low credibility** \| **Low credibility** \| **Moderate credibility** \| **High credibility** \|  \| \|  \|  \|  \|  \|  \|  \| \|  \| Very likely no effect modification  Use overall effect for each subgroup  ***Royal Guard v Standard***  *Parasite Prevalence*  ***Interceptor G2 v Standard***  *Malaria Case* \| Likely no effect modification  Use overall effect for each subgroup but note remaining uncertainty  ***Royal Guard v Standard***  *Malaria Case*  *Prevalence of Anaemia*  ***Interceptor G2 v Standard***  *Prevalence of Anaemia*  *Parasite Prevalence* \| Likely effect modification  Use separate effects for each subgroup but note remaining uncertainty \| Very likely effect modification  Use separate effects for each subgroup \|  \| \| Comment: \| \| \| \| \| \| | | | |

## Setting subgroup

| **Preliminary considerations** |
| --- |
| Study reference(s): |
| If available, protocol reference(s): |
| State a single outcome and, if applicable, time-point of interest (e.g., mortality at 1 year follow-up): |
| State a single effect measure of interest (e.g., relative or absolute risk difference): |
| State a single potential effect modifier of interest (e.g., age or comorbidity): |
| Was the potential effect modifier measured before or at randomization? [ ] yes, continue [ ] no, stop here and refer to manual for further instructions |

| **Credibility assessment** | | | |
| --- | --- | --- | --- |
| **1: Is the analysis of effect modification based on comparison within rather than between trials?** | | | |
| [ **x** ] Completely between | [ ] Mostly between or unclear | [ ] Mostly within | [ ] Completely within |
| *Subgroup analysis or meta-regression comparing overall effects of each individual trial. This is typical for aggregate data meta-analysis.* | *Subgroup analysis or meta-regression with most information coming from overall effects, but some trials providing within-trial subgroup information* | *Most trials providing within-trial subgroup information; or individual participant data analysis that combines within and between trial information* | *All trials providing within-trial subgroup information or individual participant data; and the analysis separates within from between trial information, e.g., meta-analysis of interactions* |
| Comment: | | | |
| **2: For within-trial comparisons, is the effect modification similar from trial to trial?** [ **x** ] Not applicable: no or one within-RCT comparison | | | |
| [ ] Definitely not similar | [ ] Probably not similar or unclear | [ ] Mostly similar | [ ] Definitely similar |
| *Effect modification reported for two or more trials and clearly different directions* | *Effect modification not reported for individual trials or too imprecise to tell* | *Effect modification reported for two or more trials, mostly similar in direction, but considerable differences in magnitude* | *Effect modification reported for two or more trials, similar in direction, only some differences in magnitude* |
| Comment: | | | |
| **3: For between-trial comparisons, is the number of trials large?** [ ] Not applicable: no between RCT comparison | | | |
| [**x** ] Very small | [ ] Rather small or unclear | [ ] Rather large | [ ] Large |
| *1 or 2 or in smallest subgroup; 5 or less in continuous meta-regression* | *3-4 in smallest subgroup; 6-10 in continuous meta-regression* | *5-9 in smallest subgroup; 11 to 15 in continuous meta-regression* | *10 or more in smallest subgroup; more than 15 in continuous meta-regression* |
| Comment: | | | |
| **4: Was the direction of effect modification correctly hypothesized a priori?** | | | |
| [ ] Definitely no | [ **X** ] Probably no or unclear | [ ] Probably yes | [ ] Definitely yes |
| *Clearly post-hoc or results inconsistent with hypothesized direction or biologically very implausible* | *Vague hypothesis or hypothesized direction unclear* | *No prior protocol available but unequivocal statement of a priori hypothesis with correct direction of effect modification* | *Prior protocol available and includes correct specification of direction of effect modification, e.g., based on a biologic rationale* |
| Comment: | | | |
| **5: Does a test for interaction suggest that chance is an unlikely explanation of the apparent effect modification?** (consider irrespective of number of effect modifiers) | | | |
| [ **x** ] Chance a very likely explanation | [ **x** ] Chance a likely explanation or unclear | [ ] Chance may not explain | [ **x** ] Chance an unlikely explanation |
| *Interaction or meta-regression p-value >0.05*  ***Royal Guard v Standard***  *Malaria Case Incidence (overall)*  *Test for differences between subgroups p = 0.57*  *Malaria Case Incidence (1-year post)*  *Test for differences between subgroups p = 0.20*  *Malaria Case Incidence (2-year post)*  *Test for differences between subgroups p = 0.35*  *Parasite Prevalence (18-months)*  *Test for differences between subgroups p =0.84*  *Parasite Prevalence (furthest possible time point)*  *Test for differences between subgroups p = 0.07*  *Prevalence of anaemia (furthest possible time point)*  *Test for differences between subgroups p = 0.92*  *Prevalence of anaemia (furthest possible time point)*  *Test for differences between subgroups p = 0.80*  ***Interceptor G2 v Standard***  *Malaria Case Incidence (overall)*  *Test for differences between subgroups p = 0.88*  *Malaria Case Incidence (1-year post)*  *Test for differences between subgroups p = 0.94*  *Parasite prevalence (18-months*  *Test for differences between subgroups p = 0.71*  *Parasite Prevalence (furthest possible time point)*  *Test for differences between subgroups p =0.28*  *Prevalence of anaemia (furthest possible time point)*  *Test for differences between subgroups p = 0.39*  *Prevalence of anaemia (furthest possible time point)*  *Test for differences between subgroups p = 0.78* | *Interaction or meta-regression p-value ≤0.05 and >0.01, or no test of interaction reported and not computable* | *Interaction or meta-regression p-value ≤0.01 and >0.005*  ***Interceptor G2 v Standard***  *Malaria Case Incidence (2-year post)*  *Test for differences between subgroups p = 0.05* | *Interaction or meta-regression p-value ≤0.005* |
| Comment: | | | |
| **6: Did the authors test only a small number of effect modifiers or consider the number in their statistical analysis?** | | | |
| [ ] Definitely no | [ ] Probably no or unclear | [ ] Probably yes | [ **x** ] Definitely yes |
| *Explicitly exploratory analysis or large number of effect modifiers tested (e.g., greater than 10) and multiplicity not considered in analysis* | *No mention of number or 4-10 effect modifiers tested and number not considered in analysis* | *No protocol available but unequivocal statement of 3 or fewer effect modifiers tested* | *Protocol available and 3 or fewer effect modifiers tested or number considered in analysis* |
| Comment: | | | |
| **7: Did the authors use a random effects model?** | | | |
| [ **X** ] Definitely no | [ ] Probably no or unclear | [ ] Probably yes | [ ] Definitely yes |
| *Fixed (or common) effect or fixed effects model explicitly stated* | *Probably fixed effect(s) model* | *Probably random (or mixed) effects* | *Random (or mixed) effects explicitly stated* |
| Comment: | | | |
| **8: If the effect modifier is a continuous variable, were arbitrary cut points avoided?** [ **x** ] not applicable: not continuous | | | |
| [ ] Definitely no | [ ] Probably no or unclear | [ ] Probably yes | [ ] Definitely yes |
| *Analysis based on exploratory cut point(s), e.g., picking cut point associated with highest interaction p-value* | *Analysis based on cut point(s) of unclear origin* | *Analysis based on pre-specified cut point(s), e.g., suggested by prior RCT* | *Analysis based on the full continuum, e.g., assuming a linear or logarithmic relationship* |
| Comment: | | | |
| **9 Optional: Are there any additional considerations that may increase or decrease credibility?** (manual section 3.9) [ **x** ] not applicable | | | |
|  | [ ] Yes, probably decrease | [ ] Yes, probably increase | |
| Comment:   \| **10: How would you rate the overall credibility of the proposed effect modification?**  The overall rating should be driven by the items that decrease credibility. The following provides a sensible strategy:   - All responses definitely or probably decrease credibility or unclear 🡪 very low - Two or more responses definitely decrease credibility 🡪 maximum usually low even if all other responses satisfy credibility criteria - One response definitely decreases credibility 🡪 maximum usually moderate even if all other responses satisfy credibility criteria - Two responses probably decrease credibility 🡪 maximum usually moderate even if all other responses satisfy credibility criteria - No response options definitely or probably decrease credibility 🡪 high very likely   Place a mark on the continuous line (or type “x” in editable version) \| \| \| \| \|  \| \| --- \| --- \| --- \| --- \| --- \| --- \| \|  \|  \| \| \| \|  \| \|  \|  \| \| \| \|  \| \|  \|  \| \|  \|  \| \| \| \|  \| \|  \|  \| \| \| \|  \| \|  \| **Very low credibility** \| **Low credibility** \| **Moderate credibility** \| **High credibility** \|  \| \|  \|  \|  \|  \|  \|  \| \|  \| Very likely no effect modification  Use overall effect for each subgroup  ***Royal Guard v Standard***  *Malaria Case Incidence (overall)*  *Malaria Case Incidence (1-year post)*  *Malaria Case Incidence (2-year post)*  *Parasite Prevalence (furthest possible follow-up)*  *Prevalence of anaemia (18-months follow-up)*  *Prevalence of anaemia (furthest possible follow-up)*  ***Interceptor G2 v Standard***  *Malaria Case Incidence (1-year post)*  *Malaria case Incidence (2-years post)*  *Parasite Prevalence (18-months follow-up)*  *Prevalence of anaemia (18-months follow-up)*  *Prevalence of anaemia (furthest possible follow-up)* \| Likely no effect modification  Use overall effect for each subgroup but note remaining uncertainty \| Likely effect modification  Use separate effects for each subgroup but note remaining uncertainty \| Very likely effect modification  Use separate effects for each subgroup \|  \| \| Comment: \| \| \| \| \| \| | | | |

## Vector subgroup

| **Preliminary considerations** |
| --- |
| Study reference(s): |
| If available, protocol reference(s): |
| State a single outcome and, if applicable, time-point of interest (e.g., mortality at 1 year follow-up): |
| State a single effect measure of interest (e.g., relative or absolute risk difference): |
| State a single potential effect modifier of interest (e.g., age or comorbidity): |
| Was the potential effect modifier measured before or at randomization? [ ] yes, continue [ ] no, stop here and refer to manual for further instructions |

| **Credibility assessment** | | | |
| --- | --- | --- | --- |
| **1: Is the analysis of effect modification based on comparison within rather than between trials?** | | | |
| [ **x** ] Completely between | [ ] Mostly between or unclear | [ ] Mostly within | [ ] Completely within |
| *Subgroup analysis or meta-regression comparing overall effects of each individual trial. This is typical for aggregate data meta-analysis.* | *Subgroup analysis or meta-regression with most information coming from overall effects, but some trials providing within-trial subgroup information* | *Most trials providing within-trial subgroup information; or individual participant data analysis that combines within and between trial information* | *All trials providing within-trial subgroup information or individual participant data; and the analysis separates within from between trial information, e.g., meta-analysis of interactions* |
| Comment: | | | |
| **2: For within-trial comparisons, is the effect modification similar from trial to trial?** [ **x** ] Not applicable: no or one within-RCT comparison | | | |
| [ ] Definitely not similar | [ ] Probably not similar or unclear | [ ] Mostly similar | [ ] Definitely similar |
| *Effect modification reported for two or more trials and clearly different directions* | *Effect modification not reported for individual trials or too imprecise to tell* | *Effect modification reported for two or more trials, mostly similar in direction, but considerable differences in magnitude* | *Effect modification reported for two or more trials, similar in direction, only some differences in magnitude* |
| Comment: | | | |
| **3: For between-trial comparisons, is the number of trials large?** [ ] Not applicable: no between RCT comparison | | | |
| [ **x** ] Very small | [ ] Rather small or unclear | [ ] Rather large | [ ] Large |
| *1 or 2 or in smallest subgroup; 5 or less in continuous meta-regression* | *3-4 in smallest subgroup; 6-10 in continuous meta-regression* | *5-9 in smallest subgroup; 11 to 15 in continuous meta-regression* | *10 or more in smallest subgroup; more than 15 in continuous meta-regression* |
| Comment: | | | |
| **4: Was the direction of effect modification correctly hypothesized a priori?** | | | |
| [ ] Definitely no | [ **x** ] Probably no or unclear | [ ] Probably yes | [ ] Definitely yes |
| *Clearly post-hoc or results inconsistent with hypothesized direction or biologically very implausible* | *Vague hypothesis or hypothesized direction unclear* | *No prior protocol available but unequivocal statement of a priori hypothesis with correct direction of effect modification* | *Prior protocol available and includes correct specification of direction of effect modification, e.g., based on a biologic rationale* |
| Comment: | | | |
| **5: Does a test for interaction suggest that chance is an unlikely explanation of the apparent effect modification?** (consider irrespective of number of effect modifiers) | | | |
| [ **x** ] Chance a very likely explanation | [ ] Chance a likely explanation or unclear | [ **x** ] Chance may not explain | [ ] Chance an unlikely explanation |
| *Interaction or meta-regression p-value >0.05*  ***Royal Guard v Standard***  *Malaria Case Incidence (overall)*  *Test for differences between subgroups p = 0.60*  *Malaria Case Incidence (1-year post)*  *Test for differences between subgroups p = 0.20*  *Malaria Case Incidence (2-year post)*  *Test for differences between subgroups p = 0.35*  *Parasite Prevalence (18-months follow-up*  *Test for differences between subgroups p = 0.97*  *Parasite Prevalence (furthest possible follow-up)*  *Test for differences between subgroups p = 0.50*  *Prevalence of anaemia (18-months follow-up)*  *Test for differences between subgroups p = 0.92*  *Prevalence of anaemia (furthest possible follow-up)*  *Test for differences between subgroups p = 0.10*  ***Interceptor G2 v Standard***  *Malaria Case Incidence (overall)*  *Test for differences between subgroups p = 0.88*  *Malaria Case Incidence (1- year post)*  *Test for differences between subgroups p = 0.94*  *Parasite Prevalence (18-months follow-up*  *Test for differences between subgroups p = 0.71*  *Parasite Prevalence (furthest possible follow-up)*  *Test for differences between subgroups p = 0.28*  *Prevalence of anaemia (18-months follow-up)*  *Test for differences between subgroups p = 0.39*  *Prevalence of anaemia (furthest possible follow-up)*  *Test for differences between subgroups p = 0.78* | *Interaction or meta-regression p-value ≤0.05 and >0.01, or no test of interaction reported and not computable* | *Interaction or meta-regression p-value ≤0.01 and >0.005*  ***Interceptor G2 v Standard***  *Malaria Case Incidence (2- year post)*  *Test for differences between subgroups p = 0.05* | *Interaction or meta-regression p-value ≤0.005* |
| Comment: | | | |
| **6: Did the authors test only a small number of effect modifiers or consider the number in their statistical analysis?** | | | |
| [ ] Definitely no | [ ] Probably no or unclear | [ ] Probably yes | [ **x** ] Definitely yes |
| *Explicitly exploratory analysis or large number of effect modifiers tested (e.g., greater than 10) and multiplicity not considered in analysis* | *No mention of number or 4-10 effect modifiers tested and number not considered in analysis* | *No protocol available but unequivocal statement of 3 or fewer effect modifiers tested* | *Protocol available and 3 or fewer effect modifiers tested or number considered in analysis* |
| Comment: | | | |
| **7: Did the authors use a random effects model?** | | | |
| [ **x** ] Definitely no | [ ] Probably no or unclear | [ ] Probably yes | [ ] Definitely yes |
| *Fixed (or common) effect or fixed effects model explicitly stated* | *Probably fixed effect(s) model* | *Probably random (or mixed) effects* | *Random (or mixed) effects explicitly stated* |
| Comment: | | | |
| **8: If the effect modifier is a continuous variable, were arbitrary cut points avoided?** [ **x** ] not applicable: not continuous | | | |
| [ ] Definitely no | [ ] Probably no or unclear | [ ] Probably yes | [ ] Definitely yes |
| *Analysis based on exploratory cut point(s), e.g., picking cut point associated with highest interaction p-value* | *Analysis based on cut point(s) of unclear origin* | *Analysis based on pre-specified cut point(s), e.g., suggested by prior RCT* | *Analysis based on the full continuum, e.g., assuming a linear or logarithmic relationship* |
| Comment: | | | |
| **9 Optional: Are there any additional considerations that may increase or decrease credibility?** (manual section 3.9) [ ] not applicable | | | |
|  | [ ] Yes, probably decrease | [ ] Yes, probably increase | |
| Comment:   \| **10: How would you rate the overall credibility of the proposed effect modification?**  The overall rating should be driven by the items that decrease credibility. The following provides a sensible strategy:   - All responses definitely or probably decrease credibility or unclear 🡪 very low - Two or more responses definitely decrease credibility 🡪 maximum usually low even if all other responses satisfy credibility criteria - One response definitely decreases credibility 🡪 maximum usually moderate even if all other responses satisfy credibility criteria - Two responses probably decrease credibility 🡪 maximum usually moderate even if all other responses satisfy credibility criteria - No response options definitely or probably decrease credibility 🡪 high very likely   Place a mark on the continuous line (or type “x” in editable version) \| \| \| \| \|  \| \| --- \| --- \| --- \| --- \| --- \| --- \| \|  \|  \| \| \| \|  \| \|  \|  \| \| \| \|  \| \|  \|  \| \|  \|  \| \| \| \|  \| \|  \|  \| \| \| \|  \| \|  \| **Very low credibility** \| **Low credibility** \| **Moderate credibility** \| **High credibility** \|  \| \|  \|  \|  \|  \|  \|  \| \|  \| Very likely no effect modification  Use overall effect for each subgroup  ***Royal Guard***  *Malaria Case Incidence (overall)*  *Malaria Case Incidence (1-year post)*  *Malaria Case Incidence (2-year post)*  *Parasite Prevalence (18-month follow-up)*  *Parasite Prevalence (furthest possible follow-up)*  *Prevalence of anaemia (furthest possible follow-up)*  ***Interceptor G2***  *Malaria Case Incidence (overall)*  *Parasite Prevalence (furthest possible follow-up)*  *Malaria Case Incidence (1-year)*  *Malaria Case Incidence (2-year)*  *Parasite Prevalence (18-month follow-up)*  *Prevalence of anaemia (18-months follow-up*  *Prevalence of anaemia (furthest possible follow-up)* \| Likely no effect modification  Use overall effect for each subgroup but note remaining uncertainty \| Likely effect modification  Use separate effects for each subgroup but note remaining uncertainty \| Very likely effect modification  Use separate effects for each subgroup \|  \| \| Comment: \| \| \| \| \| \| | | | |
