# Supplementary material for: Effectiveness of dual active ingredient insecticide-treated nets in preventing malaria: A systematic review and meta-analysis
Source: PLoS One. 2023 Aug 16;18(8):e0289469. doi: 10.1371/journal.pone.0289469 (PMC10431665; doi:10.1371/journal.pone.0289469)
Supplement: Attachment — Submitted filename: Response to Reviewers.docx [file pone.0289469.s003.docx]

**Response to Reviewers – Accompanying Manuscript PONE-D-23-09253**

**Effectiveness of dual active ingredient insecticide-treated nets in preventing malaria: a systematic review and meta-analysis**

**Editor’s comments**

*1. Please ensure that your manuscript meets PLOS ONE's style requirements, including those for file naming. The PLOS ONE style templates can be found at* 
[*https://journals.plos.org/plosone/s/file?id=wjVg/PLOSOne_formatting_sample_main_body.pdf*](https://protect-au.mimecast.com/s/ar9nCGv0q2FjPR3YIQJ__h?domain=journals.plos.org) *and* 
[*https://journals.plos.org/plosone/s/file?id=ba62/PLOSOne_formatting_sample_title_authors_affiliations.pdf*](https://protect-au.mimecast.com/s/BwwbCJyBw2h02v9rfvSZvI?domain=journals.plos.org)

We believe that the revised manuscript now meets the PLOS ONE style requirements.

*2. We note that you have stated that you will provide repository information for your data at acceptance. Should your manuscript be accepted for publication, we will hold it until you provide the relevant accession numbers or DOIs necessary to access your data. If you wish to make changes to your Data Availability statement, please describe these changes in your cover letter and we will update your Data Availability statement to reflect the information you provide.*

We wish to make no changes to this information as presented in the revised manuscript

*3. Please note that funding information should not appear in any section or other areas of your manuscript. We will only publish funding information present in the Funding Statement section of the online submission form. Please remove any funding-related text from the manuscript.*

We have removed all detail regarding funding information from the manuscript.

*4. Please include a separate caption for each figure in your manuscript.*

The revised version of the manuscript has included captions as per the instructions for authors.

*5. Please include captions for your Supporting Information files at the end of your manuscript, and update any in-text citations to match accordingly. Please see our Supporting Information guidelines for more information:* [*http://journals.plos.org/plosone/s/supporting-information*](https://protect-au.mimecast.com/s/amrhCK1Dx2i3YN7EuGujam?domain=journals.plos.org)*.*

We have now included captions for the supporting information files at the end of the manuscript and have updated all the in-text citations accordingly.

**Reviewer #1**

**Major comments**

*1. I regret to say that the analysis underpinning this review contains a major flaw that is likely to invalidate at least some of the conclusions they draw. All three trials that contributed to this review were cluster randomised, and the authors correctly state that analysis of such data needs to take account of their clustered nature in calculating standard errors (SEs) and hence 95% Confidence Intervals. The authors note that there was “No need to adjust standard errors for failing to account for clustering as all studies had done so appropriately”. Unfortunately they then do not use the appropriate 95%CI that were reported in the studies and which allow for appropriate inflation of SEs, but calculate their own, so-called ‘exact 95% CI’ from the crude numbers given in the tables of the papers without taking account of the clustered nature from which these numbers are aggregated. I checked a number of 95% CIs that are cited in the review and all were identical to what one would get from assuming a simple random sample of independent observations, rather than a set of observations that are correlated within clusters. It is unclear why the 95% CIs that are cited in the papers, and which the authors confirm as taking account of clustering, were replaced by the narrower CIs that ignore clustering. Where data are pooled across studies, it appears that 95% CI were computed equivalent to adding the corresponding cases and denominators from the constituent studies, and calculating SEs assuming simple binomial or Poisson distributions (depending on outcome type), with no between cluster variance component. As a consequence, all 95% CIs are too narrow and need to be recalculated.
In some cases the corrected results are likely not to lead to a different interpretation regarding the superiority of the new nets, in others it is highly likely that a different conclusion will need to be drawn. For example, for the Royal Guard pyriproxyfen net the two papers that contribute outcomes for malaria incidence (Accrombessi et al and Mosha et al) for the comparison with the standard pyrethroid net, the overall results cited in each of the two papers, are given as incidence rate ratios [95% Cis] of 0·86 [ 0·65–1·14]. p= 0·28 (Accrombessi) and 0·99 (0·66–1·50), p=0·9801 (Mosha), i.e. no evidence against the null hypothesis of no effect in each trial alone. This changes dramatically if one calculates exact 95% CIs that ignore the lack of independence in the data (Fig 25 of the manuscript), and the authors come up with a highly significant superior protection afforded by this net, with an IRR of 0.86 [0.78- 0.94]. It is likely that the corrected result would show little or no evidence of superiority of Royal Guard over the standard pyrethroid net once the analysis takes account of clustering. Therefore, erroneously this net has been recommended as providing better protection based on ‘strong evidence’.

The conclusion “the evidence also suggests that clusters that receive a pyriproxyfen-pyrethroid ITN (figure 25) will result in a reduction of malaria case incidence compared to clusters that receive a pyrethroid-only ITN.... This finding was associated with high certainty of the evidence” (page 43) therefore seems highly unlikely. This error can have important practical consequences, since international donors purchase nets based on WHO guidance. Therefore, in addition to correcting the meta analysis for this paper, it is incumbent on the authors to contact WHO to inform them that their recently promulgated recommendation has been based on a flawed analysis and may need to be withdrawn.

The errors are simple to remedy and I am sure the authors need no advice on this: if the correct 95% CIs cited in the published papers together with the raw numbers of cases and denominators were entered into the meta-analysis software, it will calculate the appropriate inflation that is applied to the standard errors for each study and hence apply this to the pooled analysis – I am certain the authors will know exactly what needs to be done (and probably wonder how this slip-up occurred). However, in its current form the paper cannot be published, and the discussion and conclusion will need to reflect a revised set of results, possibly contradicting some of the current recommendations.
As the authors are highly experienced in this type of work, this is probably just a glitch, but one with significant consequences for the findings.*

We sincerely thank the reviewer for the care they have taken to review these meta-analyses. There were some substantial corrections required in the presentation of the data in this revised manuscript. To explain our process, this was a review commissioned by the WHO and in doing this review, we utilised pre-published, raw data where applicable to facilitate the analyses. These initial analyses were conducted to inform the WHO decision making as the reviewer has identified, with the WHO understanding that it was preliminary data. However, we had overlooked updating this data to account for the adjusted estimates following availability of the published data (late-January 2023).

We acknowledge that we should have identified this prior to submission and appreciate the reviewer for identifying the error. We have completely re-conducted all the analyses (where appropriate) to account for the appropriate adjusted estimates and can provide the .rm5 file with the supplementary material for transparency and further review if required.

For the comparisons of Chlorfenapyr & Pyrethroid nets versus Pyrethroid & PBO nets, and Pyriproxyfen & Pyrethroid nets versus Pyrethroid & PBO nets, the single included study that provided data regarding this comparison did not actually provide adjusted estimates and the raw data provided by these studies is still used in the analysis presented. However, we have now changed the measure of effect presented to an odds ratio (where applicable) for consistency with the other changed outcomes and have rated the certainty of the evidence for each outcome under these comparisons down by one additional level for the domain of risk of bias, as adjusted estimates were not used.

Four outcomes have changed that warrant important consideration as the conclusions drawn from the unadjusted data do change when considering the appropriate adjusted estimates, all four of these outcomes belong under the comparison of Pyriproxyfen & Pyrethroid nets versus Pyrethroid only nets. The outcomes that have changed include:

1. Malaria Case Incidence (Overall) – Finding is no longer significant, have downgraded two levels for imprecision
2. Malaria Case Incidence (2-years) – Finding remains insignificant, but have downgraded two levels for imprecision, as opposed to one.
3. Parasite Prevalence (12-months) – Finding is no longer significant, have downgraded one level for imprecision.
4. Parasite Prevalence (24-months) - Finding is no longer significant, have downgraded one level for imprecision

Overall, there have been substantial changes made throughout the manuscript to reflect the new analyses, however we believe that the conclusions drawn from these new analyses will only change for the outcomes above. The final pooled estimates of each analysis have been provided below, detailing how they have changed from the previously submitted version. Similarly, we have also included the certainty of evidence for each outcome, and detailed how it has changed.

Chlorfenapyr & Pyrethroid nets vs Pyrethroid only nets

*Malaria Case Incidence (Overall):* IRR = 0.55, 95%CI: 0.44-0.68 [Was – IRR = 0.52, 95%CI: 0.48-0.57]

*Certainty of Evidence:* Changed [High] – Increased certainty as no longer heterogenous.

*Malaria Case Incidence (1-year post):* IRR = 0.47, 95%CI: 0.35-0.63 [Was – IRR = 0.44, 95%CI: 0.37-0.52]

*Certainty of Evidence:* No change [High]

*Malaria Case Incidence (2-year post):* IRR = 0.67, 95%CI: 0.61-0.74 [Was – IRR = 0.57, 95%CI: 0.51-0.63]

*Certainty of Evidence:* No change [High]

*Parasite Prevalence (6-month):* OR = 0.47, 95%CI: 0.32-0.69 [Was – RR = 0.56, 95%CI: 0.48-0.65]

*Certainty of Evidence:* No change [High]

*Parasite Prevalence (12-month):* OR = 0.47, 95%CI: 0.31-0.71 [Was – RR = 0.78, 95%CI: 0.72-0.85]

*Certainty of Evidence:* No change [High]

*Parasite Prevalence (18-month):* OR = 0.63, 95%CI: 0.49-0.80 [Was – RR = 0.75, 95%CI: 0.70-0.80]

*Certainty of Evidence:* No change [High]

*Parasite Prevalence (24-month):* OR = 0.45, 95%CI: 0.30-0.68 [Was – RR = 0.56, 95%CI: 0.50-0.63]

*Certainty of Evidence:* No change [High]

*Parasite Prevalence (furthest):* OR = 0.53, 95%CI: 0.41-0.69 [Was – RR = 0.64, 95%CI: 0.59-0.69]

*Prevalence of anaemia (6-month):* OR = 0.71, 95%CI: 0.40-1.26 [Was – RR = 0.83, 95%CI: 0.66-1.04]

*Prevalence of anaemia (12-month):* OR = 1.55, 95%CI: 0.58-4.14 [Was – RR = 1.48, 95%CI: 0.58-3.79]

*Prevalence of anaemia (18-month):* OR = 0.83, 95%CI: 0.53-1.28 [Was – RR = 0.95, 95%CI: 0.80-1.13]

*Prevalence of anaemia (24-month):* OR = 0.94, 95%CI: 0.52-1.70 [Was – RR = 0.79, 95%CI: 0.48-1.3]

*Prevalence of anaemia (furthest):* OR = 0.99, 95%CI: 0.62-1.58 [Was – RR = 0.97, 95%CI: 0.81-1.16]

Chlorfenapyr & Pyrethroid nets vs Pyrethroid & PBO nets (NO CHANGE)

*Malaria Case Incidence (Overall):* No change in effect estimate

*Certainty of Evidence:* Changed [Moderate] from [High]

*Malaria Case Incidence (1-year post):* No change in effect estimate

*Certainty of Evidence:* Changed [Very Low] from [Low]

*Malaria Case Incidence (2-year post):* No change in effect estimate

*Certainty of Evidence:* Changed [Moderate] from [High]

*Parasite Prevalence (12-month):* OR = 0.78, 95%CI: 0.62-0.97 [Was – RR = 0.81, 95%CI: 0.68-0.98]

*Certainty of Evidence:* Changed [Low] from [Moderate]

*Parasite Prevalence (18-month):* OR = 0.91, 95%CI: 0.77-1.04 [Was – RR = 0.94, 95%CI: 0.86-1.04]

*Certainty of Evidence:* Changed [Low] from [Moderate]

*Parasite Prevalence (24-month):* OR = 0.50, 95%CI: 0.42-0.60 [Was – RR = 0.63, 95%CI: 0.56-0.71]

*Certainty of Evidence:* Changed [Moderate] from [Low]

Pyriproxyfen & Pyrethroid nets vs Pyrethroid only nets

*Malaria Case Incidence (Overall):* IRR = 0.90, 95%CI: 0.73-1.13 [Was – IRR = 0.86, 95%CI: 0.81-0.92]

*Certainty of Evidence:* Changed [Low] from [High]

*Malaria Case Incidence (1-year post):* IRR: 0.66, 95%CI: 0.47-0.85 [Was – IRR = 0.81, 95%CI: 0.70-0.93]

*Certainty of Evidence:* No change [High]

*Malaria Case Incidence (2-year post):* IRR: 0.94, 95%CI: 0.75-1.17 [Was – IRR = 0.91, 95%CI: 0.81-1.02]

*Certainty of Evidence:* Changed [Low] from [Moderate]

*Parasite Prevalence (6-month):* OR: 0.92, 95%CI: 0.63-1.34 [Was – RR = 0.96, 95%CI: 0.85-1.08]

*Certainty of Evidence:* No change [Moderate]

*Parasite Prevalence (12-month):* OR: 0.69, 95%CI: 0.46-1.04 [Was – RR = 0.70, 95%CI: 0.60-0.80]

*Certainty of Evidence:* Changed [Moderate] from [High]

*Parasite Prevalence (18-month):* OR: 0.97, 95%CI: 0.76-1.26 [Was – RR = 0.98, 95%CI: 0.92-1.04]

*Certainty of Evidence:* No change [Low]

*Parasite Prevalence (24-month):* OR: 0.79, 95%CI: 0.54-1.16 [Was – RR = 0.82, 95%CI: 0.75-0.90]

*Certainty of Evidence:* Changed [Moderate] from [High]

*Parasite Prevalence (furthest possible):* OR: 0.89, 95%CI: 0.79-1.01 [Was – RR = 0.91, 95%CI: 0.82-1.01]

*Prevalence of anaemia (6-month):* OR: 1.24, 95%CI: 0.71-2.17 [Was – RR = 1.14, 95%CI: 0.93-1.39]

*Prevalence of anaemia (12-month):* OR: 1.15, 95%CI: 0.40-3.31 [Was – RR = 1.22, 95%CI: 0.45-3.33]

*Prevalence of anaemia (18-month):* OR: 0.87, 95%CI: 0.56-1.33 [Was – RR = 0.94, 95%CI: 0.79-1.13]

*Prevalence of anaemia (24-month):* OR: 1.15, 95%CI: 0.66-2.00 [Was – RR = 1.02, 95%CI: 0.64-1.62]

*Prevalence of anaemia (furthest possible):* OR: 0.77, 95%CI: 0.58-1.03 [Was – RR = 0.85, 95%CI: 0.72-1.00]

Pyriproxyfen & Pyrethroid nets vs Pyrethroid & PBO nets

*Malaria Case Incidence (Overall):* [Was – IRR = 1.25, 95%CI: 1.10-1.41]

*Certainty of Evidence:* Changed [Moderate] from [High]

*Malaria Case Incidence (1-year post):* [Was – IRR = 2.04, 95%CI: 1.55-2.68]

*Certainty of Evidence:* Changed [Moderate] from [High]

*Malaria Case Incidence (2-year post):* [Was – IRR = 1.10, 95%CI: 0.95-1.27]

*Certainty of Evidence:* Changed [Low] from [Moderate]

*Parasite Prevalence (12-month):* OR = 1.16, 95%CI:0.94-1.44 [Was – RR = 1.13, 95%CI: 0.95-1.33]

*Certainty of Evidence:* Changed [Low] from [Moderate]

*Parasite Prevalence (18-month):* OR = 1.34, 95%CI: 1.14-1.58 [Was – RR = 1.17, 95%CI: 1.07-1.27]

*Certainty of Evidence:* Changed [Moderate] from [High]

*Parasite Prevalence (24-month):* OR = 0.88, 95%CI 0.75-1.03 [Was – RR = 0.92, 95%CI: 0.84-1.02]

*Certainty of Evidence:* Changed [Low] from [Moderate]

*Prevalence of anaemia (12-month):* OR = 0.80, 95%CI: 0.31-2.04 [Was – RR = 0.80, 95%CI: 0.32-2.00]

*Prevalence of anaemia (18-month):* OR = 1.67, 95%CI: 0.97-2.87 [Was – RR = 1.60, 95%CI: 0.97-2.65]

*Prevalence of anaemia (24-month):* OR = 1.38, 95%CI: 0.82-2.32 [Was – RR = 1.35, 95%CI: 0.83-2.18]

*2. Apart from this major issue with standard errors, the meta-analysis has been meticulously carried out.*

We greatly appreciate this feedback and hope that the revised analyses also meet your expectations.

*3. The meta-analysis also assessed variation of effect in subgroups (effect modification) to see whether efficacy is affected by factors such as mosquito vector species, urbanicity etc. No such effects were evident, but the discussion should acknowledge that this analysis is ill-suited for such an investigation. These factors were specific to each study site with little or no variation within each study area. For example, effect modification by vector species cannot be assessed given that each vector is only present in either the one or the other site. Hence any potential effect modification would be entirely subsumed by differences between studies. Since effect modification is biologically plausible, there should be acknowledgement that the analysis had low power for detecting this if it existed.*

We agree with the reviewer and have added some additional context to the discussion that highlights that these analyses were ill-suited to identify effect modification, and that effect modification may still be an important consideration as more evidence becomes available. A section in the discussion has been added that reads as follows: “It is also important to note, that the analyses conducted were ill-suited to detect sub-group differences, due to so few studies being included, and each study having been conducted in different settings and with different dominant vector species. As effect modification is viably plausible, we emphasise that while we did not detect effect modification from any of the investigated subgroups, uncertainty remains, and effect modification may still be present. Future research is needed on these types of nets to investigate this concern.

*4. Combining the results of the three trials, two that used Royal Guard pyriproxyfen nets (cited above) and one that investigated the Sumitomo Olyset Duo pyriproxyfen net (Tiono et al), is of little value and possibly misleading given that the latter net has been withdrawn from the market some time ago by the manufacturers. Any conclusions based on a corrected analysis should make clear that the Royal Guard net (the only pyriproxyfen net currently available) failed to show superiority to standard nets in two independent trials. It should also be mentioned that Olyset Duo is not a WHO pre-qualified net, see* [*https://extranet.who.int/pqweb/vector-control-products/prequalified-product-list?field_product_type_tid=100&field_pqt_vc_ref_number_value=&title=&field_applicant_tid=&field_active_ingredient_synergis_tid=*](https://protect-au.mimecast.com/s/spZ5CMwGz2IEL1BOFP7MO4?domain=extranet.who.int)

Thank you for this insight. In relation to the *Sumitomo Olyset Duo* nets, this study was included as it did meet the eligibility criteria, however we will expand on the usefulness of this data in the discussion. In addition the Royal Guard net did show superiority to standard nets only at 1-year post intervention, while showing no superiority and not 2-year post-intervention.

We have added detail in the “Interventions and Comparisons” section identifying the limitations of the Olyset Duo nets. The updated section now reads as follows: “It is worth noting, that the Sumitomo Olyset Duo pyriproxyfen ITN has been withdrawn from the market and is not a WHO pre-qualified net.”

We have updated the discussion section to expand on how the conclusions drawn from this analysis may be limited. The updated section now reads as follows: “At 1-year post intervention, the evidence suggests that clusters that receive a chlorfenapyr-pyrethroid ITN (figure 4) or pyriproxyfen-pyrethroid ITN (figure 26) will result in a reduction of malaria case incidence compared to clusters that receive pyrethroid-only ITNs. Both findings were associated with high certainty of the evidence (Table 1 and 3). At 2-years post-intervention the evidence suggests that, compared to clusters receiving pyrethroid-only ITNs, clusters that receive chlorfenapyr-pyrethroid ITNs (figure 5) will also result in a reduction of malaria case incidence (Table 1), however, this was not observed in clusters that received pyriproxyfen-pyrethroid ITNs (figure 27, Table 3). These findings were associated with High and Low certainty evidence respectively.”

**Minor comments**

*1. Eligibility criteria should include proven pyrethroid resistance in malaria vectors in the study area (summary and p 26).*

Thank you for the consideration, however the eligibility criteria established for this review cannot be changed at this late stage. Additionally, these eligibility criteria were provided to the review team from experts in the WHO Global Malaria Programme.

*2. Introduction, first paragraph: cite figures on global malaria burden from most recent World Malaria Report, rather than outdated ones.*

Where appropriate, we have provided updated figures based on the most recent World Malaria Report.	We have updated the introduction to highlight this new information, the updated section now reads as follows: “Malaria presents a significant burden to global public health, with an estimated 247 million malaria cases in 2021^2^ Substantial progress has been made since 2000 in reducing global malaria cases from 80 cases per 1000 persons at risk to 57 per 1000 persons at risk in 2019. However, there was recently an increase in this metric to 59 per 1000 persons at risk observed in 2020^2”^

*3. Introduction, first paragraph: malaria case incidence should be expressed per 1000 persons at risk.*

This comment was addressed in response to the comment above.

*4. Also Introduction, first paragraph: It is incorrect to refer to the use of ITNs as a treatment strategy. It is a strategy for prevention (or control, not treatment).*

Thank you for this observation, we have amended the wording to identify ITNs as a prevention and not a treatment strategy. This changes has been made throughout the manuscript.

*5. Introduction, second paragraph, second sentence: this statement is dated: all nets are now factory treated long lasting insecticidal nets and those distributed through government or NGO agencies do not require re-treatments. Self treatment kits are generally no longer used.*

We have removed the sentence regarding self-treatment of nets using self-treatment kits from the revised manuscript.

*6. Page 25: There seems to be a muddle as to what are the same and what are different classes of insecticide. Some rephrasing is needed here.*

We have re-worded this section slightly and believe it to be clearer. We are trying to identify that of the two additional classes of ITNs, one class relies to the updated section now reads as follows: “The second class of ITNs, designed to kill resistant mosquitoes, consists of combinations of pyrethroid insecticides and other active ingredients. Belonging to this class includes ITNs treated with both a pyrethroid and piperonyl butoxide (PBO).^1^ PBO is a synergist that acts to inhibit the metabolic enzymes of the mosquito that work to detoxify (and therefore reduce effectiveness of) insecticides. The benefits to public health of these pyrethroid-PBO ITNs have been demonstrated, resulting in the WHO conditionally recommending that these nets be used, particularly in areas where pyrethroid-resistant mosquitoes are present.^1^ This class also provisionally includes ITNs that combine pyrethroids with other non-pyrethroid active ingredients (henceforth referred to as dual active ingredient nets, DAI). Studies on one DAI ITN, that combines alpha-cypermethrin (pyrethroid) and the pyrrole chlorfenapyr have recently demonstrated both entomological and epidemiological benefit.^4,5^

Finally, the third class of ITNs include those that have been designed to sterilize and/or reduce the fecundity of host-seeking insecticide-resistant mosquitoes. This class provisionally includes DAI ITNs treated with a pyrethroid insecticide and an insect growth regulator such as pyriproxyfen.”

*7. Page 26, objectives: As stated, these objectives go beyond what was actually addressed in this study, namely improved efficacy against malaria infection. The question whether something should actually be used rather than something else, is broader, including cost, coverage implications, acceptability, durability, etc. The objectives should be narrowed to what was actually investigated in this study, namely efficacy as a method of preventing malaria infection, compared to the current standard of care.*

Thank you for the suggestion, however as with the suggestion regarding the eligibility criteria, the objectives of the review questions as presented are unable to be changed at this stage. These review questions were explicitly provided by the WHO and serve the basis of the recommendations based on this evidence.

*8. Objectives (p 26): The term “long lasting insecticidal nets” should be used in both objectives – not just objective two.*

Thank you for this observation, we have added this detail to review question one as suggested.

*9. Page 27, line 2: State: population at risk for specified duration, or cases over person time.*

We have added the suggested change to the revised manuscript.

*10. Page 27, definition of mortality of adult female Anopheles should be widened to cover mortality caused by slow acting insecticides such as chlorfenapyr, since mortality due to exposure to these may take up to 72 hours.*

Thank you for this suggestion, however the outcome definitions used in this review are unable to be changed at this point. Additionally, these outcome definitions were approved by the WHO Global Malaria Programme as acceptable.

*11. Page 30, unit of analysis: “ the inflation of standard errors was not required as originally planned as per the protocol”. This does not mean it can be ignored, see point 1 of major comments.*

We have addressed this comment as per our new analyses described above.

*12. Page 31, Role of funding source. “The funder of the study had a role in the development of the protocol, the wording and development of the review questions, the interpretation of the final results and the development of this manuscript.” This seems rather unusual – Note to EDITOR: is it in line with the policy of the journal?*

We have removed this statement as per the Editors comments above. However, it is still true and will be made available in the online version in accordance with PLOS ONE standards.

*13. Page 32, Interventions: Nets are not 'coated' with insecticide. “Impregnated” would be a better description, since the chemical is intended to be gradually released to the surface of the fibre over the lifetime of the net.*

All instance of “coated” have been replaced with “impregnated” as suggested.

*14. Page 32, description of Accrombessi study: “ Both interventions were compared against each other”. This is incorrect: the interventions were only compared to control, not to each other. Not comparing to each other is a deliberate part of the design of the study to reduce the frequency of statistical testing. This also applies to the Mosha et al study which adjusted p-values to take account of multiple testing. Inventing additional tests post hoc invalidates this approach. The Olyset-PBO net was only compared to control, not to the other two interventions as stated in the text. The authors should correct this description. This does not prevent the comparisons being made in this secondary analysis of the data. In both studies (Accrombessi et al and Mosha et al) the comparator was the Interceptor net – in the text it appears that this is only the case for Mosha et al.*

The reviewer is correct and we thank them for bringing our attention to the wording in the original manuscript that did not make this clear. We have made changes throughout the manuscript (highlighted) that make it explicit that the interventions were only compared against a control pyrethroid-only ITN.

*15. Page 32, description of different nets: It would be better to give specs about chemical content in the same way for all nets, e.g. g/m2.*

Thank you for this suggestion, however we have reported the chemical content of the nets as they have been reported in their respective studies. We have not made a change in the revised manuscript regarding this suggestion.

*16. Page 35, Bias arising from randomisation process. Suggest rewording this to avoid restricted randomisation appearing to be muddled with allocation concealment -- the two have different objectives and are separate processes. Not doing the one does not affect the other and vice versa.*

Thank you for the suggestion. While we agree with the peer-reviewer that randomisation and allocation concealment are not the same thing, we have presented them as two separate concepts in the following description. Additionally, this is standardised wording associated with the Cochrane RoB 2.0 tool for cluster randomised controlled trials and we do not feel it would be appropriate to change. No changes have been made in the revised manuscript regarding this suggestion.

**Reviewer #2**

*Summary:
The number of incident cases averted per 1000 person-years at risk is a great way to frame the results, but an IRR would also be quite helpful for additional context. What were the actual incidence rates in the intervention clusters compared to the standard of care clusters? How big of a relative (i.e., percent) reduction is 190 and 145 fewer per 1000 person-years at risk?*

We have added the IRRs and associated 95%CI to the abstract as suggested. However, we feel that the information related to relative differences is available in Tables 1-4 and is better suited in these tables and not in the abstract.

*The interpretation could be better contextualized, as even though Dual AI ITNs do demonstrate consistent reductions in malaria case incidence and other outcomes across multiple comparisons, this statement is not explicit about what those comparison are. I think it is important to be specific and acknowledge that Dual AI ITNs provided better control of malaria than standard, pyrethroid-only ITNs in a variety of settings with pyrethroid resistant vectors.*

We believe that this is clear in the revised manuscript, in addition to the comment above, a section of the abstract has been re-written to read the following: “Compared to pyrethroid-only ITNs, clusters that received pyrethroid-non-pyrethroid DAI ITNs were associated with 305 fewer cases per 1000-person years (from 380 fewer cases to 216 fewer cases) (IRR = 0.55, 95%CI: 0.44-0.68). However, this trend was not observed in clusters that received pyrethroid-insect growth regulator ITNs compared to pyrethroid-only ITNs (from 280 fewer cases to 135 more) (IRR = 0.90, 95%CI: 0.73-1.13).”

*Summary of Finings Table: The descriptions of the Participants Follow-up (the first column) could be clearer. Specifically, how many studies are included in each assessment (for example, in the first row is it 3 total studies, 2 RCTs with 2000-person years plus another study with 61,183 participants?).*

Thank you for the suggestion and agree with the reviewer that the presentation may appear confusing and have tried to make this clearer in the revised manuscript. We also acknowledge that these tables have been presented in a standard way according to the GRADE approach. For each outcome, we clearly identify the number of studies that contribute to each outcome. It is then standard to provide the number of participants included in the analyses, however this data is unavailable for 1 study. This has now been clearly identified in Tables 1-4 of the revised manuscript.

*For the Anticipated absolute effects risk difference calculations (the last column), can you clarify if this is presented as a mean estimate number of cases averted per 1,000 with a 95% credibility interval? Also, maybe consider providing a brief explanation of the calculation and the source of the IRR used for this in the footnotes? It’s not immediately clear why sometimes the risk difference is straightforward (the difference in the event rates across study arms) and sometimes it is not (e.g., the first row of the chlorfenapyr-pyr ITNs compared to pyr-only ITNs table).*

The anticipated absolute effects are a simple way of presenting the IRR (or OR) in terms of absolute values, as per the GRADE approach. Based on the control risk (e.g. using row 1, Table 1 as an example) of 678 cases per 1000 person years. An IRR of 0.55 translates to 305 fewer cases [678-(678*0.55)=305.1. This is standard for the GRADE approach, and no changes have been made to the manuscript following this suggestion.

*Would be nice to standardize the number of significant figures presented throughout the table as well…not sure that carrying out the case incidence rates to the hundredths of a case per 1000 person-year is necessary.*

Thank you for the suggestion. The tables have been double checked, and the number of significant figures has been standardised in the revised manuscript. All percentages are presented with one significant figure, all other values are presented with two significant figures.

*Introduction:
This section needs to be proofread and cleaned up, and could be more focused than currently presented. Some examples of things to consider include:*

- *Is P. vivax usually considered one of the more virulent Plasmodium spp. parasites to infect humans?*

From our knowledge and cited sources P. vivax and P. falciparum are the most virulent parasites

- *ITNs (and vector control in general) are not treatment strategies, but prevention strategies.*

Thank you for this suggestion, we have addressed this feedback in relation to feedback from another peer-reviewer. Throughout the manuscript, ITNs are discussed as being a prevention strategy and not a treatment strategy.

- *I think the last sentence of the introductory paragraph is incomplete*

Thank you for the suggestion, in response to a piece of feedback the reviewer provided below, we have completely re-written the introduction to have a better and more coherent flow. We hope these changes have addressed this feedback.

- *I know that the classification scheme for ITNs has recently changed, but I do not think that it is correct to describe pyrethroid-only nets as requiring treatment with a treatment kit of periodic re-treatment…this might be confusing newer and older WHO terminologies.*

Thank you for raising this. This issue was also raised by a second peer-reviewer, and the sentence has been removed from the revised manuscript.

- *Based in part on the results reviewed in this meta-analysis, the public health value of DAI nets has been determined…I think it is appropriate to change the verb tense of this sentence and provide some more context. Perhaps “The public health value and incremental cost-effectiveness of Dual AI ITNs had not been established until recently.”*

We agree with the reviewer and thank them for their suggested phrasing. We have amended to the sentence in the introduction to the following: “The value to public health of DAI ITNs treated with both pyrethroids and insect growth regulators has not been established until recently.”

- *The final sentences of the last paragraph on the first page of the intro does not fit well with the first few sentences – there is no good transition from the discussion of how pyriproxyfen works to the problem of pyrethroid resistance.*

We have completely re-written the introduction following this feedback and believe that we have achieved a more consistent, and better fitting introduction. The revised changes taken place from lines 140 to lines 172 of the revised manuscript.

- *I’m not sure the review questions are appropriately framed, as they do not include a discussion of pyrethroid resistance in the local vectors “In areas with ongoing malaria transmission,” which were key considerations during each of the randomized trials reviewed. The question is a bit more complex than framed, and might be better if described like: “In areas with ongoing malaria transmission and pyrethroid resistance vectors, should nets treated with a pyrethroid and non-pyrethroid insecticide…”*
- *Also, there are some interesting policy implications in the language used: “should” dual AI nets be used “versus” standard nets. This would require a robust cost-effectiveness analysis, and a discussion of current WHO GMP recommendations on universal coverage targets. The questions are more typically framed like “Do Dual AI ITNs provide additional protection from malaria transmission, compared to standard pyrethroid-only ITNs, in areas of active malaria transmission and pyrethroid resistant vectors…”*

Thank you for the thoughts on the questions presented, however these questions are unable to changed at this stage. These questions were provided verbatim to the review team by the WHO Global Malaria Programme, and have been used to assist in the formation of recommendations in which these nuanced discussions between contextualised individuals can place.

*Results:
The Accrombessi paper from Benin has since been published and the text could be updated to reflect this.*

*It would be helpful if table 6 included a column with a basic description of the pyrethroid resistance status of the primary vector in each study location.*

Thank you for identifying this, we have amended the language used in the results to identify that this study has indeed been published.

*Discussion:
I think there may be a mix-up in the second paragraph, as Table 1 describes a high certainty of evidence that chlorfenapyr-pyrethroid ITNs will results in a reduction in malaria case incidence compared to pyrethroid-only ITNs, but the narrative describes this as only Moderate certainty.*

Thank you for this observation. These tables have been changed following comments from reviewer 1. We believe that all issues like this have been resolved in the revised manuscript. The updated certainty is high, and table 1 reflects this.
