# Supplementary material for: Effectiveness of dual active ingredient insecticide-treated nets in preventing malaria: A systematic review and meta-analysis
Source: PLoS One. 2023 Aug 16;18(8):e0289469. doi: 10.1371/journal.pone.0289469 (PMC10431665; doi:10.1371/journal.pone.0289469)
Supplement: Attachment — Submitted filename: Response to Reviewers.docx [file pone.0289469.s004.docx]

**Response to Reviewers – Accompanying Manuscript PONE-D-23-09253R1**

**Effectiveness of dual active ingredient insecticide-treated nets in preventing malaria: a systematic review and meta-analysis**

**Reviewer #1**

*Thank you for responding to my previous comments so thoughtfully and for having addressed them where appropriate. I have only two remaining points that should be dealt with before publication:*

*1. Having taken care of the inflation of standard errors and 95% CIs to account for the within-cluster correlation of responses after my earlier comments relating to this (and now reflecting this in the revised results), I don't see this mentioned anywhere in the methods section of the manuscript. Please add a sentence stating that calculation of 95%CI took account of the clustered nature of the data.
Apologies if it is there and I have missed it.*

We thank the reviewer for identifying this oversight in our methods. We have added the following sentence to lines 333-334 of the “data synthesis and meta-analysis sections of the revised manuscript. “Calculation of 95% CIs took account of the clustered nature of the data where appropriate.”

*2. Overall there are a very large number of endpoints in this paper and it is therefore possible that one or two results may be due to chance, despite the fact that these data derive from Randomised trials. This is particularly a strong possibility were one endpoint result is contrary to the pattern of results for a particular intervention. For example, the pyrethroid-insect growth regulator (pyriproxyfen) dual active ingredient vs pyrethroid only net comparison shows 95% CIs overlapping 1 (i.e. non significant) for all but one endpoint, namely malaria case incidence after 12 months. It is highly implausible that this net would be superior to pyrethroid only nets in this one particular way but otherwise no better than the standard net. A more plausible interpretation is that this result is a chance finding and there is little evidence that pyriproxyfen DAI nets are superior to standard nets (the null hypothesis). There should thus be some caution expressed that multiple testing will lead to chance findings and it is therefore important to interpret results not in isolation but in relation to other endpoints for the same product.*

We agree with the reviewer and have added a few sentences regarding this point to the limitations section of our discussion. The following sentences have been added to lines 955-960 of the revised manuscript. “Finally, we argue that while these findings should be interpreted carefully within the context of a guideline panel, they should also be interpreted in relation to other endpoints assessed regarding the same comparison. For example, caution should be taken when interpreting the results presented in figure 17 (pyriproxyfen-pyrethroid ITNs versus pyrethroid-only ITNs – 12 months) in isolation from the results presented in figure 16 and 18, as this result may suggest these DAI ITNs have superiority over pyriproxyfen-ITNs that may not exist when considering the entire body of evidence.”

**Reviewer #2**

*The revisions have addressed many of the comments and concerns raised by the reviewers and editors, particularly the re-calculation of the confidence intervals, the reframing the prevalence results as odds ratios, and the re-interpretation of many of the conclusions in light of the updated analysis.

Most of the revised text is also easier to understand and improves the readability of the paper.

The methods, analysis, and assessment of heterogeneity and risk of bias all seem robust and well carried out. Unfortunately, I am still concerned that the review questions are not framed appropriately and the conclusions are too broad as presented, and can not recommend publication as written.*

*Appreciating that the GMP provided the research questions to the review team, they are still poorly framed and not addressed by the studies currently available.*

*For question 1, the available data only address transmission settings with pyrethroid resistant An. gambiae s.l. and/or An. funestus s.l. in Africa. The question, and the conclusions of the paper, need to be revised to reflect this in order to recommend publication. Also, I believe that WHO PQ prefer the original term used “nets treated with,” or the term “insecticide treated nets (ITNs) utilizing a pyrethroid…”compared to the the revised phrase “long last insecticidal nets treated with….”

For question 2, the question as written is simply not answerable with the data available. It is comforting to see that no recommendation or conclusion relative to this question is presented in the paper, and there is some acknowledgment of the nuance required when interpreting the results in the context of malaria control programming. However, the second research question should be removed or, alternatively, acknowledged as not yet answerable while highlighting the knowledge gaps that need to be addressed in order to do so (e.g., cost effectiveness – in the face of a volatile ITN market with shifting net prices and new tools on the horizon, and a need to revisit current WHO GMP policy recommendations with regard to universal coverage targets).*

We thank the reviewer for their time reviewing this manuscript and their suggestions to improve the quality of the review. While we have actioned some changes (outlined below), we will not be removing or adjusting the questions as presented in the review. These questions were provided to the review team from the WHO Global Malaria Programme and to change them or remove them at this stage of the review process would not be appropriate. We hope the reviewer can appreciate and accept this. We will, however, change the wording of the questions from “long lasting insecticidal nets…” to “insecticide-treated nets” as this does not change the intent of the original question posed.

We do agree with the review that more explicit mention can be made to the limitations of the review questions and how they relate to the conclusions of the paper. Firstly, we have made changes to the sentence from lines 945-947 that clarify that the data comes from high-transmission settings with pyrethroid resistant *An. Gambiae s.l* and/or *An. Funestus s.l* in Africa and therefore generalisability of these results may be hampered. “However, this does suggest limitations to the transferability of this data as the results have all come from high-transmissions settings with pyrethroid resistant An. Gambiae s.l and/or An. Funestus s.l vectors from Africa.”

We have also added an additional sentence to the limitation’s sections of the discussion regarding the second review question. This change has been added from lines 960-962. “Finally, the second review question that was initially asked was unable to be answered in this review with the data made available to the review team, as such, no conclusions have been made regarding this data.”
